# Body-Shaping Membrane to Regenerate Breast Fat by Elastic Structural Holding

**DOI:** 10.34133/research.0137

**Published:** 2023-05-10

**Authors:** Hye-Seon Kim, Jeongeun Park, Hyun-Su Ha, Sewoom Baek, Chan Hee Lee, Kyubae Lee, Suji Park, Jueun Kim, Se Won Yi, Hak-Joon Sung

**Affiliations:** ^1^ Department of Medical Engineering, Graduate School of Medical Science, Brain Korea 21 Project, Yonsei University College of Medicine, Seoul 03722, Republic of Korea.; ^2^ TMD LAB Co. Ltd., 6th Floor, 31, Gwangnaru-ro 8-gil, Seongdong-gu, Seoul 04799, Republic of Korea.

## Abstract

Tissue regeneration requires structural holding and movement support using tissue-type-specific aids such as bone casts, skin bandages, and joint protectors. Currently, an unmet need exists in aiding breast fat regeneration as the breast moves following continuous body motion by exposing the breast fat to dynamic stresses. Here, the concept of elastic structural holding is applied to develop a shape-fitting moldable membrane for breast fat regeneration (“adipoconductive”) after surgical defects are made. The membrane has the following key characteristics: (a) It contains a panel of honeycomb structures, thereby efficiently handling motion stress through the entire membrane; (b) a strut is added into each honeycomb in a direction perpendicular to gravity, thereby suppressing the deformation and stress concentration upon lying and standing; and (c) thermo-responsive moldable elastomers are used to support structural holding by suppressing large deviations of movement that occur sporadically. The elastomer became moldable upon a temperature shift above *T*_m_. The structure can then be fixed as the temperature decreases. As a result, the membrane promotes adipogenesis by activating mechanotransduction in a fat miniature model with pre-adipocyte spheroids under continuous shaking in vitro and in a subcutaneous implant placed on the motion-prone back areas of rodents in vivo.

## Introduction

Implantable medical devices should enable adhesion (structural holding) and movement (elasticity) of neighboring tissues [1,2]. Hence, the device is designed to fit into organ shapes for scaffolding upon implantation [3], and the device material properties are tuned to match the stiffness and movement of target tissues, resulting in different properties for bone, blood vessels, muscle, and fat [4,5]. The concept of elastic structural holding is seen in some medical aids, such as bone casts, skin bandages, and joint protectors [6]. The major functions of these aids are (a) to tightly hold the neighboring tissues surrounding a defect so that the tissues adhere to each other to facilitate the healing process by preventing structural disintegration and (b) to preserve tissue motion so that organ-specific movement is not hampered, thereby accelerating the healing process. Continuous advances have been made in the patient-specific design and properties of implantable devices [7]. Moreover, the design and material of implantable devices have been tuned to support the mechanical properties of target tissue because the devices can promote regeneration upon synchronization of structure–function relationship with the surrounding tissue [8,9]. However, aids that can enable elastic structural holding through a combination of the device material and design functions, especially for fat regeneration, which is a relatively unexplored area, remain elusive.

Fat regeneration is required for breast reconstruction after surgical treatment of patients with cancer [8]. As breast cancer is the most prevalent cancer in women worldwide, with continuously increasing morbidity [9], surgical demand justifies breast fat regeneration as a target model of this study. Silicone has been used as a standard material for breast implants because of its long-term durability and easy fabrication [10]. However, as silicone exhibits poor structure-fitting ability and shape fixity, it limits the ability of elastic structural holding to promote breast fat regeneration under implantation and is susceptible to biofilm formation [11,12]. The function of elastic structural holding is particularly important because breasts move unexpectedly with large deviations in response to body motions, and the movement varies depending on their size, shape, and age. In this regard, user-defined shape molding and elastic synchronization with fat movement should be enabled by combining material properties and implant design as a new paradigm of development.

Mechanotransduction mechanisms convert the effects of body movement on cellular signaling for either regeneration or destruction [13,14]. The stable activation of pro-adipogenic mechanotransduction by suppressing large deviations in breast motion can be proposed as a major mediator to promote fat regeneration by elastic structural holding. As a result, mechanotransduction-induced signaling can direct cell shape, behavior, and fate for regenerative actions [15,16]. Adipose cells in breast fat are particularly subject to this effect because their movements are affected by dynamic movement and mechanical loading in cooperation with gravity-associated factors, including body weight loading and weight bearing [17]. Hence, when abnormal fat movements are suppressed to produce consistent motion, adipose cells activate pro-adipogenic mechanotransduction with (a) actin contraction (length shortage), (b) increased cell–cell adhesion, (c) suppression of YAP expression in nuclei with movement to cytosol areas, and (d) increased PPARγ expression [16,18–20].

In this study, the elastic structural holding was enabled using a new concept of the breast membrane, thereby promoting adipogenesis. While the current devices can simply promote the healing process by adhering to a target tissue or supporting the motion, the membrane enables structural holding with operation of similar mechanical properties to the breast tissue. The design is established by using honeycomb structure [strut (−)] with the addition of a strut [strut (+)] in the direction perpendicular to gravity. The unique strut (+) design enables the management of stress distribution to low levels throughout the entire membrane structure by suppressing stress concentration upon lying and standing based on computational analyses using the finite element method (FEM). The design stabilizes the motion of breast tissue through structural integration with improvement. A thermo-responsive polymer provides elasticity and user-defined molding capability so that the membrane can hold the breast shape while preserving elastic motion. The molding capability and elastic structural holding function of strut (+) appreciably suppress large deviations in breast-mimetic motion owing to its higher modulus under tensile and compression conditions compared to strut (−).

The quality and composition of breast fat vary depending on each patient, which limits the consistent examination of pro-adipogenic activities upon sufficient proliferation of adipose cells [21,22]. Hence, a hemispheric fat miniature was used by culturing pre-adipocyte spheroids in a gelatin gel using a 3-dimensional (3D) printed mold due to the matched characteristics of adipose tissue and breast movement with the support by gelatin gel under shaking culture [21,23–25], thereby enabling the in vitro examination of strut (+) effect on elastic structural holding. Also, as the fat regeneration can be determined upon partial defecting of miniature, the hydrogel filling of defect was approached to prevent the breast shape deformation under shaking and animal movement. In this study, a hydrogel of methoxy poly (ethylene glycol)-block-poly(ε-caprolactone) (mPEG-PCL) was chosen because of the injectable property at the body temperature with the solution–gel transition. Also, this hydrogel is biodegradable so that the switch of space filling by new fat regeneration can be supported. This hydrogel is biocompatible as approved by Food and Drug Administration (FDA) [26–29] and has exhibited nontoxic side effects in the long-term implantations [30–32]. When these fat miniatures were subjected to shaking on a rocker and implanted subcutaneously into the motion-prone back of mice, strut (+) promoted fat regeneration by stably activating pro-adipogenic mechanotransduction. Hence, the results suggest a paradigm-shiftable device to accelerate breast fat regeneration after partial surgical defects.

## Results

### Design and computational analysis of strut-guided advantages in membrane

**Fig. 1.**
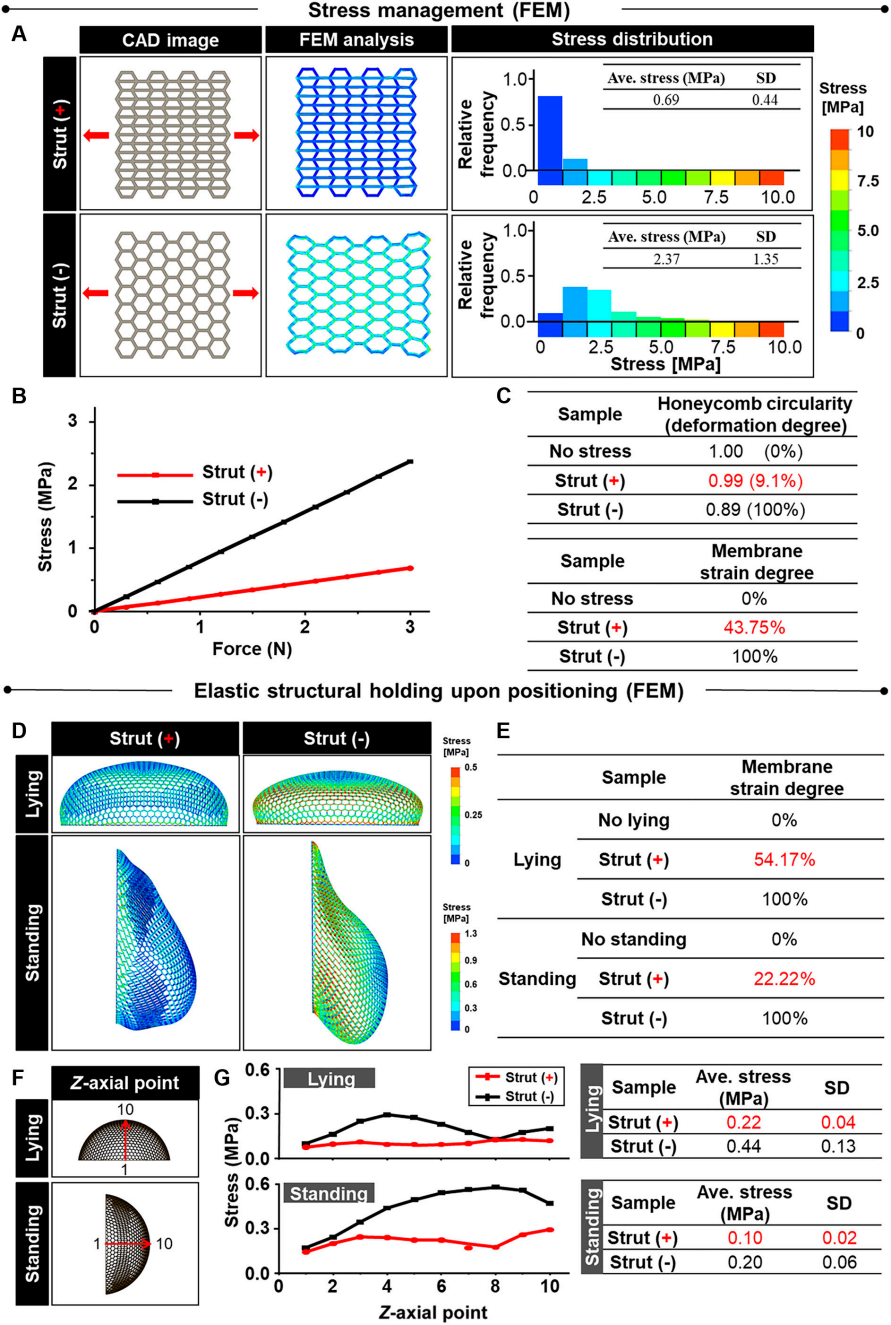
Strut-guided stress management and elastic structural holding of the membranes in strut (+). The elastic properties and structural holding function of a membrane are derived from the polymer and design, respectively. Their synergistic actions in the responses of the membranes to the breast movement under a daily pressure (3 N) from standing and lying [48] were determined by FEM analysis of 2D flat (A to C) and 3D hemisphere (D to F) membranes. (A) The test designs (CAD) were set by adding struts [strut (+)] into the honeycomb structure of a flat membrane [strut (−)]. Strut (+) manages the stress distribution effectively to the lower ranges (average 0.69 in 0 to 2.5 MPa) over the entire membrane, unlike strut (−) (average 2.37 in 0 to 6.125 MPa). This result is supported (B) as strut (+) maintains lower levels of stress than those of strut (−) under the same incremental force (0 to 3 N). Owing to the consequent effect of elastic structural holding, (C) strut (+) suppressed the deformation of the honeycomb structure to 9.1% (circularity) and 43.75% (strain), when each degree was set from 0 (no motion stress) to 100% [strut (−)]. Under daily pressure, the 3D hemisphere model analysis exhibited the lower range of stress distribution by strut (+) over the entire structure in both lying and standing motions compared to strut (−) according to (D) FEM analysis. As a result, (E) strut (+) suppressed the deformation of honeycomb circularity to 54.17% (lying) and 22.22% (standing), when each degree was set from 0 (no motion stress) to 100% [strut (−)]. (F) For the 3D hemisphere model, the stress distribution to the 10 *z*-axial points was analyzed because the *z*-axial pressure is often applied daily by lying and standing. The point is incremental from the bottom (=1) to the top (=10) of hemisphere in each position in the 2D images. (G) The strut (+) membrane maintained the lower levels of stress through the 10 points, with a 50% reduction in average stress in the lying and standing positions compared to the strut (−) membrane.

As the elastic property and structural holding function of membranes are dependent on the polymer and design, respectively, these unique device characteristics were examined by computational analysis (Fig. 1 and Fig. S1). The adipoconductive membrane was designed by adding struts [strut (+)] into the honeycomb structure [strut (−)]. The test designs [computer-aided design (CAD)] and FEM analysis were set by strut (+), strut (−), and square as a form of 2D flat membrane (Fig. 1A and Fig. S1A). In response to the stretching deformation (red arrows), only strut (+) maintained the structural stability among the candidates. Strut (+) and square managed the stress distribution effectively in the lower range [strut (+): average 0.69 in 0 to 2.5 MPa and square: average 0.60 in 0 to 2.48 MPa] over the entire membrane compared to strut (−) (average 2.37 in 0 to 6.13 MPa) (Fig. 1A and Fig. S1B). In agreement with this result, strut (+) and square maintained lower levels of stress than strut (−) under the same incremental force (0 to 3 N) (Fig. 1B and Fig. S1C). These results justified strut (+) as the design of adipoconductive membrane to maintain the structural stability upon stretching with the maintenance of low stress distribution. Furthermore, as an indication of the elastic structural holding effect, strut (+) suppressed the deformation of the honeycomb structure to 9.1% (circularity) and 43.75% (strain) when each degree was scaled from 0 (no motion stress) to 100% [strut (−)] (Fig. 1C).

After CAD modeling of the membrane, FEM was used to analyze stress management at a lying or standing position (Fig. S2). The stress management of membrane with consequent resistance to structural deformation under an average daily pressure (3 N) in the range of 1.3 to 5.6 N upon standing and lying was reported previously [35,36]. The results from the 2D flat models were confirmed, as the lower range of stress distribution was managed by the strut (+) membrane over the entire structure in both lying and standing positions unlike in the strut (−) membrane (Fig. 1D). Consequently, the strut (+) membrane suppressed the deformation of honeycomb circularity to 54.17% (lying) and 22.22% (standing) when each degree was set from 0 (no motion stress) to 100% [strut (−)] (Fig. 1E). In particular, for the 3D hemisphere model, the stress distribution to the 10 *z*-axial points was analyzed because the *z*-axial pressure is often applied daily by lying and standing (Fig. 1F). The point was incremental from the bottom (=1) to the top (=10) of hemisphere in each position, and the 2D images were analyzed due to the limitation of program capability to analyze the too many numbers of node when 3D models were used. The higher stress level of strut (−) varied at each *z*-axial point compared to strut (+) as seen by the close point (=1) in both positions and overlapping (=8) in lying. Overall, the strut (+) membrane maintained the lower levels of stress through the 10 points, with a 50% reduction in average stress in the lying and standing positions compared to the strut (−) membrane (Fig. 1G). These computational analyses provide precalculated insights into the advantages of strut (+) regarding stress management and structural holding under the daily pressure.

### Membrane production for user-defined moldability and elastic structural holding

As a membrane material, a thermo-responsive elastic polymer [6arm-polycaprolactone-poly glycidyl methacrylate (6-arm 96% PCL-*co*-04% PGMA)] was first synthesized and characterized (Fig. S3). Successful polymer synthesis was confirmed by analyzing the chemical structure using proton nuclear magnetic resonance (^1^H-NMR) spectrometry (Fig. S3A). The crosslinking of polymer was verified as *T*_m_ decreased (Fig. S3B) as opposed to the glass transition temperature (*T*_g_) in differential scanning calorimetry (DSC) analysis (Table S1). The cross-linking reduced Young’s modulus by over 80% to 5.24 ± 0.2 MPa at 45 °C and to 10.83 ± 1.68 MPa at 40 °C compared to that before molding at room temperature in DMA analysis (Fig. S3C). Hence, the transition temperature (*T*_t_) was determined to be 45 °C (*T*_m_ post-crosslinking) due to the moldable flexibility of polymer chains. The maximum tensile strength in the stress–strain curve is aligned with the results of the FEM analysis when the mechanical properties of the polymer film were analyzed using a universal testing machine (UTM) (Fig. S3D). Compared to silicone (~2 MPa), more stress (>10 MPa) was required to deform the membrane under a 900% strain, indicating the membrane’s potential to enable elastic structural holding (Fig. S3E and Table S2).

The thermo-responsive polymer was stable and biocompatible as determined by the following tests. First, the polymer exhibited about 35% weight loss even for 1 year compared to the less than 1% weight loss of silicone when the results of accelerated degradation test were analyzed following the American Society of Testing and Materials (ASTM) international standard 1980 [33] (Table S3). This slow degradation indicates a supportive role of fat regeneration based on the tissue engineering principle. Second, a series of biocompatibility tests [(a) cytocompatibility, (b) biofilm formation, (c) intracutaneous reactivity, and (d) blood compatibility] was carried out by the principles of Good Laboratory Practice (GLP; ISO 10993-10). (a) The polymer was cytocompatible when cell viability was tested through a CCK-8 assay by varying the elution concentration in the culture medium after incubation with the polymer for 3 d at 37 °C (Fig. S3F). (b) As biofilm formation is a prevalent issue for implantable materials, including silicone, the superior suppression of *Pseudomonas aeruginosa* adhesion by the polymer over silicone was demonstrated after incubating the bacteria on test films at 37 °C for 24 h and performing SEM (Fig. S3G). (c) The intracutaneous reactivity was determined by scoring the degree of local irritation in the cranial end of the rabbit at 0, 24, 48, and 72 h after intracutaneous injection of polymer extract in saline or cottonseed oil (Fig. S4 and Tables S4 to S6). The results confirm that the polymer is a nonirritable material in the body as neither edema, erythema, nor eschar was observed in the saline-injected sites by maintaining no meaningful differences with the oil injected sites. (d) When hemolysis was tested (Tables S7 and S8), −0.66% of the −0.77 hemolytic index validates that the thermo-responsive polymer is clearly hemocompatible considering 0.11% of polyethylene (negative control) and 16.85% of plasticized polyvinyl chloride pellet (positive control) with a consequent index range of 0 to 16.74. Hence, the thermo-responsive polymer was considered for the follow-up biological experiments.

**Fig. 2.**
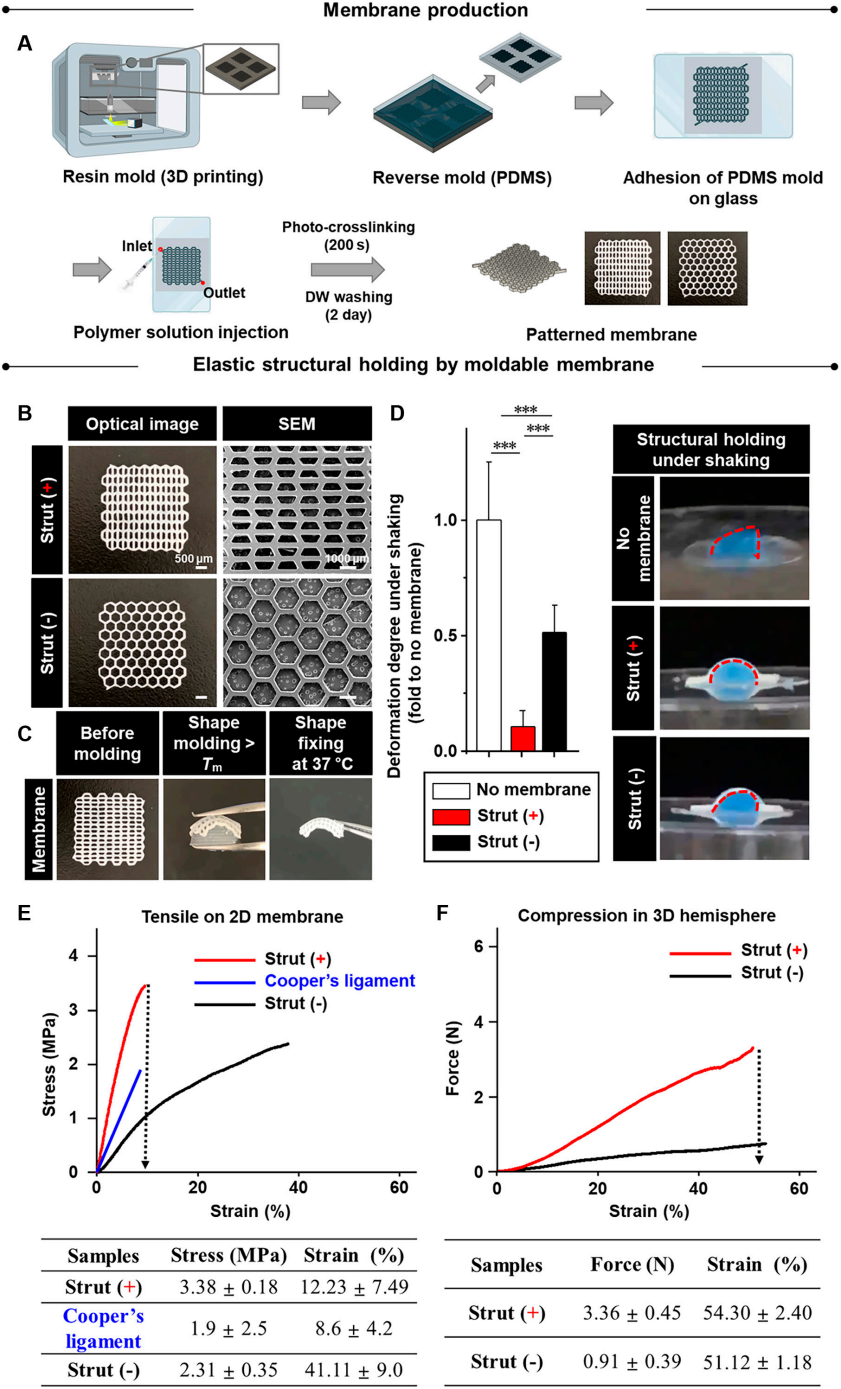
Membrane production for user-defined elastic shaping and strut-guided structural holding. (A) To produce test membranes, a resin mold was 3D printed, and a reverse PDMS mold was cast using the resin mold. It was then adhered onto a glass plate. The polymer solution was injected into the PDMS mold, which was then exposed to UV crosslinking for 200 s and washed 2 times. As a result, patterned membranes (strut +/−) were produced as shown (B) in the optical and SEM images. Because of the thermo-responsive elastic switch, (C) the flat membrane was molded to a breast shape upon a temperature shift above *T*_m_ (45 °C). The shape was then fixed at 37 °C through the release of heat energy. Owing to the synergistic action between material properties and design, (D) the elastic structural holding of a breast shape under shaking was confirmed in the strut (+) membrane, which preserved uniform motion (images) by minimizing the heterogeneous shape deformation (graph) as seen in the other groups. This unique property is promoted by strut-guided stronger resistance to (E) tensile deformation of 2D membrane and (F) to compressional deformation of the 3D hemisphere membrane, compared to the Cooper’s ligament and/or the strut (−) membrane. Data are mean ± SD (*n* = 7 for all experiments).

The test membranes were generated by 3D printing a resin mold, which was then used to cast a reverse PDMS mold. The mold has then adhered onto a glass plate (Fig. 2A). After injecting the polymer solution into the PDMS mold, the sample was exposed to ultraviolet (UV) crosslinking for 200 s and washed for 2 d to produce strut (+/−), as shown in the optical and scanning electron microscopy (SEM) images (Fig. 2B). As the movement of the polymer chains increases with increasing temperature, the elastic switch of the membrane enabled molding to a breast shape upon a temperature shift above *T*_m_ (45 °C) after storage for 5 s (Fig. 2C and Movie S1). In this way, the thermo-responsive moldable elastomers are suitable for the use in humans by quickly molding above *T*_m_ (45 °C) so that heat damage can be minimized. As another option, the elastomer can be slowly molded at 40 °C for 300 s slightly above body temperature and under *T*_m_ (Movie S2) because Young’s modulus was reduced to 10.83 ± 1.68 MPa by over 80% compared to that before molding at room temperature (Fig. S3C). Alternatively, *T*_m_ of elastomer can be adjusted by adjusting the crosslinking degree upon alteration of the ratio between PCL and PGMA as reported previously [34–36]. The crosslinking decrystallizes the polymer, resulting in reduction of *T*_m_ because the endothermic heat energy decreases.

The shape was then fixed at 37 °C by releasing heat energy. The synergistic action between the material and design results in the elastic structural holding of the breast shape by strut (+) under shaking conditions as uniform motion is preserved by minimizing heterogeneous deviations of the shape deformation, unlike in other test groups (Fig. 2D and Movies S3 to S5). Moreover, this unique property was enforced by the strut-guided stronger resistance to tensile deformation of the 2D membrane (Fig. 2E) and to the compressional deformation of the 3D hemisphere membrane (Fig. 2F) than Cooper’s ligament and/or strut (−). Strut (+) suppresses large deviations in breast motion by providing enforced resistance to tensional and compressional deformations through elastic structural holding.

### Fat miniature under shaking with strut-guided pro-adipogenesis

**Fig. 3.**
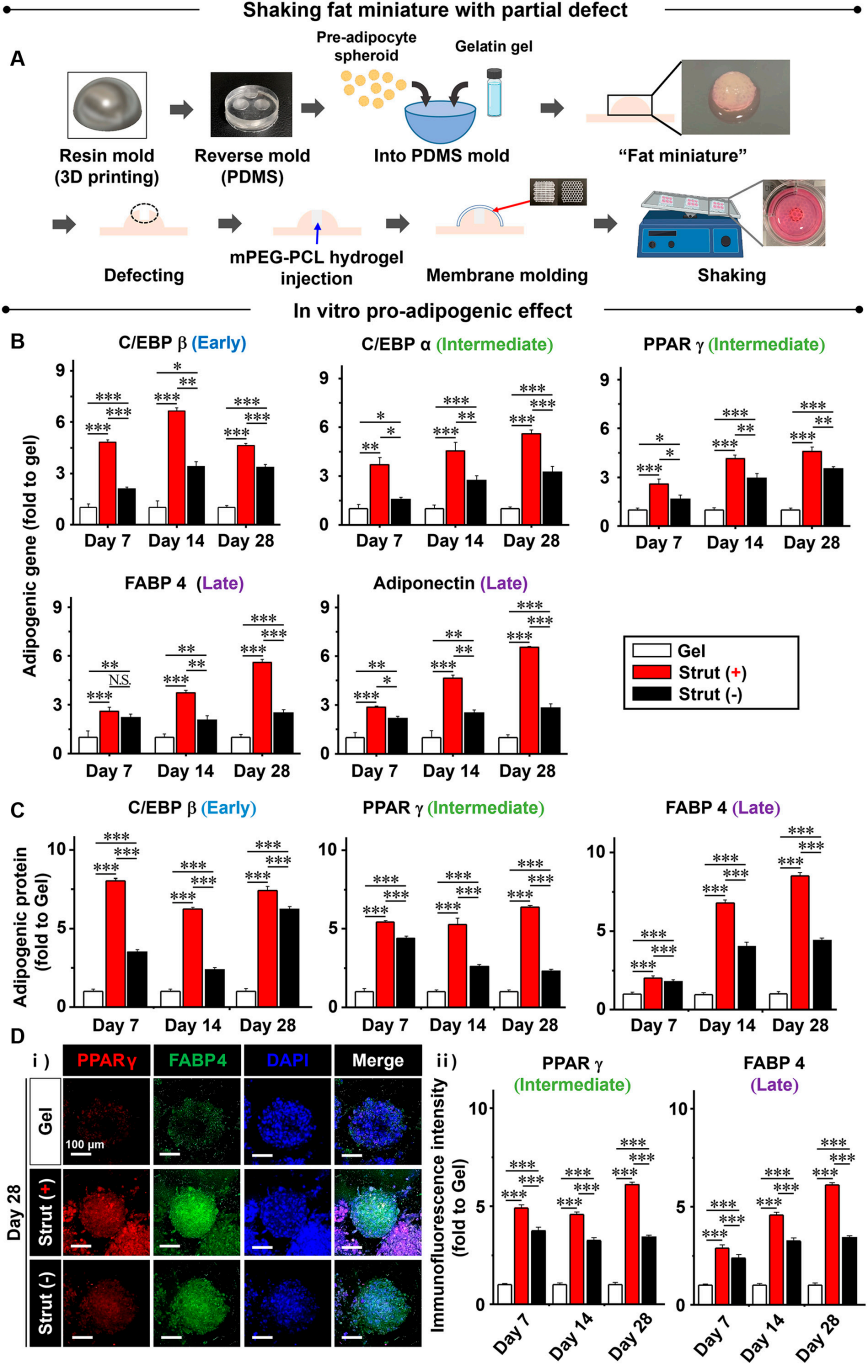
Fat miniature under shaking with strut-guided pro-adipogenesis. For the following in vitro experiments, (A) artificial breast fat as a form of miniature (fat miniature) was produced. A resin mold was 3D printed to produce a reverse PDMS mold. Pre-adipocyte spheroids with gelatin gel were then placed in the mold, which became a fat miniature following cell culture. Defects were made in the fat miniature to model breast regeneration, and mPEG-PCL hydrogel was injected into the defects to serve as matrix filing. The defect was then covered by the molding test membranes. When the test groups (no membrane and strut +/−) were shaken on a rocker following treatment with adipogenic induction medium for 28 d, the strut-guided elastic holding promoted (B) marker gene expression of pro-adipogenesis more significantly than no membrane and strut (−) as determined by qRT-PCR. The genes include the early (C/EBPβ), intermediate (C/EBPα and PPARγ), and late (FABP4 and adiponectin) pro-adipogenic markers. The results are aligned with the protein expression of the same markers (C) by quantitative analysis of a Western blot and by (D) (i) immunofluorescence staining by confocal imaging with (ii) quantitative analysis. Data are mean ± SD. *P < 0.05, **P < 0.01, and ***P < 0.001 between the lined groups (*n* = 3 for all experiments).

The artificial breast fat was produced in the form of a miniature (fat miniature) through the following process (Fig. 3A). A resin mold was 3D printed to produce a reverse PDMS mold. Pre-adipocyte spheroids with gelatin gel were then placed in the mold, which became a fat miniature after 14 d of culture. The fat-like characteristics of miniature were validated as the gene expression of adipose-specific factors (PPARγ and adiponectin) [37] increased compared to 2D and 3D suspension cultures (Fig. S5A), which was supported by the histological examination (Fig. S5B). The miniature was filled with defects to model breast regeneration. The mPEG-PCL hydrogel was injected into the defect to serve as matrix filing to prevent the shape deformation under the motion pressure. The defect was then covered with molding test membranes. The successful synthesis of the mPEG-PCL hydrogel as an injectable filler was confirmed by ^1^H-NMR spectroscopy using chloroform-d_6_ (CDCl_3_) (Fig. S6A). *T*_m_ of the hydrogel appears at approximately 40 °C as a typical property of mPEG-PCL obtained through DSC analysis (Fig. S6B and Table S9). The phase diagram of the hydrogel was produced by analyzing the sol–gel phase transition at various test concentrations (Fig. S6C). The cytocompatibility of mPEG-PCL hydrogel was verified by comparing the compatible cytotoxicity of L929 cells to that of no extract (0%) using the CCK-8 assay (Fig. S6D), indicating a promising function to support the defect regeneration by cell proliferation.

The test groups (no membrane and strut +/−) were shaken on a rocker as a model of breast motion in an adipogenic induction medium for 28 d. As a result, the marker gene expression of pro-adipogenesis significantly increased from no membrane to the strut (−) membrane and further to the strut (+) membrane because of the strut-guided elastic holding, as determined by quantitative reverse transcription polymerase chain reaction (qRT-PCR) (Fig. 3B). The genes included early (C/EBPβ), intermediate (C/EBPα and PPARγ), and late (FABP4 and adiponectin) pro-adipogenic markers. The results were supported by the protein expression of the same markers when a Western blot (Fig. 3C and Fig. S7) and confocal images (Fig. 3D and Fig. S8) were quantitatively analyzed. In contrast to the shaking condition, when fat miniatures were cultured under static conditions using adipogenic induction medium for 28 d, the effects of the test membrane types were not differentiated, as no change in adipogenesis of fat miniatures was observed among the test groups (Fig. S9) according to the qRT-PCR analysis of pro-adipogenic markers (C/EBPβ, C/EBPα, PPARγ, FABP4, and adiponectin).

### Effect of body motion on the fat miniature implant with strut-guided pro-adipogenesis

**Fig. 4.**
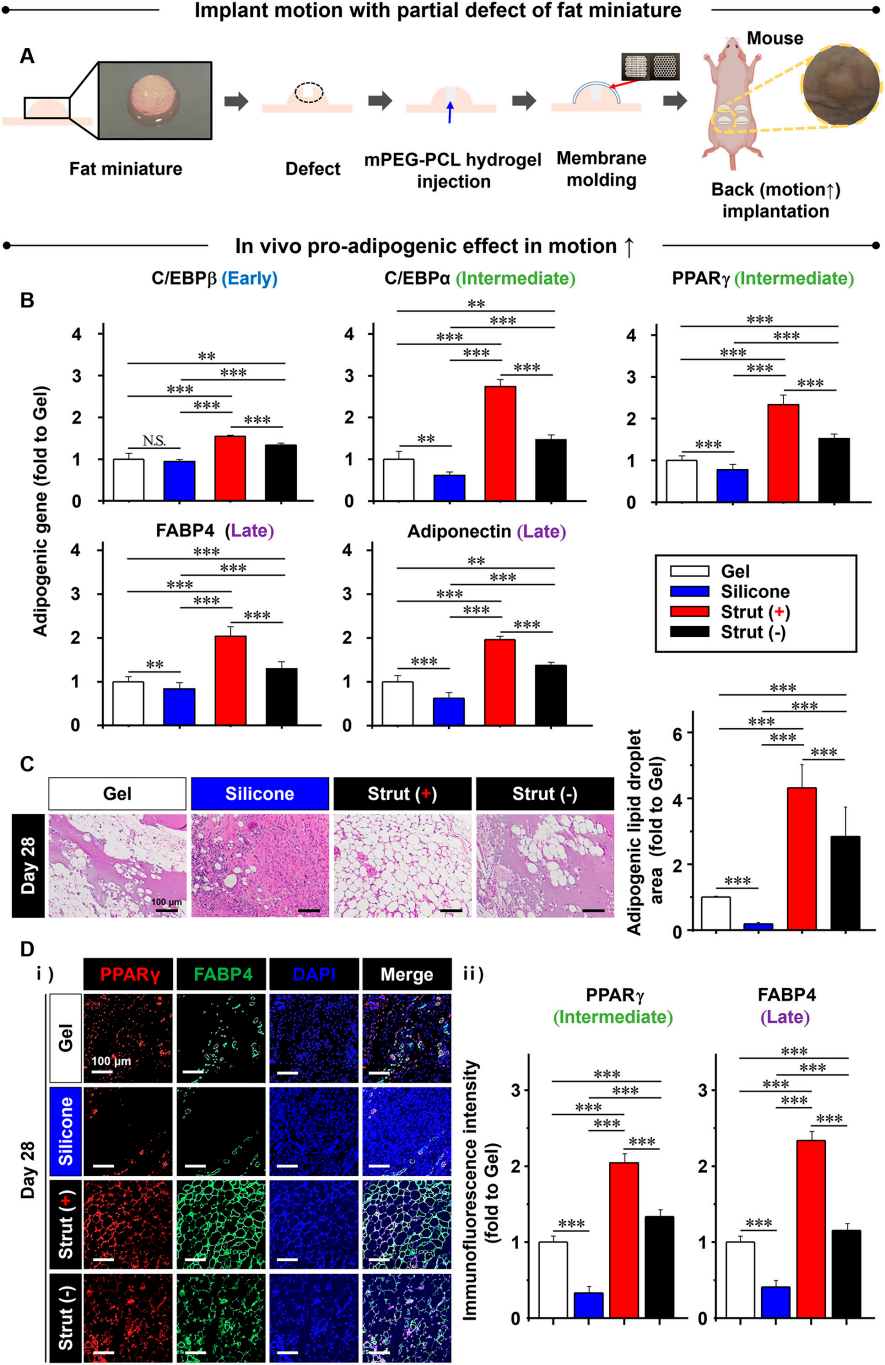
Body motion effect on the fat miniature implant with strut-guided pro-adipogenesis. For the following in vivo experiments, (A) the fat miniature was defected to model breast regeneration, and mPEG-PCL hydrogel was injected into the defect to serve as matrix filing. The defect was then covered with the molding test membranes. The test groups included gel (no membrane), silicone (commercial control), and strut (+/−). Test samples were implanted into the subcutaneous back of each nude mouse for 28 d so that the samples could be affected by the more active motion of the back compared to the abdomen side (Fig. S8). The strut-guided elastic holding promoted (B) marker gene expression of pro-adipogenesis significantly from gel and silicone to strut (−) and furthermore to strut (+) as determined by qRT-PCR. The genes included the early (C/EBPβ), intermediate (C/EBPα and PPARγ), and late (FABP4 and adiponectin) pro-adipogenic markers. (C) These results are supported by the greater adipogenic morphology (lipid droplet area) of strut (+) compared to the other groups, including the silicone, in which tissue fibrosis was prevalent. (D) The results agree with the protein expression of intermediate (PPARγ) and late (FABP4) pro-adipogenic markers (i) through immunofluorescence staining by confocal imaging with (ii) a quantitative analysis. The fibrotic tissue formation of silicone hinders adipogenesis. Data are mean ± SD. *P < 0.05, **P < 0.01, and ***P < 0.001 between the lined groups (*n* = 6, for all experiments).

Fat miniatures were covered by strut (+) with sterilization using ethylene oxide (EO) gas, followed by implantation into the subcutaneous back of nude mice for 28 d. The successful invasion of in vitro cells and in vivo tissues into the defect sites was confirmed as the hydrogel degradation generated the space to fill after 28 d (Fig. S10). Also, no visible inflammatory response was observed by H&E staining (Fig. S11A) as supported by the negligible expression of tumor necrosis factor-α (TNF-α) in immunocytochemistry (Fig. S11B). Furthermore, bacterial infection was not observed by crystal violet staining compared to silicone (Fig. S12A), followed by quantitative analysis (Fig. S12B). Motion frequency is an important factor in differentiating the effects of elastic structural holding. Hence, the abdomen of rats or the back of mice was selected to compare the effects of motion frequency because the back appears to move more frequently than the abdomen for rodents. The motion frequency of the abdomen was 6 times less than that of the back according to the pedometer recording (Fig. S13A). The adipogenic effect of low motion frequency was determined and compared to that of high motion frequency (Fig. 4).

The back implantation was preceded by adding defects to the fat miniatures to model breast regeneration, and mPEG-PCL hydrogel was injected into the defect to serve as matrix filing. The defect was then covered with the molding test membranes (Fig. 4A). The test groups included gel (no membrane), silicone (commercial control), and struts (+/−). Test samples were implanted into the subcutaneous back of each nude mouse for 28 d so that the samples could be affected by the more active motion of the body and the results could be compared to those obtained on the abdominal side (Fig. S13). The gene expression of pro-adipogenic markers increased significantly from the gel and silicone to strut (−) and to strut (+) through strut-guided elastic holding, as determined by qRT-PCR analysis of C/EBPβ, C/EBPα, PPARγ, FABP4, and adiponectin (Fig. 4B). Furthermore, the most adipogenic morphology (lipid droplet area) was exhibited in strut (+), and prevalent tissue fibrosis was observed in silicone (Fig. 4C). In addition, the results were supported by the pro-adipogenic protein expression (PPARγ and FABP4) observed through immunofluorescence staining with confocal imaging (Fig. 4D). The formation of fibrotic tissue in silicone appeared to hinder adipogenesis.

Similar to back implantation, abdomen implantation was performed using rats for 42 d after partial defects of fat miniatures with hydrogel filing were made and covered without (gel) or with test membranes (silicone and strut +/−) (Fig. S13B). The overall trend in the gene expression of pro-adipogenic markers (C/EBPβ, PPARγ, and FABP4) was similar between the implants in the abdomen (Fig. S13C) and back (Fig. 4) according to qRT-PCR analyses. Furthermore, the differences between the test groups were not significant in some cases, including the expression of C/EBPβ and FABP4 between strut (+) and (−) as well as PPARγ expression between gel and silicone, indicating insufficient adipogenic stimulation by low motion frequency (Fig. S13A). These results were also evident in the comparison of the adipogenic lipid droplet area among the test groups (Fig. S13D and E). Highlighting the limitation of silicone implants, fibrotic tissue formation by silicone hinders adipogenesis, as seen in the back implants. The results confirm that high motion frequency, as seen in breast motion, is required to maximize the effect of elastic structural holding, justifying the use of strut (+) together with defect filing by decellularized matrix and autologous flap.

### Strut-guided preservation of pro-adipogenic mechanotransduction under motion

**Fig. 5.**
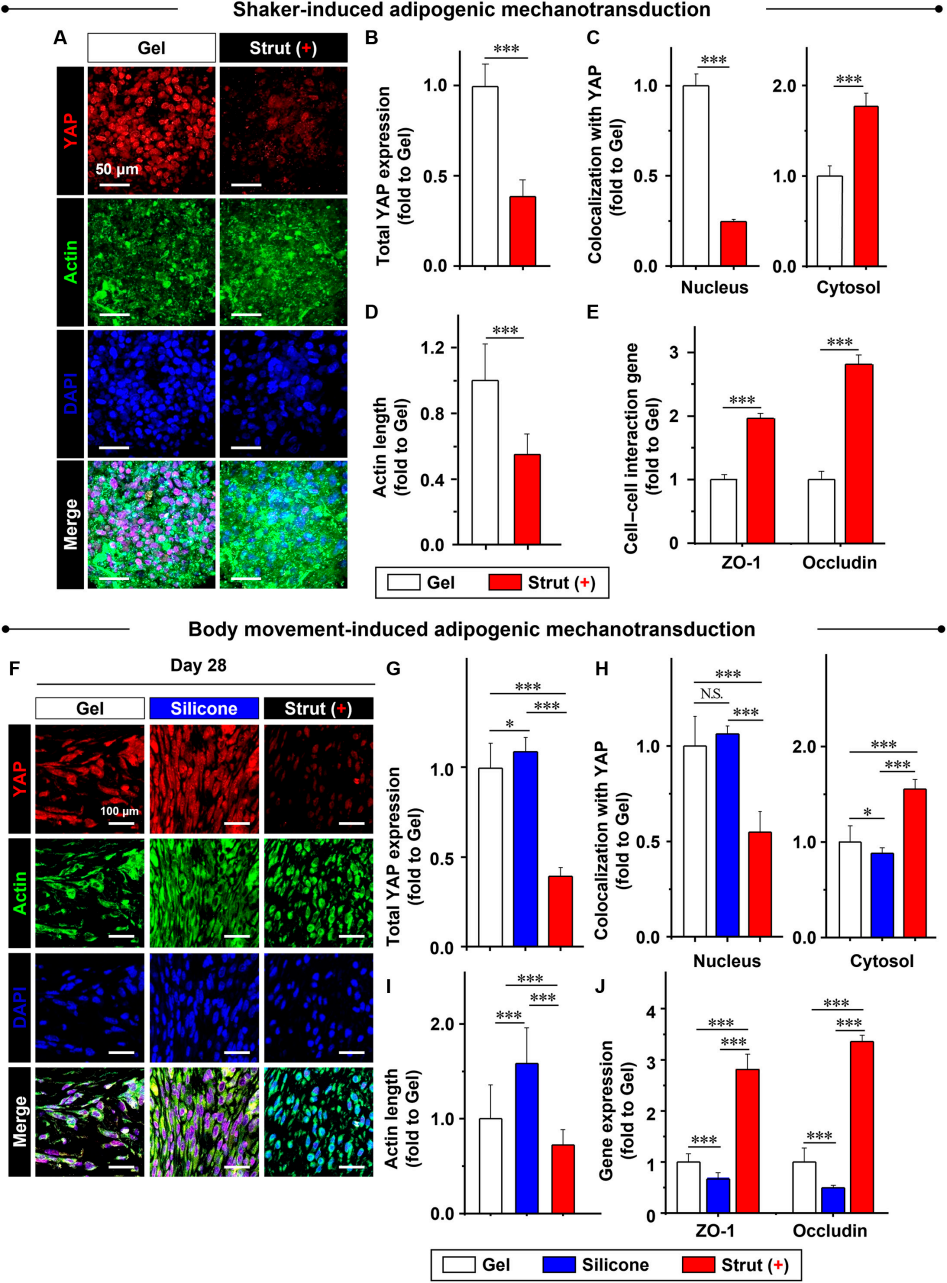
Strut-guided preservation of pro-adipogenic mechanotransduction under motion. For the following experiments, the fat miniature was partially defected and then filled with mPEG-PCL hydrogel. (A) When the fat miniatures without (gel) or with a membrane [strut (+)] covering were shaken on a rocker following treatment with adipogenic induction medium for 28 d, strut (+) significantly reduced (B) YAP expression, (C) nucleus colocalization with YAP as opposed to cytosol colocalization, and (D) actin length (E) while significantly promoting cell–cell interactions as evidenced by the expression of ZO-1 and occludin, compared to gel. (F) When the fat miniatures without (gel) or with silicone (commercial control) or strut (+) membrane covering were implanted into the subcutaneous back of nude mice for 28 d, strut (+) significantly reduced (G) YAP expression, (H) nucleus colocalization with YAP as opposed to the cytosol colocalization, and (I) actin length (J) while significantly promoting cell–cell interactions as evidenced by the expression of ZO-1 and occludin, compared to gel and silicone. The tissue fibrosis by silicones appears to disrupt the audiogenic mechanotransduction most. Data are mean ± SD. *P < 0.05, **P < 0.01, and ***P < 0.001 between the lined groups (*n* = 3 and 6 in the in vitro and in vivo experiments, respectively).

High motion frequency promotes the pro-adipogenic effects of strut (+) through elastic structural holding by suppressing large and heterogeneous deviations of breast motion. Hence, the activation of pro-adipogenic mechanisms needs to be examined to confirm the mechanism through in vitro and in vivo motion models. First, fat miniatures without (gel) or covered by a membrane [strut (+)] were shaken on a rocker under adipogenic induction medium for 28 d (Fig. 5A). As a series of indications for activation of pro-adipogenic mechanotransduction, strut (+) significantly reduced the YAP expression (Fig. 5B), nucleus colocalization with YAP as opposed to cytosolic colocalization (Fig. 5C), and actin length (Fig. 5D) while significantly promoting cell–cell interactions, as evidenced by the higher expression of ZO-1 and occludin, compared to Gel (Fig. 5E).

Fat miniatures without (gel) or with silicone (commercial control) or strut (+) membranes were implanted into the subcutaneous back of nude mice for 28 d (Fig. 5F). For these in vivo experiments, silicone was included as the test group because the effect of fibrotic formation by silicone became more apparent upon implantation. Strut (+) significantly reduced the YAP expression (Fig. 5G), nuclear colocalization with YAP as opposed to cytosolic colocalization (Fig. 5H), and actin length (Fig. 5I) while significantly promoting cell–cell interactions, as evidenced by the expression of ZO-1 and occludin, compared to gel and silicone (Fig. 5J). Tissue fibrosis induced by silicones appears to disrupt adipogenic mechanotransduction. These results indicate that strut (+) enables the conversion of motion control to cellular signaling through stable mechanotransduction, thereby promoting fat regeneration.

## Discussion

Structural integration and population gathering play a pivotal role in the propagation and movement of matter [38,39]. This phenomenon has been observed in fundamental cases, such as chemical bonding to direct material properties (ductility and hardness), human growth and movement against gravity, cancer growth in mass forms, and bacterial aggregation to build up biofilms [38,40–42]. This approach achieves its aims by holding the structure tightly so that it can thereby operate stably without disintegration and excessive deviation. This approach has been applied to (a) wound healing by tightening tissues but maintaining movement and (b) tissue-engineered constructs by physical scaffolding and synchronization with tissue movement. Although the concept is employed prevalently for medical treatments such as skin bandages, bone casts, joint protectors, suturing, and sealants, scientific insight has been insufficient, and the mechanistic understanding remains unexplored. Moreover, the material properties and structural design of medical devices are often considered separately [43]. Their cooperative functions are rarely strategized owing to a lack of full understanding of the mechanism of structural integration with movement synchronization upon implantation. In this regard, the current study is significant because insight into structural holding is provided with an understanding of the mechanotransduction mechanism by focusing on the cooperation between the material properties and structural design of strut (+).

Breast fat regeneration was chosen as the target of the approach using strut (+) because fat regeneration requires defect filing with autologous flat [44] or acellular dermal matrix [45,46] as well as shape holding by care bras or bandages, and the breast is exposed to heterogeneous directional pressure upon body movement [47]. Elastic structural holding can be achieved by combining the unique polymer properties and structural design of a membrane. The 6-arm 96% PCL-co-04% PGMA possesses a shape memory function due to the thermomechanical properties of elastomer. Hence, *T*_m_ and mechanical properties can be adjusted by altering the ratio of PCL to PGMA and the consequent crosslinking degree as reported in our previous studies [48–50]. These tunable properties provide several options to mold the polymer following the patient-specific breast shape. The polymer can be molded quickly for 5 s at 45 °C or slowly for 300 s at 40 °C, where the polymer chain mobility increases to reach the elastic switch as the heat energy is accumulated over time. Then, the shape can be fixed at room temperature (25 °C) through the release of heat energy. The molding capability serves as a foundation of structural holding, whereas the elastic property enables synchronization with fat movement. Furthermore, the thermo-responsive polymer is validated as biodegradable and biocompatible. The slow degradation with 35% weight loss for 1 year can support a depot function of sustained drug delivery for a long term. In addition, tissue regeneration can be supported under implantation in cooperation with the cytocompatible, antibacterial infection, nonirritable, and hemocompatible properties by relying on the basic nonimmunogenic, nonallergenic, and noncarcinogenic characteristics of PCL as approved by FDA previously [26,29–32].

However, the structural holding function cannot sufficiently suppress the heterogeneous and large deviation of breast fat movement in response to dynamic body motions. A key design concept of membrane was developed to handle the anisotropic property of breast tissue as this developmental feature should be inevitably produced through adaption with standing and lying under the gravity [16,24]. The anisotropic property was matched by adding a strut into each honeycomb in the direction perpendicular to gravity [15]. As a result, the stress distribution was efficiently handled to low levels with suppression of stress concentration over the entire membrane structure, and the resistance to deformations was improved upon standing or lying in simulation of the breast motion. A device’s structure can guide biological functions according to the structure–function relationship theory. Strut (+) promotes fat regeneration by stably activating pro-adipogenic mechanotransduction as a key mechanism, and this action requires a high motion frequency, as evidenced in the back and abdomen in rodent models. These results indicate that strut (+) mediates the translation of mechanical motion into beneficial cellular signaling. These results are significant in the field as they can inform the reformation of current designs for existing implantable devices. In particular, devices implanted into dynamic movement and load-bearing sites become more advantageous if they are subjected to this design reformation. Moreover, because each device design should be approached differently, the cross-validation approach among computational analysis, in vitro, and in vivo experiments can serve as an educational model for a broad audience.

The fat miniature and hydrogel filing for fat regeneration upon the partial defect represent another advanced aspect of the experimental model. First, the size and frequency of miniature and defect can be controlled in a user-specified manner. Second, the injectable mPEG-PCL was used to fill the defect so that the shape deformation could be prevented, and the hydrogel biodegradability left a room to induce the invasion of adipogenic cells and tissues. Third, the shaking model using a rocker and implantation into the motion-prone back and the abdomen, which has a low motion frequency, also provides significant insight into the pro-adipogenic mechanotransduction mechanism, thereby further highlighting the significance of the current study. As a mouse model, the fat miniature with breast cancer cells was considered to implant into the motion-prone back so that a surgical defect to remove the cancer could serve as a real-world model of breast reconstruction. However, the cancer cells exhibited accelerated degradation of gelatin gel upon motion stimulation, and thus, the structure of caner fat miniature could not be maintained stably enough to define the implantation position. The present model of fat miniature was used to validate the elastic structural holding of the body-shaping membrane by relying on the unique cooperation between structural design with material moldability. Hence, the cancer miniature is one of the important subjects to study in the next step so that the effect of elastic structural holding on fat regeneration with a risk of cancer recurrence can be examined after a right model of fat and cancer cells is developed.

Moreover, a future study requires a systemic approach to examine more potential designs by optimizing the direction and number of struts in addition to variation of the basic honeycomb structure to others. The present study served as a proof of concept by starting with the isotropic honeycomb structure, followed by addition of strut as an anisotropic factor to minimize the deformation by the body motion against the gravity. Computational calculation will be approached to select the most efficient design among the maximum variations of each structural factor, thereby advancing the design concept to the real-world success in the clinic. Other organs that are subjected to dynamic movements, such as muscle and skin, serve as future targets of the approach used in this study. As injectable mPEG-PCL is used to fill the partial defect, the use of a flat and acellular dermal matrix with strut (+) needs to be examined for further clinical translation. This study did not consider different sizes and shapes of the breast, so variation in patient-specific factors such as breast shape will be investigated in a future study. Nonetheless, the present study provides significant insight into a new means of accelerating breast fat regeneration, which is an underexplored area.

## Materials and Methods

### Cell and animal

The human pre-adipocyte and mouse fibroblast cell line (L929) were purchased from Lonza (Basel, Switzerland) and American Type Culture Collection (ATCC; VA, USA), respectively. Pre-adipocytes were cultured on tissue culture polystyrene (TCPS; SPL Life Science, Gyeonggi-do, Republic of Korea) dishes in Pre-adipocyte Basal Medium-2 (PBM-2, Lonza). L929 was cultured on TCPS in Dulbecco’s modified Eagle’s medium-low glucose (DMEM; Gibco, MA, USA) with supplementation of fetal bovine serum (10% v/v, Gibco) and penicillin–streptomycin (1% v/v, Gibco). When cells were propagated until they reached 80% confluence, they were passaged by detachment using 0.25% trypsin/ethylenediaminetetraacetic acid (Gibco).

Sprague-Dawley rats (8 weeks old and male) and BALB/c nude mice (6 weeks old and male) were purchased from Orient Inc. (Gyeonggi-do, Republic of Korea). The New Zealand white rabbits were purchased from DooYeol Biotech (Seoul, Republic of Korea). The Institutional Animal Care and Use Committee of the Yonsei Laboratory Animal Research Center (permit no. 2019-0205 and 2020-0071) and Korea Testing & Research Institute (permit no. IAC2022-1336) approved all animal protocols.

### Polymer synthesis

As a thermo-responsive elastomer, the membrane polymer (6-arm 96% PCL-*co*-04% PGMA) was synthesized by ring-opening polymerization of ε-caprolactone (CL; Sigma-Aldrich, MO, USA) and glycidyl methacrylate (GMA; Sigma-Aldrich). Briefly, dipentaerythritol (0.5 mmol, Sigma-Aldrich), hydroquinone (2.1 mmol, Sigma-Aldrich), and distilled CL (329 mmol) were prepared in a 3-necked flask. The mixture was stirred at 110 °C for 10 min. GMA (21 mmol) was added, and the reaction was allowed to proceed for 10 min. Then, a solution of 1,5,7-triazabicyclo [4.4.0] dec-5-ene (Sigma-Aldrich) and anhydrous acetonitrile (Sigma-Aldrich) (3.5 mmol) was added and reacted at 110 °C for 6 h under a nitrogen atmosphere. The product was dissolved in chloroform and precipitated using cold diethyl ether (Sigma-Aldrich). The final product, 6-arm 96% PCL-*co*-04% PGMA (%: molar ratio), was obtained after vacuum drying for 24 h.

### Polymer characterization

The chemical structure of 6-arm 96% PCL-*co*-04% PGMA with the molar ratio (%) was examined using ^1^H-NMR (400-MHz NMR, Avance III, Bruker Biospin, MD) spectra with chloroform-d (CDCl_3_, Sigma-Aldrich) solvent. The polymer was dissolved in *N*-methyl-2-pyrrolidone (NMP; Sigma-Aldrich, 1 g·ml^−1^) with 1% photo-initiator (Irgacure 2959, Sigma-Aldrich). This was followed by the casting of rectangular films (5 mm × 50 mm). A UV lamp (300 to 400 nm, UVACUBE 400; Hoenle, Germany) was used to crosslink the solution for 200s.

The thermomechanical properties of polymer were characterized by differential scattering calorimetry (DSC; DSC214, NETZSCH, SELB, Germany) and dynamic mechanical analysis (DMA; Discovery DMA850, TA Instruments, New Castle, DE, USA). First, DSC was used to determine melting temperature (*T*_m_), melting enthalpy (∆*H*_m_), crystallization temperature (*T*_c_), crystallization enthalpy (∆*H*_c_), and glass transition temperature (*T*_g_) at a heating rate of 10 °C·min^−1^ under a nitrogen atmosphere. Next, DMA was used to determine Young’s modulus in a strain-controlled mode with a rate of 5 mm·min^−1^ after incubation at each temperature (25, 37, 40, and 45 °C) for 5 min. The mechanical properties were characterized using a UTM (34SC-1, Instron, Norwood, MA, USA) at 37 °C. The samples were elongated at a strain rate of 10 mm·min^−1^ until the samples broke and then compared to silicone membranes from a Bellagel implant (Bellagel BRTZ-H 325, Hansbiomed Inc., Seoul, Korea).

### Polymer degradation

The polymer degradation was determined using the accelerated aging test, following the ASTM international standard 1980 [37]. Equations 1 to 3 were used to calculate the period of a real-world lifetime (RT) equivalent usage.

$$\mathrm{AAT}=\left[\mathrm{Desired}\;\left(\mathrm{RT}\right)\right]/\mathrm{AAF}$$ (1)

$$\mathrm{AAF}=Q_{10}\left[\left(\mathrm{TAA}-\mathrm{TRT}\right)/10\right]$$ (2)

$$Q_{10}=2.0$$ (3)

where AAT is the accelerated aging time, AAF is the accelerated aging factor, Desired (RT) is the desired period of real time, Q_10_ is the temperature coefficient (how material quickly changes upon increasing temperature by 10 °C), TAA is the accelerated aging temperature (70 °C), and TRT is 37 °C.

The condition of accelerated aging experiment was established by incubating test polymer films in saline (Dai Han Pharm. Co. Ltd., Seoul, Republic of Korea) at 70 °C for 37 d, followed by calculating the values corresponding to 1 year with determination of the weight loss (%) using Eqs. 4 to 6:

AAF=2.03.3=9.9 (4)

AAT=365/9.9=36.9 (5)

$$\left.\left[\left(W_{0}-W_{a}\right)/W_{0}\right]\right)\;\times \;100\%$$ (6)

where *W*_0_ is the initial weight and *W*_a_ is the weight after degradation.

### Polymer biocompatibility

Biocompatibility tests [(a) cytotoxicity, (b) bacteria adhesion test, (c) intracutaneous reactivity, and (d) blood compatibility] were carried out according to the principles of GLP (ISO 10993-10).

First, the cytotoxicity was tested using elution of polymer films in DMEM for 72 h (1 g·5 ml^−1^), which was then added to L929 cells (5 × 10^4^ cells/well) in a series of dilutions (50%, 75%, and 100%) for 72 h. Cell viability was determined using Cell Counting Kit-8 (CCK-8; Dojindo, Rockville, MD, USA) with absorbance readings at 450 nm in a microplate reader (BioTek, Winooski, VT, USA).

Second, bacterial adhesion test was performed as an indication of antibiofilm formation in comparison with silicone [35]. Briefly, *P. aeruginosa* (ATCC; Korean Culture Center of Microorganisms, Seoul, Korea) was cultured in a tryptic soy agar (Sigma-Aldrich) at 37 °C until they grew up to the mid-log phase as indicated by the optical density of 0.55 in microplate spectrophotometric reading at 600 nm (Infinite M Nano, Tecan, Männedorf, Switzerland). Then, bacteria were incubated to adhere to rectangular polymer films (1.5 × 1.5 cm) for 24 h at 37 °C. Bacterial adhesion was visualized by field-emission SEM (FE-SEM; MERLIN, Zeiss, Oberkochen, Baden-Wüttemberg, Germany) with quantitative image analysis of the number of adhered bacteria using ImageJ software [National Institutes of Health (NIH), MD, USA].

Third, the intracutaneous reactivity was examined using an extract of polymer films in saline or cottonseed oil (Junsei Chemical Co. Ltd., Tokyo, Japan) for 72 h (1 g·5 ml^−1^). Each test sample (200 μl) of sterile saline and cottonseed oil with or without extract was intracutaneously injected into each of 5 sites in the cranial end of rabbits (*n* = 3; Fig. S4), followed by examining the local response at intervals (immediately, 24 h, 48 h, and 72 h) after injection. The local reaction was graded for erythema and edema of each injection site using the standard scoring method (Table S4).

Finally, blood compatibility was tested by collecting blood from the ear artery of rabbits in vacuum tubes containing anticoagulants (Sigma-Aldrich) (*n* = 3). After mixing the blood (20 μl) with Drabkin’s solution (5 ml, Sigma-Aldrich) for 15 min, the absorbance was measured in a microplate reader at 540 nm. The hemoglobin amount in the blood was determined using Eq. 7:

$$\mathrm{TBH}=A^{\mathrm{TBH}}\times F\times \mathrm{dilution factor}$$ (7)

where TBH is the accelerated aging factor, *A*^TBH^ is the absorbance of total blood hemoglobin concentration, and *F* is the slope.

The thermo-responsive polymer, polyethylene (Sigma-Aldrich, negative control), and plasticized polyvinyl chloride (Sigma-Aldrich, positive control) were prepared to either 1.4 g or 4.2 cm^2^ with 0.91% nonionic surfactant (Hatano Research Institute, Kanagawa, Japan) in phosphate-buffered saline (PBS) (7 ml). Each test sample (1 ml) was reacted with hemoglobin (10 mg/ml) at 37 °C for 3 h and then mixed with 1 ml of Drabkin’s reagent for 15 min, followed by absorbance reading at 540 nm using a microplate reader. The hemolysis (%) was calculated using Eq. 8.

$$\mathrm{Hemolysis}\;\left(\%\right)=\frac{A^{S}-A^{B}}{A^{T}-A^{B}}\times 100$$ (8)

where *A*^S^ is the absorbance of test samples, *A*^B^ is the absorbance of solvent control, and *A*^T^ is the absorbance of hemoglobin concentration.

Consequently, the hemolytic index was calculated to determine the hemolytic grade using Eq. 9 with the standard scoring (Table S7).

$$\begin{array}{c} \begin{array}{c} \mathrm{Hemolytic index}=\%\;\mathrm{hemolysis of polymer or positive control}\\ -\%\;\mathrm{hemolysis of negative control} \end{array} \end{array}$$ (9)

### Computational modeling analysis

Flat (2D) and hemispherical (3D) membrane models were produced using CAD (Fusion 360, version 2020, Autodesk, CA, USA). The 2D flat membranes were designed to a panel of square, honeycomb [strut (−)], and addition of strut into the middle of the honeycomb in the direction perpendicular to gravity [strut (+)]. Structural modeling was performed using ANSYS 2020R1 software (ANSYS, Canonsburg, PA, USA) based on the input of the membrane mechanical properties as experimentally obtained by UTM. Membrane geometries were constructed by discretizing the mesh structure using the FEM.

The membrane structural effects were analyzed by computational modeling with the assumption that 3 N is loaded onto a breast as an average pressure (tension and compression) from a lying or standing position in daily life [48]. Subsequently, when the 2D and 3D membranes were elongated and compressed, respectively, the stress, deformation degree, and circularity of the geometries were analyzed using ImageJ (NIH).

### Membrane production

CAD designs were used to produce flat (2D) membranes by 3D printing (IMC, Carima, Seoul, Republic of Korea), and the structures were used to generate reverse polydimethylsiloxane (PDMS; Dow Corning, Midland, MI, USA) molds. Two holes were punched in each PDMS mold using a 1-mm biopsy punch (KAI Medical, Gifu Prefecture, Japan), and these holes were used as the inlet and outlet for solution injection. The PDMS mold was then surface-modified to attach it to a glass plate.

The membrane polymer was dissolved in NMP (1 g·ml^−1^, Sigma-Aldrich) with a 1% photoinitiator (Irgacure 2959, Sigma-Aldrich). Then, the solution was injected through the inlet. After undergoing crosslinking for 200 s under a UV lamp, the membranes were obtained and washed in distilled water (D.W.) for 2 d. The membranes were vacuum-dried for 24 h and stored at room temperature until further use. The membrane structures were confirmed by FE-SEM and optical microscopy (Leica DMi8; Leica Microsystems, Wetzlar, Germany).

### Membrane characterization

The moldability was determined by soaking the 2D type membranes at 45 °C (above *T*_m_) for 5 s and at 40 °C (below *T*_m_) for 300 s so that the crosslinked polymers became flexible enough to undergo structural changes. The membranes were then molded on a 3D hemispherical mold that was generated by 3D printing based on CAD (Fusion 360 program), followed by fixing the hemispheric shape at room temperature by releasing heat energy. The fixity of the hemispheric membrane shape was determined by examining the shape maintenance in 37 °C water using optical microscopic imaging (Leica DMi8). The effects of strut (+/−) on elastic structural holding were examined by shaking a breast gel model on a rocker. The gel model was produced by embedding a gelatin/microbial transglutaminase (mTG) solution (9:1 ratio, final concentration = 5%, w/v; hydrogel) in a hemispheric PDMS mold (reverse) from 3D printing. Hemispherical gels with or without membranes were shaken on a rocker and visualized by digital imaging to determine the movement deviation from the original center position. The mechanical properties of the flat (2D) membranes (strut +/−) were characterized using UTM to determine the stress–strain behavior upon elongation at 10 mm·min^−1^ (*n* = 7). The force–strain curve was determined using hemispherical (3D) membranes (strut +/−) upon compression at 1 N·min^−1^ (*n* = 7).

### Hydrogel synthesis

An mPEG-PCL di-block copolymer was synthesized by ring-opening polymerization of CL (Sigma-Aldrich) and poly (ethylene glycol) methyl ether (mPEG; *M*_n_ = 750, Sigma-Aldrich) in the presence of tin (II) 2-ethylhexanoate [Sn (Oct)_2_, Sigma-Aldrich] as the catalyst [49]. Briefly, mPEG (10 mmol) and distilled CL (150 mmol) were prepared in a 3-necked round-bottom flask under a nitrogen atmosphere. The mixture was heated up to 140 °C and then allowed to react with Sn(Oct)_2_ (0.2 mmol) for 1.5 h. The product was dissolved in dichloromethane and precipitated with cold diethyl ether, followed by vacuum drying for 24 h to obtain mPEG-PCL.

### Hydrogel characterization

The structure and molar ratio (%) of mPEG-PCL were determined by ^1^H-NMR spectroscopy using chloroform-d as the solvent. The thermal properties of mPEG-PCL were characterized using DSC at a heating rate of 10 °C·min^−1^ under a nitrogen atmosphere. The sol–gel transition of mPEG-PCL was determined by inverting vials containing mPEG-PCL by varying the concentration (5%, 10%, 15%, and 20%, w/v, in 1 ml of PBS). The solutions were heated at 80 °C for 15 min, cooled down at 4 °C for 24 h, and then immersed in a water bath by increasing the temperature from 25 to 60 °C by 2.5 °C increments. The vials were stored at each temperature for 10 min and inverted to check for gelation [50]. The cytotoxicity of mPEG-PCL was determined using a CCK-8 assay (Dojindo). Briefly, mPEG-PCL (20%, w/v) was solubilized in PBS, which was then placed in a 48-well plate (SPL), and incubated for gelation at 37 °C. L929 cells (1 × 10^5^ cells/well) were seeded on the gel for 1 d and then treated with CCK-8 solution for 4 h at 37 °C. The solution was transferred to a 96-well plate, and the absorbance was read at 450 nm in a microplate reader.

### Production of fat miniature

As a miniature breast fat structure, a reverse PDMS mold with a hemispherical shape was produced using CAD and 3D printing, as described previously. To produce the fat miniature, (a) AggreWell 800 24-well culture plates (STEMCELL Technologies, Vancouver BC, Canada) were used after rinsing with an antiadherent solution (STEMCELL Technologies) for 24 h; (b) as a cell component of the fat miniature, pre-adipocyte spheroids were then produced by culturing on AggreWell plates (passages 4 to 6, 2.4 × 10^6^ cells/well); (c) the spheroids (4.8 × 10^6^ cells) were mixed with a gelatin/mTG solution (9:1 ratio, final concentration = 5%, w/v) and placed in a PDMS mold, followed by gelation as a hemispheric structure [24]; and (d) fat miniatures were cultured using Pre-adipocyte Growth Medium-2 Bullet Kit (PGM-2; Lonza) medium for 2 weeks.

Next, pre-adipocytes (1.2 × 10^6^ cells/well) were seeded in 6-well plates as a 2D culture. The cells (4.8 × 10^6^ cells/ml) were mixed with a gelatin/mTG solution (9:1 ratio, final concentration = 5%, w/v) for 3D suspension culture. After culturing 2D, 3D suspension, and fat miniature using PGM-2 for 2 weeks, the gene expression of adipose-specific factors (PPARγ and adiponectin) was determined using qRT-PCR [60]. Adipogenic phenotypes were examined by AdipoRed staining (Lonza) following the manufacturer’s protocol. Cell nuclei were counterstained with Hoechst 33342 (1:200; Thermo Fisher), followed by confocal imaging (LSM980; Zeiss, Oberkochen, Land Baden-Württemberg, Germany).

### In vitro study with shaking fat miniature

As a model for breast fat regeneration, the miniatures were partially defected using a 3-mm biopsy punch (KAI Medical) that was filled with mPEG-PCL hydrogel [20% (w/v) in PBS]. Subsequently, the test membranes were covered with molding using a silicone ring.

The pro-adipogenic effect of strut (+/−) was determined by culturing fat miniatures in static versus shaking conditions on a rocker for 1, 2, and 4 weeks using a PGM-2 differentiation medium and then comparing the results obtained under these conditions with those obtained under conditions with no membrane (Gel). The gene and protein expression of adipogenic markers (C/EBPβ, C/EBPα, PPARγ, FABP4, and/or adiponectin) was determined using qRT-PCR, Western blotting, and immunocytochemistry with quantitative analyses using ImageJ. Subsequently, the effects of elastic structural holding by strut (+) on pro-adipogenic mechanotransduction were determined and compared with those of the gel after 14-d culture. As the activation of pro-adipogenic mechanotransduction results in actin contraction [16,20], actin length was analyzed by fluorescein isothiocyanate–phalloidin (Thermo Fisher Scientific, MA, USA) staining, followed by quantitative analysis using ImageJ. The expression of cell–cell adhesion marker genes (ZO-1 and occludin) was determined using qRT-PCR. As pro-adipogenic mechanotransduction is activated, the overall expression of YAP decreases, and YAP is relocated from the nucleus to the cytosol [15,18]. The protein expression and intracellular location of YAP were analyzed quantitatively by immunocytochemistry (*n* = 3 for all experiments).

### Animal studies with low versus high motion frequency site

Because the back moves more frequently than the abdomen in rodents, pedometers (MK-365LS, YAMASA, Japan) were mounted on the abdomen of Sprague-Dawley rats and the back of BALB/c nude mice to confirm the implantation sites of test groups with low and high motion frequency, respectively, for 42 d (*n* = 3). Fat miniatures (4.8 × 10^6^ cells) were produced with partial defects and subsequent hydrogel filing as described above. As the site with a high motion frequency, the subcutaneous layer of the mouse back (BALB/c nude, 6 weeks old, male, *n* = 6) was exposed, and fat miniatures with no (Gel), silicone, and strut (+/−) membranes were sterilized using EO gas and implanted under the back skin, followed by suturing the incision using 6-0 silk (AILEE Co., Busan, Korea). At 28 d after fat miniature transplantation, the mice were sacrificed using CO_2_ gas. Inflammatory responses in the test groups were examined by hematoxylin and eosin (H&E) staining and immunohistochemistry of the inflammatory marker (TNF-α). Bacterial infection of the implanted samples was determined by staining with gram’s crystal violet solution (0.1%, Sigma-Aldrich) for 15 min, followed by imaging using optical microscopy. For quantitative analysis, the dye was extracted from the bacteria using 95% (w/v) ethanol (Sigma-Aldrich), and the absorbance was measured at 600 nm using a microplate spectrophotometer.

To produce a model site with low motion frequency, Sprague-Dawley rats (*n* = 4, 8 weeks old, male) were fed a high-fat diet (D12079B; Research Diets, NJ, USA) to increase belly fat for 6 weeks. The subcutaneous adipose tissue of the abdomen was tied using a 4-0 silk (AILEE Co., Busan, Korea) to produce a breast fat-like shape. A partial defect was then made using a 3-mm biopsy punch with injection of 20% (w/v) mPEG-PCL gel and covered with or without the test membranes upon EO gas sterilization. After 28 and 42 d, the rats were sacrificed using CO_2_ gas for further analysis. The adipoconductive effect and mechanotransduction of the test groups were examined by determining the marker gene and protein expression as previously described.

### Quantitative RT-PCR

Total RNA was extracted from cells and tissues using TRIzol reagent (Thermo Fisher Scientific), according to the manufacturer’s protocol. The NanoDrop 2000 Spectrophotometer (Thermo Fisher) was used to quantify the RNA concentration by measuring *A*_260_. Complementary DNA (cDNA) was synthesized using AccuPower CycleScript RT Premix (Bioneer, Daejeon, Republic of Korea) and the T-100 Thermal Cycler (Bio-Rad, CA, USA) following the manufacturer’s protocol. qRT-PCR was performed using the StepOne Plus RT PCR System (Applied Biosystems, MA, USA) using a primer (Table S10), cDNA, and SYBR Green PCR mix (Applied Biosystems), followed by melting curve analysis. The result for each target gene was normalized to that of glyceraldehyde-3-phosphate dehydrogenase (GAPDH) and calculated using the comparative Ct (2^−∆∆Ct^) values.

### Western blotting

Total protein was extracted using radioimmunoprecipitation assay buffer (Sigma-Aldrich) with the 1% Halt Protease and Phosphatase inhibitor cocktail (Thermo Fisher). The Bradford assay (Sigma-Aldrich) was used to quantify the protein concentration. Proteins (30 μg) were separated on a 10 % SDS-polyacrylamide Mini-Protein TGX gel (Bio-Rad) and electrotransferred onto a nitrocellulose membrane using iBlot 2 NC gel regular stacks (Thermo Fisher), followed by blocking in 1× tris-buffered saline buffer (TBST; T&L, Seoul, Republic of Korea) with 5% skim milk (Bio-Rad) for 1 h at room temperature. The membranes were incubated with β-actin (1:2,000; Santa Cruz Biotechnology, CA, USA), C/EBPβ (1:1,000; Abcam, Cambridge, UK), C/EBPα (1:1,000; Cell Signaling Technology, Danvers, MA, USA), PPARγ (1:1,000; Santa Cruz Biotechnology), and/or FABP4 (1:1,000; Abcam, Cambridge, UK) after diluting in 5% skim milk TBST at 4 °C overnight. After washing 3 times (each 10 min) with 1× TBST, the membranes were incubated with secondary goat anti-rabbit IgG(H+L)-HRP-conjugated (1:5,000; Thermo Fisher) or goat anti-mouse IgG(H+L)-HRP-conjugated (1:5,000; Santa Cruz Biotechnology) antibodies for 1 h. The Western ECL substrate (Bio-Rad) was used to visualize the blot signals with analysis using LAS-3000 (Fuji Film, Tokyo, Japan), followed by quantitative analysis with normalization to β-actin intensity using ImageJ (NIH).

### Immunocytochemistry

The fat miniatures were harvested, rinsed using PBS 3 times, and fixed with 4% paraformaldehyde (CellNest, Gyeonggi, Republic of Korea) for 30 min at room temperature. The samples were permeabilized with 0.3% Tween 20 (Sigma-Aldrich) in PBS for 1 h at room temperature and blocked with 0.3% Tween 20 and 3% bovine serum albumin (BSA; Millipore, MA, USA) in PBS for 1 h at room temperature. Next, primary antibodies were treated on samples for analysis against PPARγ (1:200; Santa Cruz Biotechnology), FABP4 (1:200; Abcam), YAP (1:200, Cell Signaling Technology), and actin (1:200, Abcam) at 4 °C overnight. The samples were then washed with PBS and treated with secondary antibodies [anti-mouse Alexa Fluor 488 and anti-mouse Alexa Fluor 594 (The Jackson Laboratory, Bar Harbor, ME, USA)] in PBS for 2 h at room temperature. Cell nuclei were counterstained with Hoechst 33342 (1:200; Thermo Fisher). Confocal imaging (LSM980; Zeiss, Oberkochen, Land Baden-Württemberg, Germany) and quantitative image analysis (NIH) were then performed.

### Histological analysis

Tissues were harvested, fixed in 10% paraformaldehyde (Biosesang, Gyeonggi, Republic of Korea) for 1 d, and embedded in paraffin wax. Paraffin blocks were sectioned into 4-μm-thick slices using a microtome (Leica Microsystems, Deerfield, IL, USA), followed by deparaffinization, hydration, and H&E staining. The sections were treated with primary antibodies against PPARγ (1:200), FABP4 (1:200), YAP (1:200), actin (1:200), and TNF-α (1:200; Abcam) with dilution in PBS containing 0.3% Tween 20 and 3% BSA at 4 °C overnight. Then, the samples were washed using PBS 3 times (each 10 min) and incubated with secondary antibodies [anti-mouse Alexa Fluor 488 or 594 and anti-mouse Alexa Fluor 488 or 594 (The Jackson Laboratory)] for 2 h at room temperature. Cell nuclei were counterstained with Hoechst 33342 (1:200; Thermo Fisher), followed by confocal imaging (LSM980; Zeiss, Oberkochen, Land Baden-Württemberg, Germany) and quantitative image analysis (NIH).

### Statistical analysis

Statistical analysis was conducted using one-way analysis of variance (ANOVA) with post hoc Bonferroni’s and Tukey’s analyses using SigmaPlot (version 12.0, Systat Software, CA, USA). All data are presented as mean ± SD. The *P* values and sizes of the samples per group are denoted in each figure legend.

## Data Availability

All data needed to evaluate the conclusions in the paper are present in the paper and/or the Supplementary Materials.
