# Supplementary material for: Body-Shaping Membrane to Regenerate Breast Fat by Elastic Structural Holding
Source: Research (Wash D C). 2023 May 10;6:0137. doi: 10.34133/research.0137 (PMC10204741; doi:10.34133/research.0137)

Body shaping membrane to regenerate breast fat by elastic structural holding

**Hye-Seon Kim^1,§^, Jeongeun Park^1,§^, Hyun-Su Ha^1^, Sewoom Baek^1^, Chan Hee Lee^1^, Kyubae Lee^1^, Suji Park^1^, Jueun Kim^1^, Se Won Yi^1,^*, and Hak-Joon Sung^1,^***

^§, *^ Equal contribution

H.-S. Kim, J. Park, H. Ha, S. Baek, C.H. Lee, K. Lee, S. Park, S.W. Yi, and Prof. H.-J. Sung

Department of Medical Engineering, Graduate School of Medical Science, Brain Korea 21 Project, Yonsei University College of Medicine, Seoul 03722, Republic of Korea

E-mail: hj72sung@yuhs.ac

**
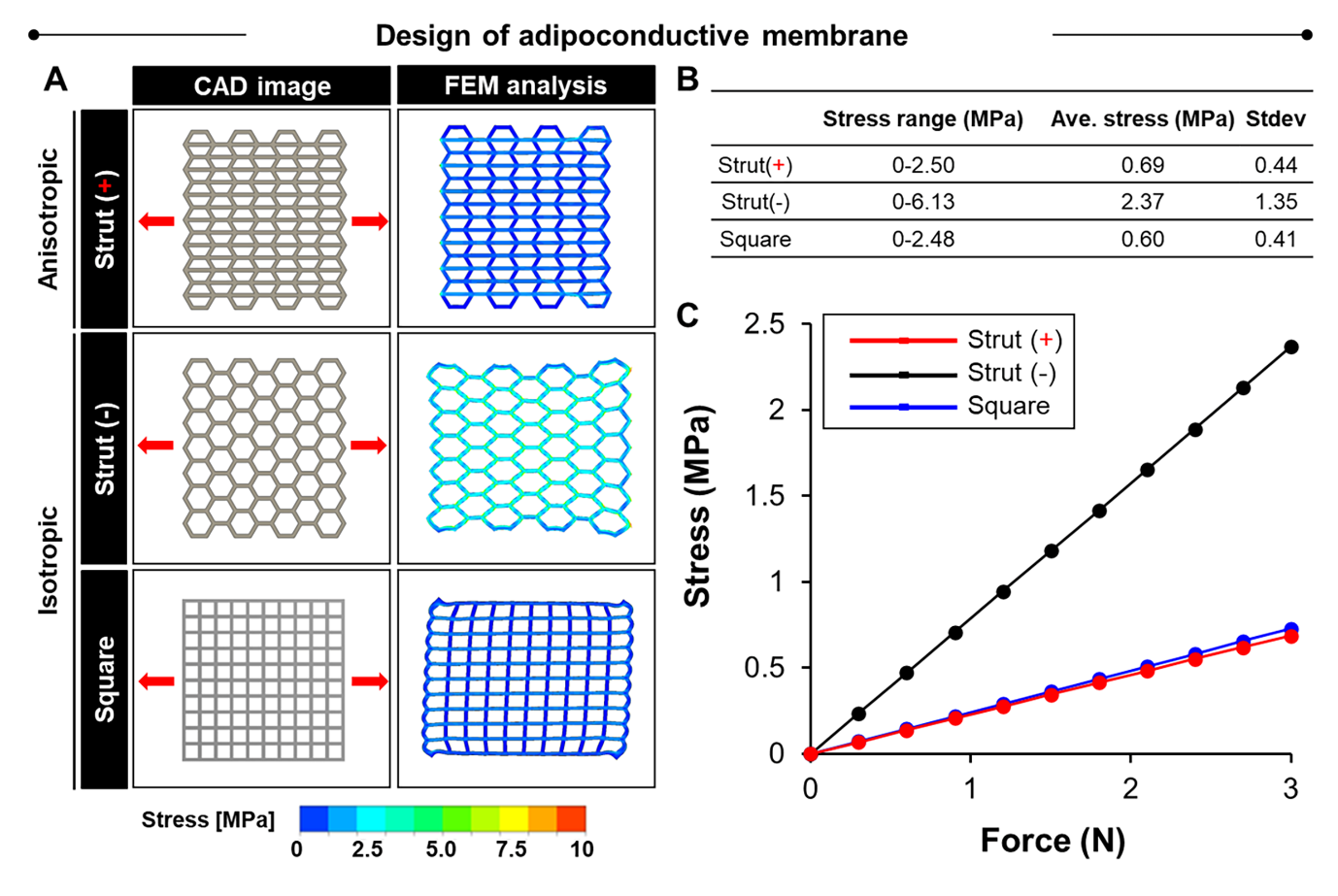
**


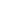


**Figure S1. Design justification of adipoconductive membrane.** The adipoconductive membrane was designed by adding struts [strut (+)] into the honeycomb structure [strut (-)]. **(A)** The test designs (CAD) and finite element method (FEM) analysis were set by strut (+), strut (-), and square as a form of 2D flat membrane. In response to the stretching deformation (red arrows), only strut (+) maintained the structural stability among the candidates. **(B)** Strut (+) and square managed the stress distribution effectively in the lower range [strut (+): avg. 0.69 in 0–2.5 MPa and square: ave.0.60 in 0–2.48 MPa] over the entire membrane compared to strut (-) (avg. 2.37 in 0–6.13 MPa) This result was supported **(C)** as the strut (+) and square maintains lower levels of stress than those of strut (-) in response to the same incremental force (0–3 N).

**
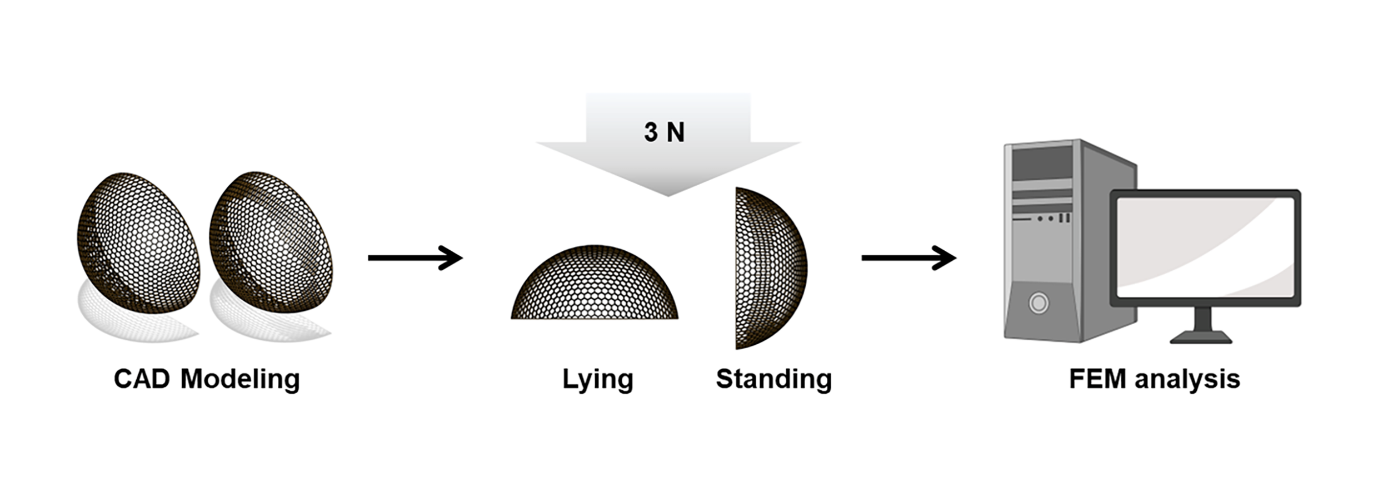
Fig. S2. Steps followed to analyze hemispherical membranes by FEM.** The hemispherical membranes (strut +/-) were modeled using CAD and subjected to 3 N in a lying or standing position, followed by FEM analysis of stress management with consequent structural deformation.

**
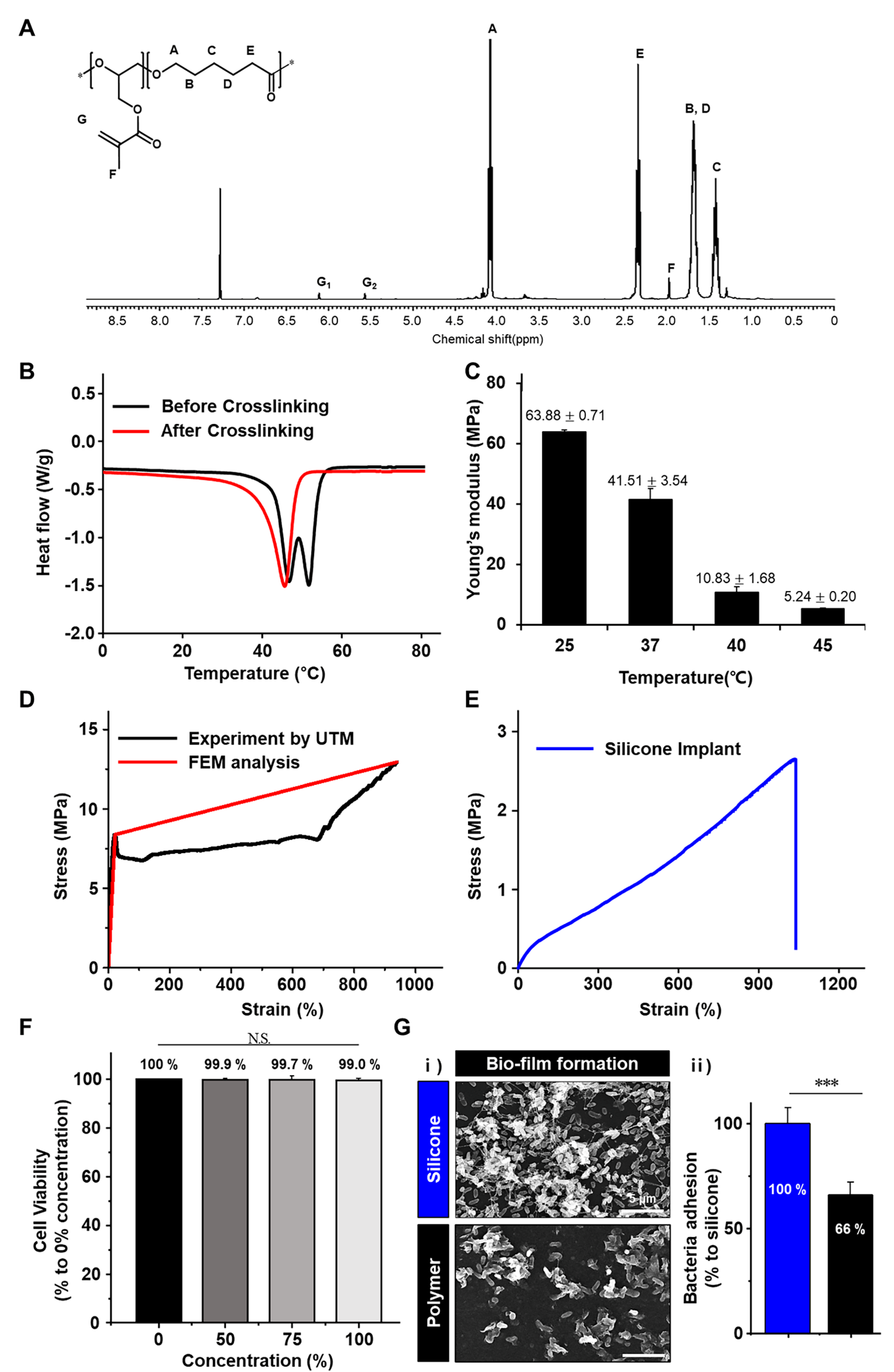
Fig. S3. Synthesis and characterizations of the membrane polymer (6arm_96% PCL-co_04%PGMA). (A)** The synthesis of the polymer was verified by analyzing the chemical structure as the peaks of proton nuclear magnetic resonance (^1^H-NMR) spectrometry indicate chloroform-d_6_ (CDCl_3_): δ (ppm) 6.13 (s, =CH_2_, G1), 5.58 (s, =CH_2_, G2), 1.97 (s, –CH_2_, F), 4.10 (m, –OCH_2_, A), 2.41 (m, –CH_2_, E), 1.74 (m, –CH_2_, D), and 1.45 (m, –CH_2_, B and D). The crosslinking of the polymer was verified by the **(B)** reduction of T_m_ upon crosslinking in the analysis of differential scanning calorimetry (DSC). **(C)** Young's modulus was determined by DMA after incubating the polymer at each temperature (25, 37, 40, and 45 °C) for 5 min. Hence, the transition temperature (T_t_) was determined to 45°C (T_m_ post crosslinking) due to the moldable flexibility of polymer chains. When the mechanical property of polymer film was analyzed using a universal testing machine (UTM), **(D)** the maximum tensile strength in the stress–strain curve aligned with the results of FEM analysis, and **(E)** compared to silicone, more stress is required to reach an over 900% strain, indicating the membrane’s potential to enable elastic structural holding. **(F)** After eluting the polymer (1 g·5 mL^-1^) in culture media at 37 °C for 72 h, the cytocompatibility of polymer was verified by varying the elution concentration using a CCK-8 assay to determine the cytotoxicity (% to that of 0% elute concentration). **(G)** After incubating *Pseudomonas* (P*.*) *aeruginosa* on test films at 37 °C for 24 h, the superior suppression of bacterial adhesion by the polymer (black) as an indication of anti-biofilm formation over silicone (blue) was determined by i) SEM with ii) quantitative analysis of the bacteria number. Data = mean +/- standard deviation. *p < 0.05, **p < 0.01, and ***p < 0.001 between the lined groups (n ≥ 3 in all analyses). N.S.= not significant.


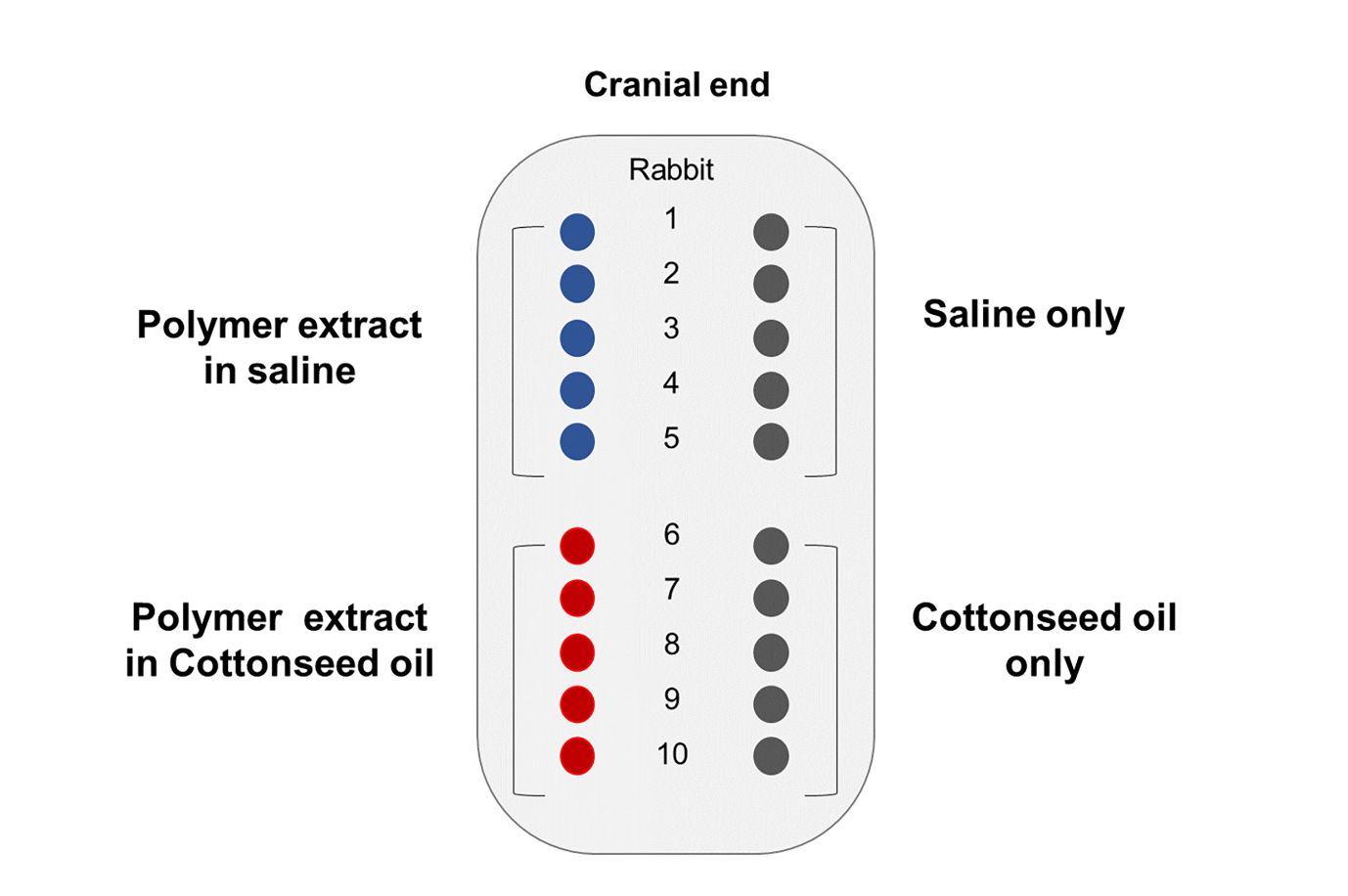


**Fig. S4. Injection sites of intracutaneous reactivity test in the cranial end of rabbit.** The polymer films were extracted to saline and cottonseed oil. Each of the extracts in either saline or cottonseed oil, saline only, and cottonseed oil only was intracutaneously injected into each of five sites in the cranial end of rabbit as shown above (n=3), followed by examining the local response at intervals (immediately, 24, 48 and 72 h) post-injection.

**
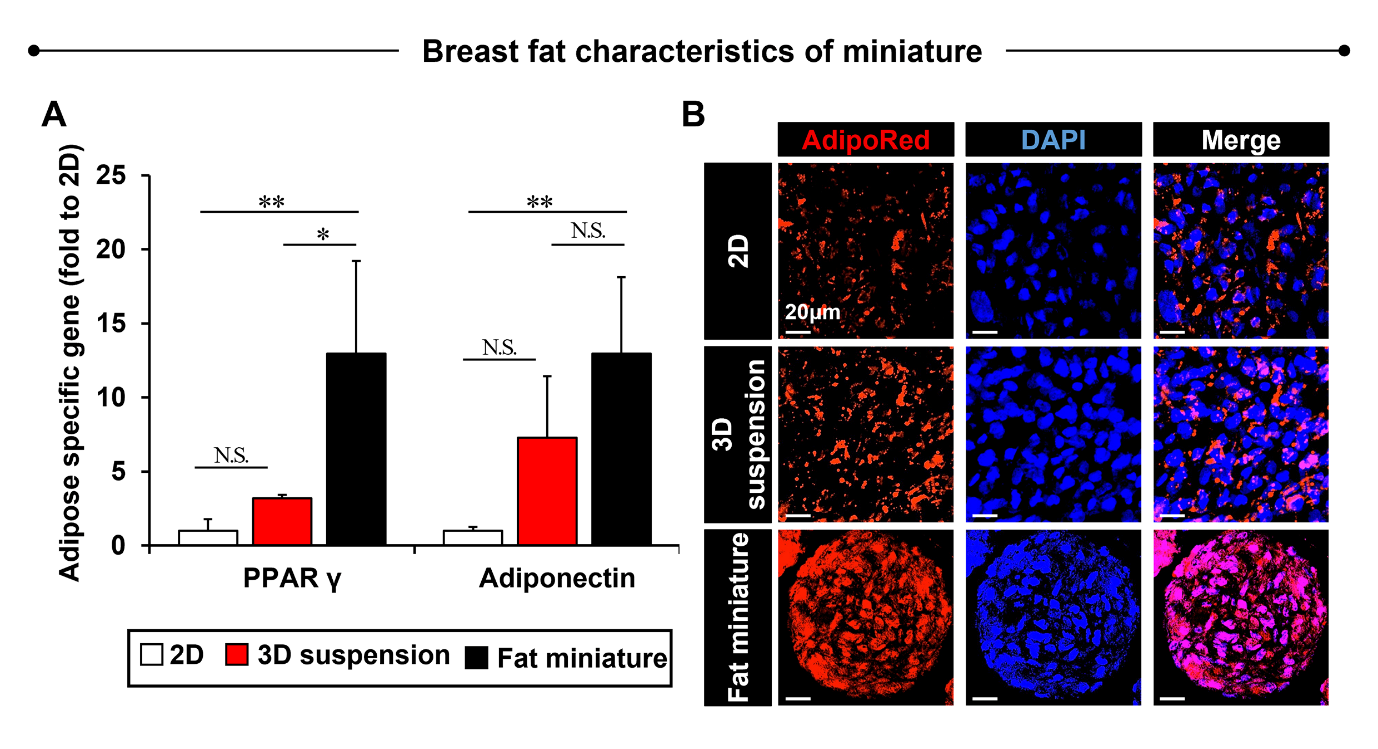
**

**Fig. S5. Characterization of fat miniature as a model of artificial breast fat.** **(A)** The fat miniature promoted the gene expression of adipose specific factors (PPARγ and Adiponectin) significantly compared to the 2D and/or 3D suspension culture after treatment of adipogenic induction media for 14 days upon qRT-PCR analysis. **(B)** The results were confirmed by the adipogenic features of fat miniature from AipoRed staining. Data = mean +/- standard deviation. *p < 0.05, **p < 0.01, and ***p < 0.001 between the lined groups (n = 3). N.S.=not significant.

**
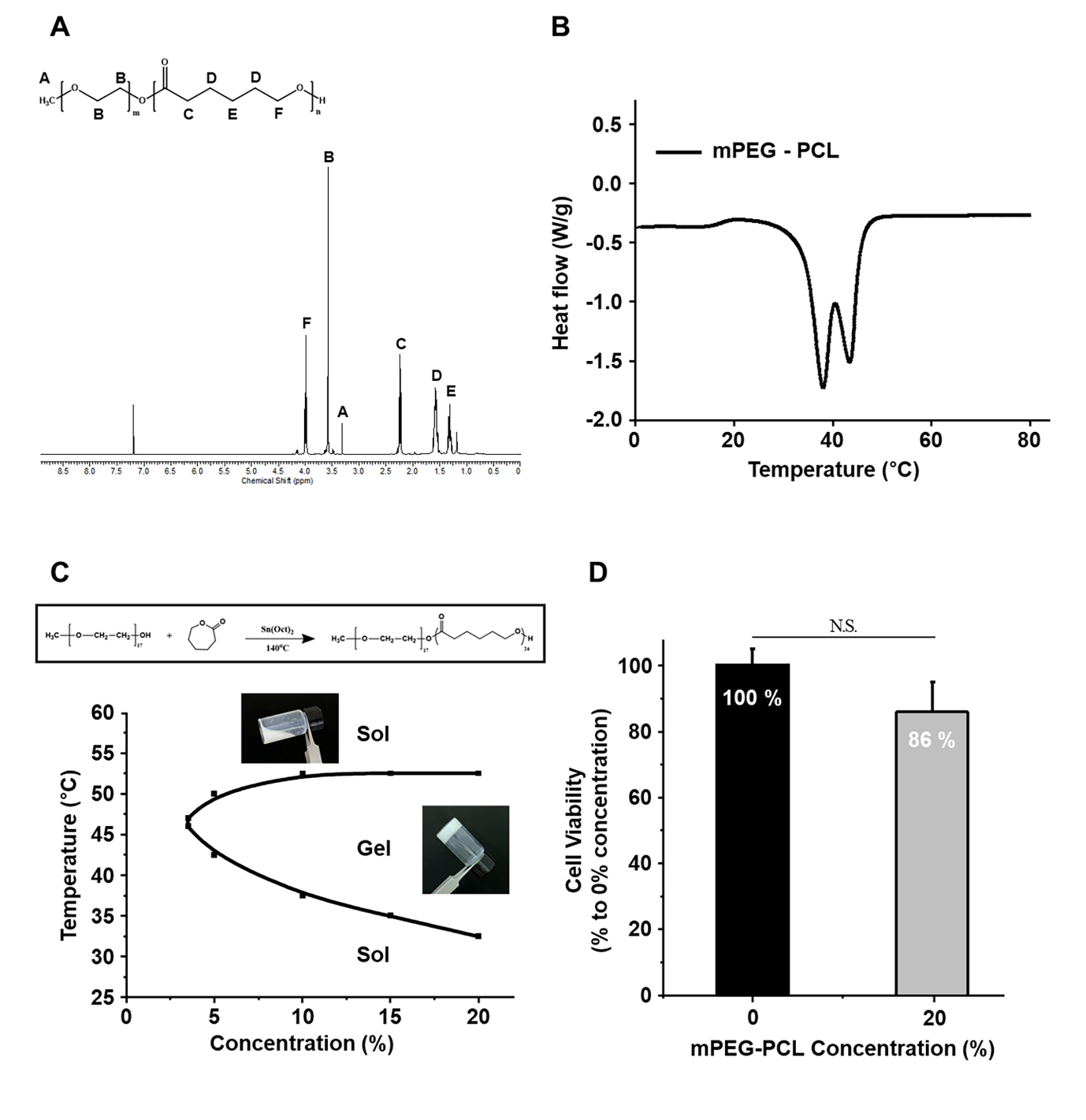
**

**Fig. S6. Synthesis and characterization of the mPEG-PCL hydrogel. (A**) The synthesis of mPEG-PCL hydrogel was confirmed by an ^1^H-NMR spectrometer with chloroform-d_6_ (CDCl_3_): δ (ppm) 4.01 (m, –OCH_2_, F), 3.67 (s, –OCH_2_, B), 3.35 (s, –CH_3_, A), 2.26 (m, –CH_2_, C), 1.62 (m, –CH_2_, D) and 1.43 (m, –CH_2_, E). **(B)** The T_m_ of the hydrogel appears around 40° C as a typical property of mPEG-PCL in the DSC analysis. **(C)** The phase diagram of hydrogel was created by analyzing the sol-gel phase transition in the test concentrations (w/v). **(D)** The cytocompatibility of mPEG-PCL hydrogel was verified by comparing the compatible cytotoxicity of L929 cells to that of no elute (0%) using a CCK-8 assay. Data = mean +/- standard deviation. *p < 0.05, **p < 0.01, and ***p < 0.001 between the lined groups (n = 3). N.S. = not significant.

**
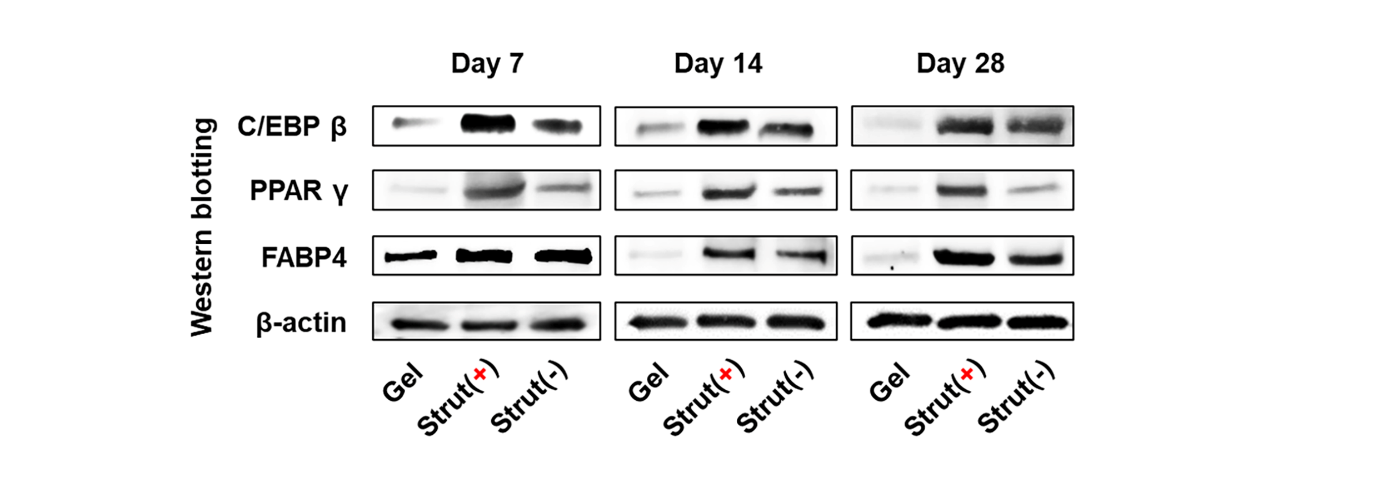
Fig. S7. Pro-adipogenic effect of Strut (+) on fat miniatures under shaking conditions.** Strut (+) promotes protein expression of adipogenic markers (C/EBPβ, PPARγ and FABP4) compared to Gel and strut (-) when western blotting is carried out on fat miniatures post-shaking following treatment with adipogenic induction media for 28 days (n = 3).

**
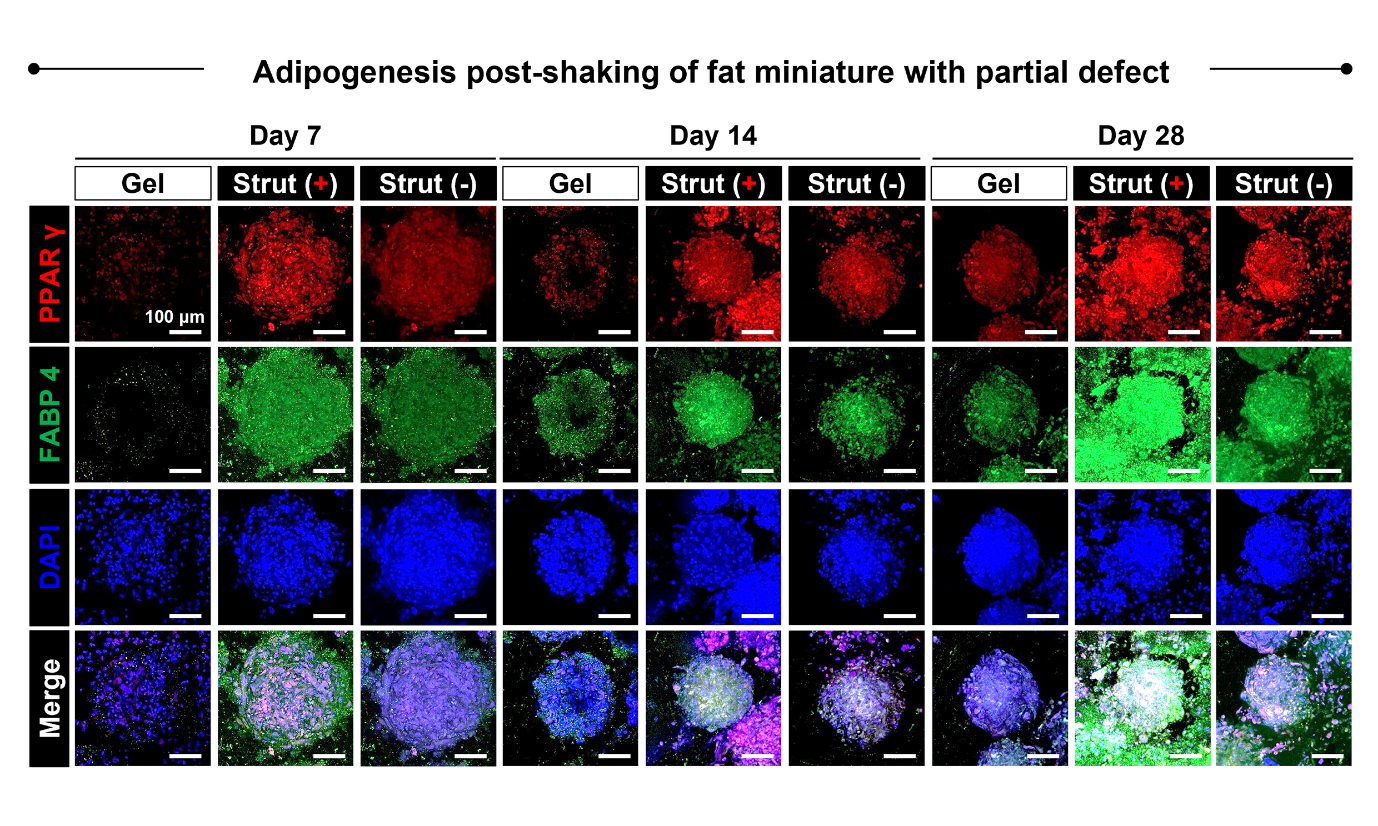
Fig. S8. Pro-adipogenic effect of strut (+) on fat miniatures under shaking conditions.** Strut (+) promotes protein expression of adipogenic markers (red PPARγ and green FABP4) compared to gel and strut (-) when immunohistochemistry is carried out on fat miniatures (blue nucleus) post-shaking following treatment with adipogenic induction media for 28 days (n = 3).

**
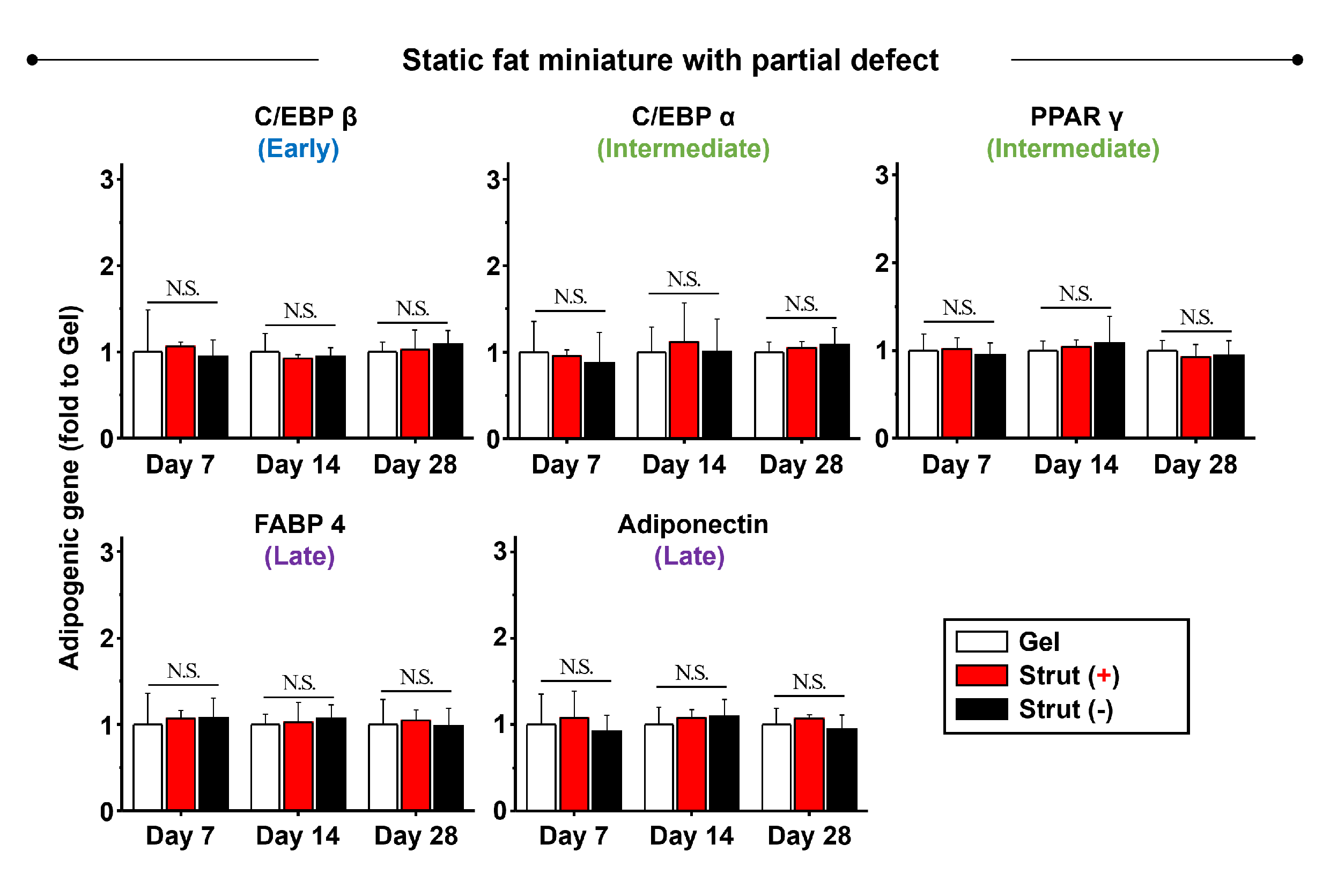
Fig. S9. Lack of membrane and strut-mediated adipogenic changes in static fat miniatures.** In contrast to the shaking condition, the static condition does not allow for the differentiation of the effects of test membrane types as no changes in the adipogenesis of fat miniatures were observed. The gene expression of pro-adipogenic markers (C/EBPβ, C/EBPα, PPARγ, FABP4 and Adiponectin) was determined using qRT-PCR post-culture following treatment with adipogenic induction media for 28 days. Data = mean +/- standard deviation. *p < 0.05, **p < 0.01, and ***p < 0.001 between the lined groups (n = 3). N.S.=not significant.


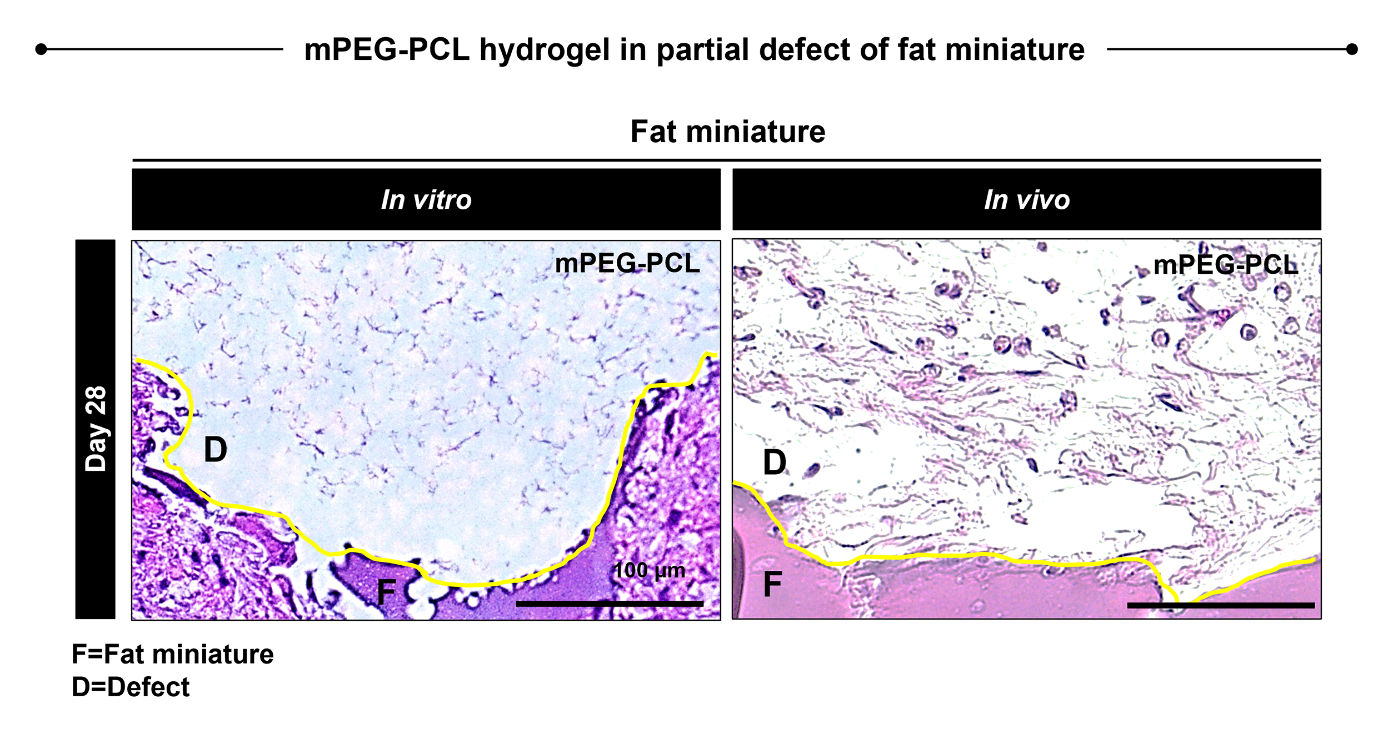


**Fig. S10. mPEG-PCL hydrogel in the partial defect of fat miniature.** The fat miniature was partially defected and cultured *in vitro* (left) or implanted into the subcutaneous back of nude mice for 28 days (right). As a result, the successful invasion of *in vitro* cells and *in vivo* tissues into the defect sites was confirmed as the hydrogel degradation generated the space to fill (yellow line: boundary between the miniature and defect).

**
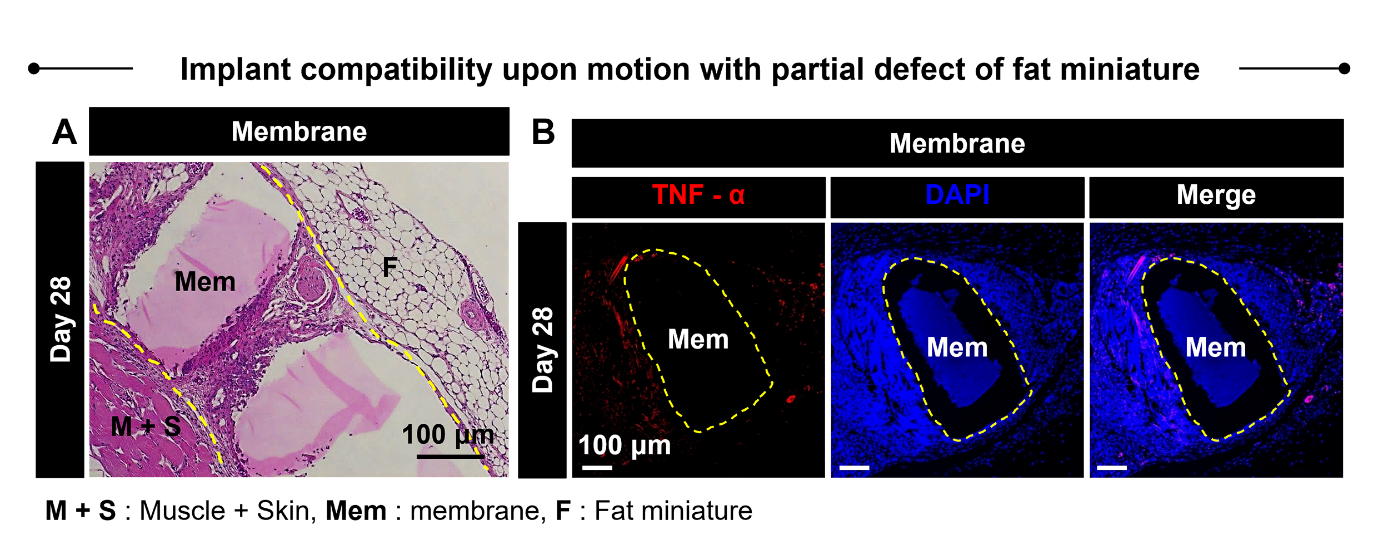
Fig. S11. Implant compatibility of the adipo-conductive membrane.** When the fat miniatures covered with strut (+) were implanted into the subcutaneous back of nude mice for 28 days, **(A)** no visible inflammatory response was observed by H&E staining as supported further by **(B)** negligible expression of TNF-α in immunocytochemistry (yellow line: membrane).


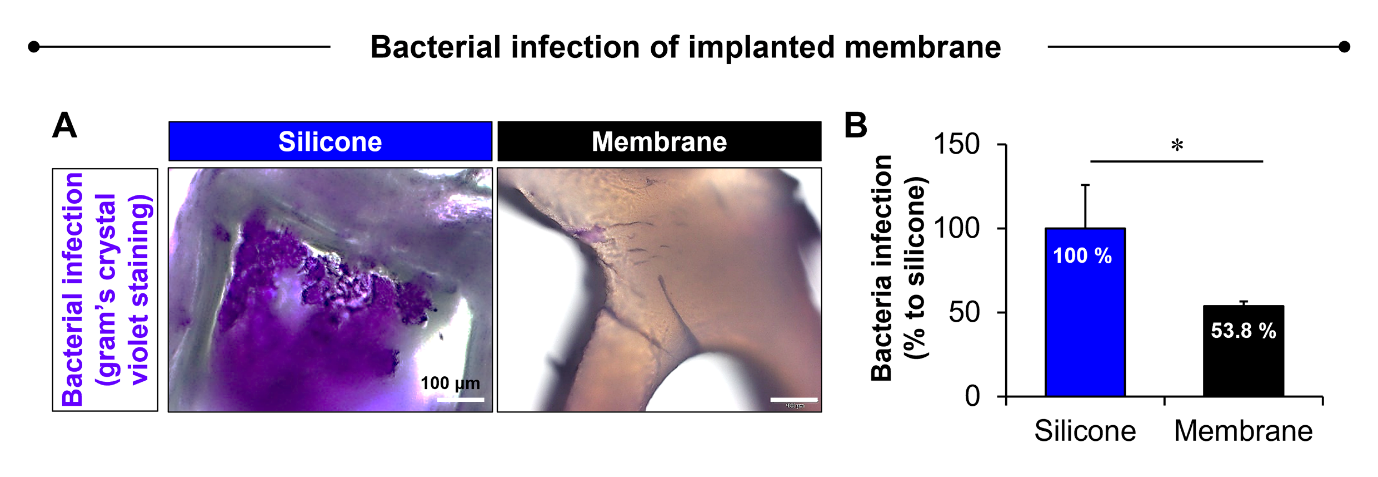


**Fig. S12. Bacterial infection of membrane post implantation with partial defect of fat miniature.** When the fat miniatures were covered with silicone and strut (+) and implanted into the subcutaneous back of nude mice for 28 days, **(A)** no visible bacterial infection was observed compared to silicone by gram’s crystal violet staining. The result was confirmed by **(B)** quantitative analysis of crystal violet absorbance. Data = mean +/- standard deviation. *p < 0.05 between the lined groups (n ≥ 3 in all analyses). N.S.= not significant.

**
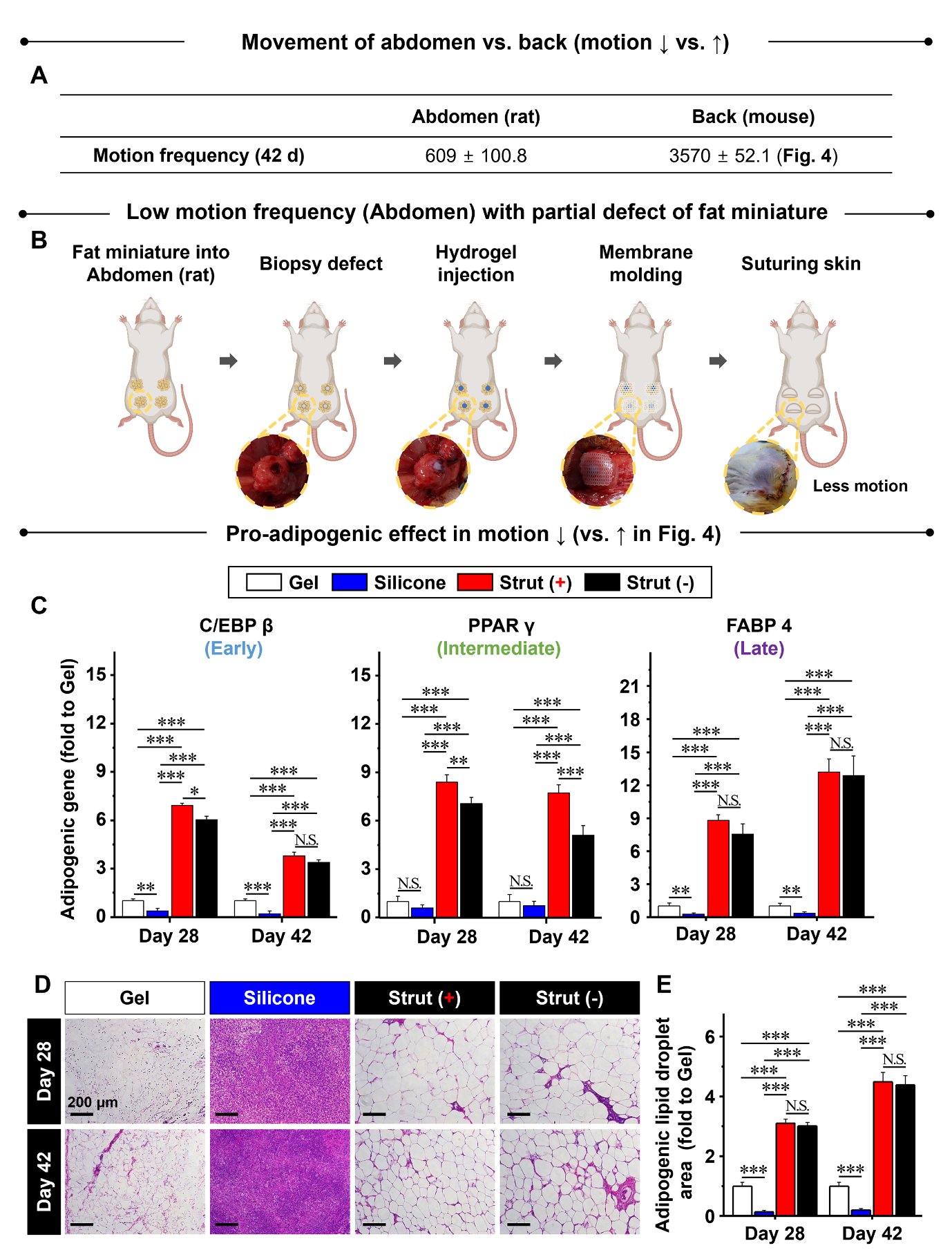
Fig. S13. Insufficient adipogenic effects of low motion frequency on fat miniature under abdomen implantation (A)** The motion frequency of the abdomen is six times less than that of back according to the pedometer recording. Thus, in order to compare the adipogenic effect of low motion frequency with that of high motion frequency (Fig. . 4), **(B)** fat miniatures were implanted into the mouse abdomen for 42 days after being partial defected with hydrogel filing and the defects being covered without (gel) or with test membranes (silicone and strut +/-). **(C)** Although the overall trend in the gene expression of pro-adipogenic markers (C/EBPβ, PPARγ, and FABP4) was similar between the implants in the abdomen and back (Fig. 4) according to the qRT-PCR analyses, differences between the test groups were not significant in some cases including the expression of C/EBPβ and FABP4 between strut (+) and (-) as well as the PPARγ expression between gel and silicone, indicating insufficient adipogenic stimulation in low-motion frequency regions. **(D)** These results are also confirmed by comparisons of the adipogenic lipid droplet areas among the test groups with **(E)** quantitative image analysis. Fibrotic tissue formation by silicone hinders adipogenesis as seen in the back implant. Data = mean +/- standard deviation. *p < 0.05, **p < 0.01, and ***p < 0.001 between the lined groups (n = 4). N.S. = not significant.

**Table S1.** DSC analyses of the membrane polymer (6arm 96%PCL–4%PGMA) before and after crosslinking


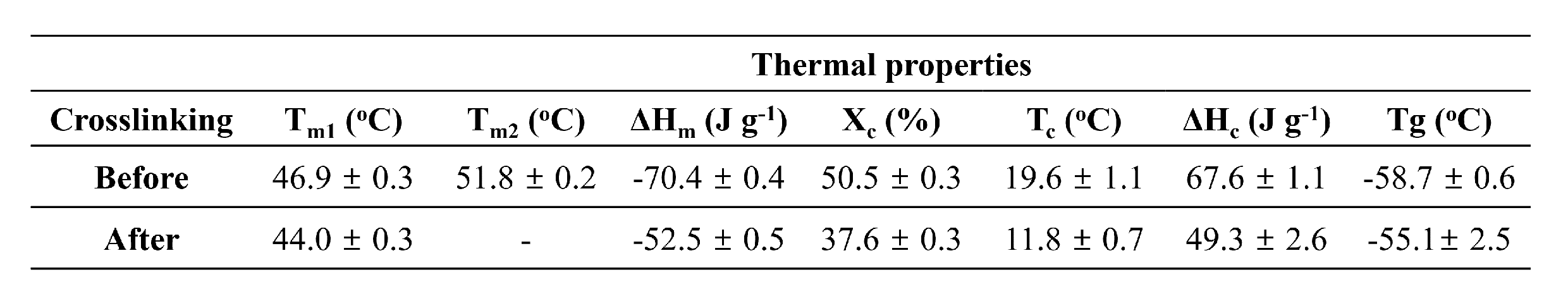


**Table S2.** Comparison of the tensile properties of silicone and the membrane polymer


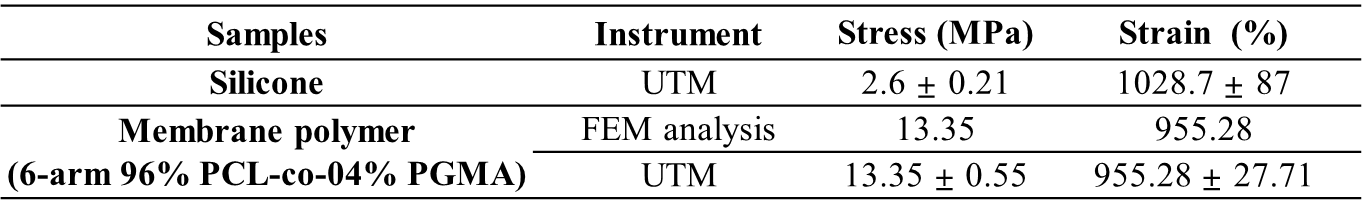


**Table S3.** Comparison of degradation of the membrane polymer with silicone

**
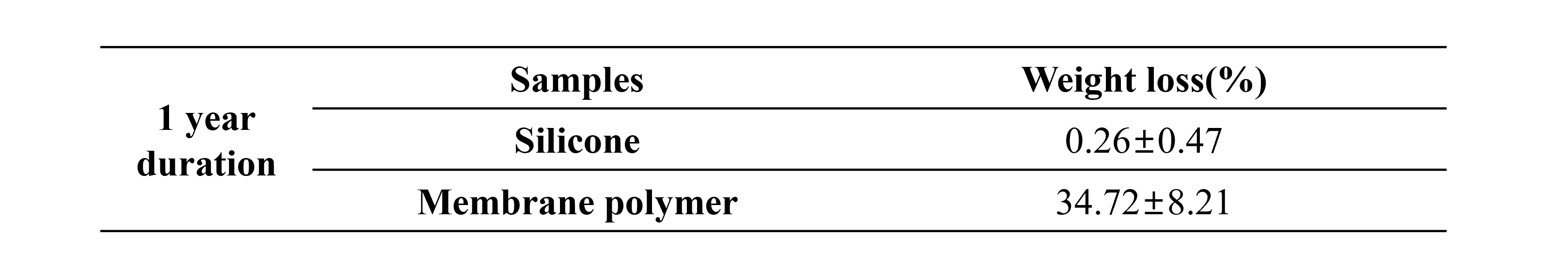
**

**Table S4.** Standard grade of intracutaneous reactivity

**
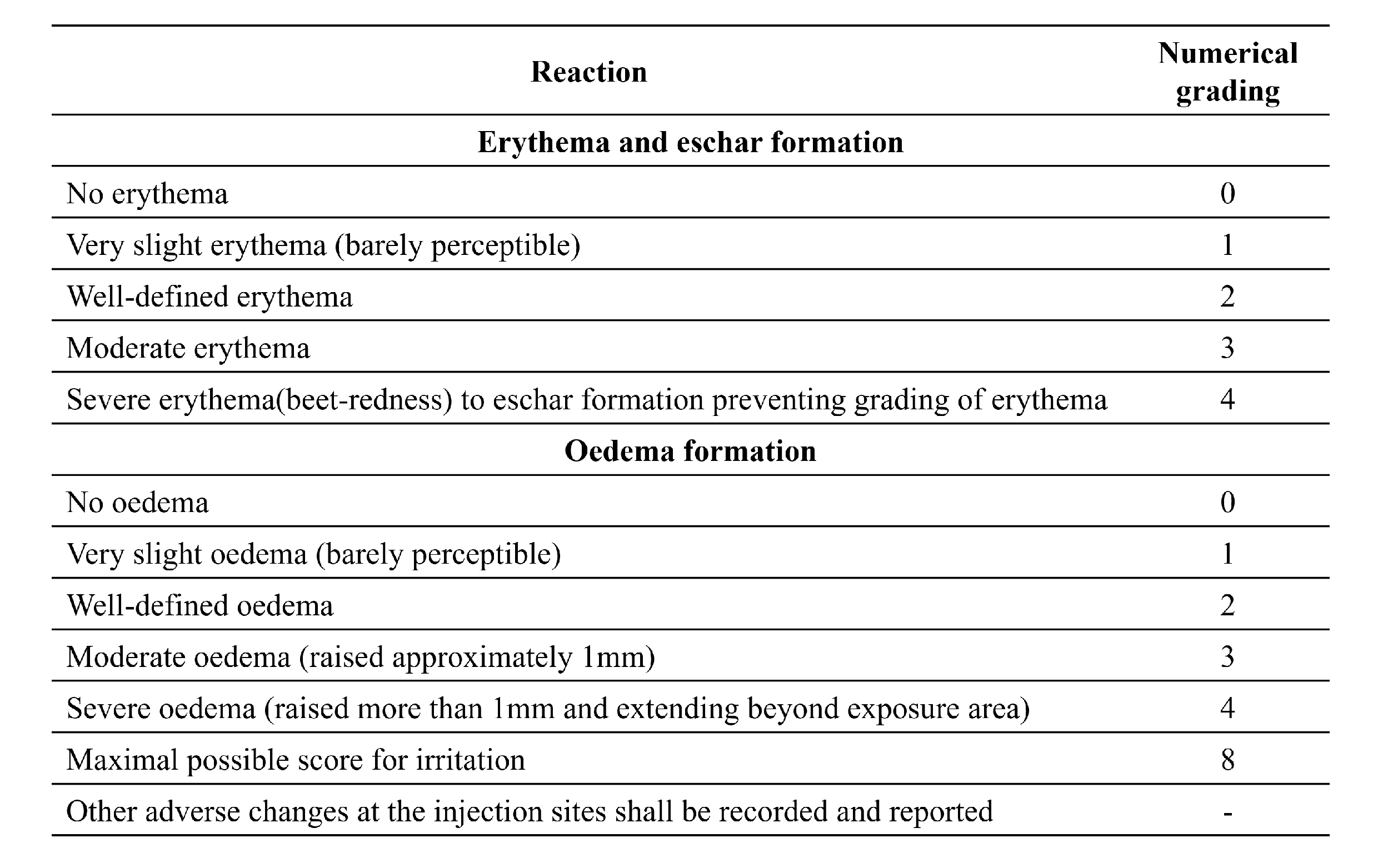
**

**Table S5.** Intracutaneous reactivity of the membrane polymer in saline

**
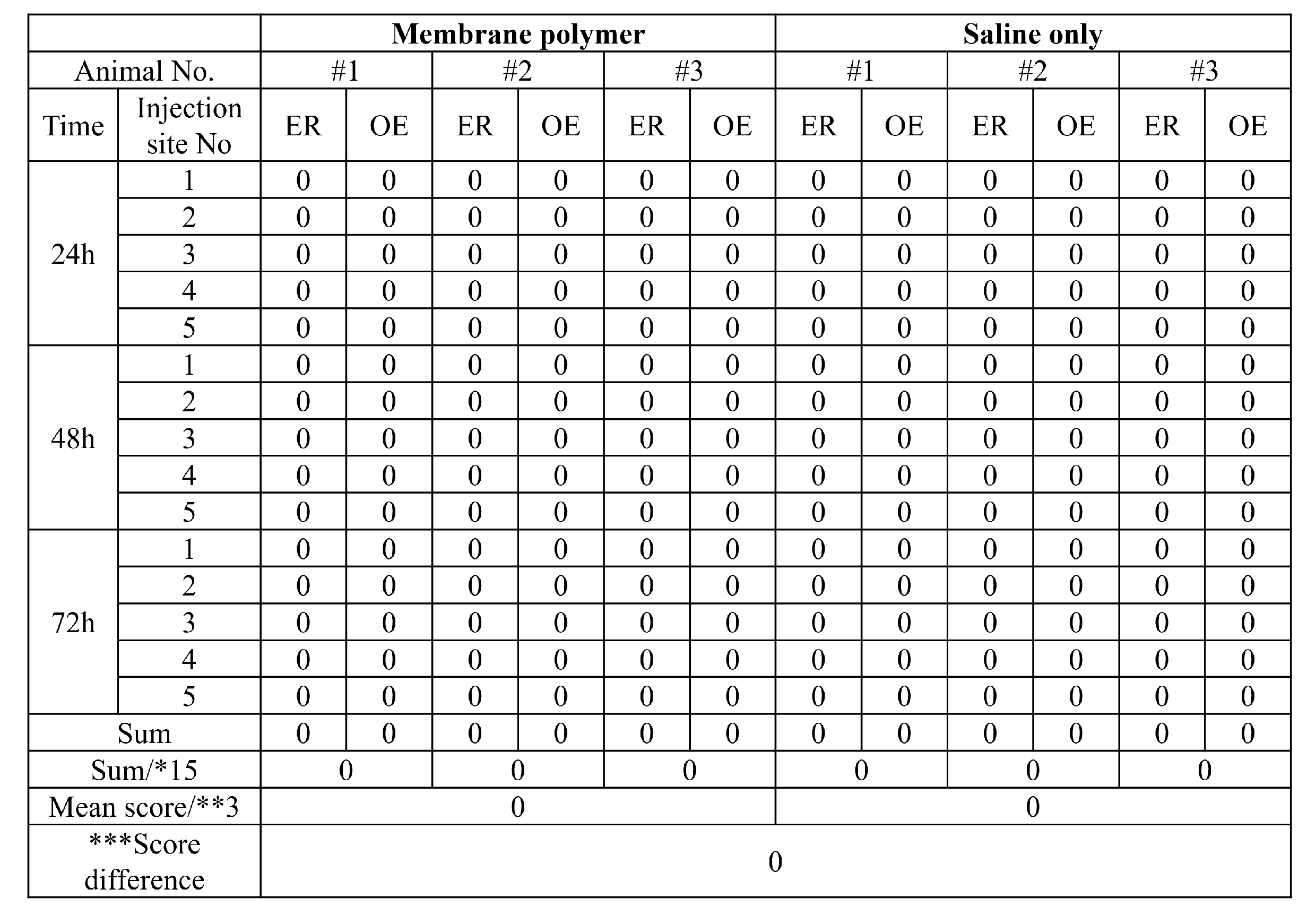
**

ER (erythema), OE (oedema)

* 15 = 3 [scoring time point (24, 48, and 72h)] X 5 (injection sites)

** 3 = Number of animals

*** Score difference = Membrane polymer score – Saline only score

**Table S6.** Intracutaneous reactivity of the membrane polymer in cottonseed oil

**
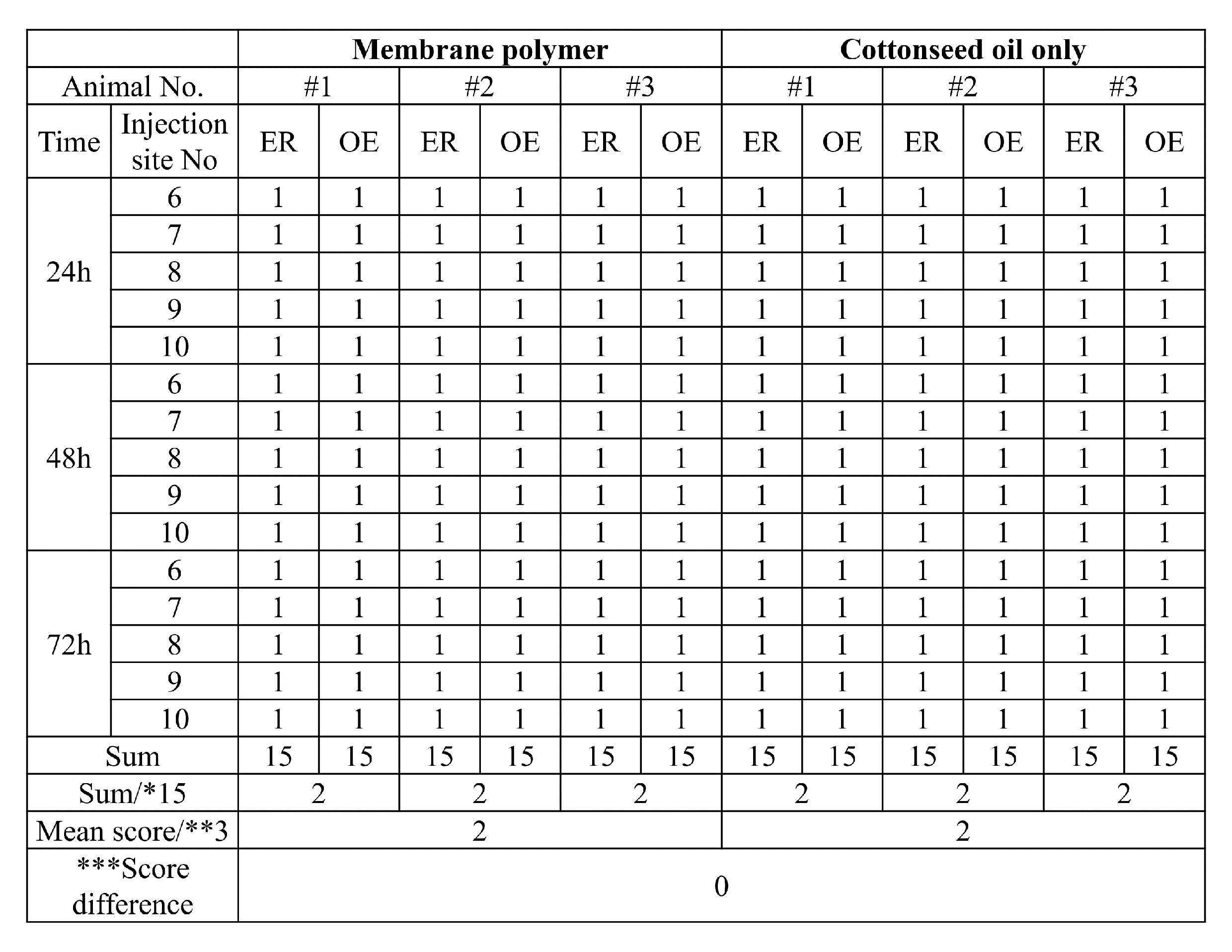
**

ER (erythema), OE (oedema)

* 15 = 3 [scoring time point (24, 48, and 72h)] X 5 (injection sites)

** 3 = Number of animals

*** Score difference = Membrane polymer score – Cottonseed oil only score

**Table S7.** Standard scoring for hemolytic index and grade

**
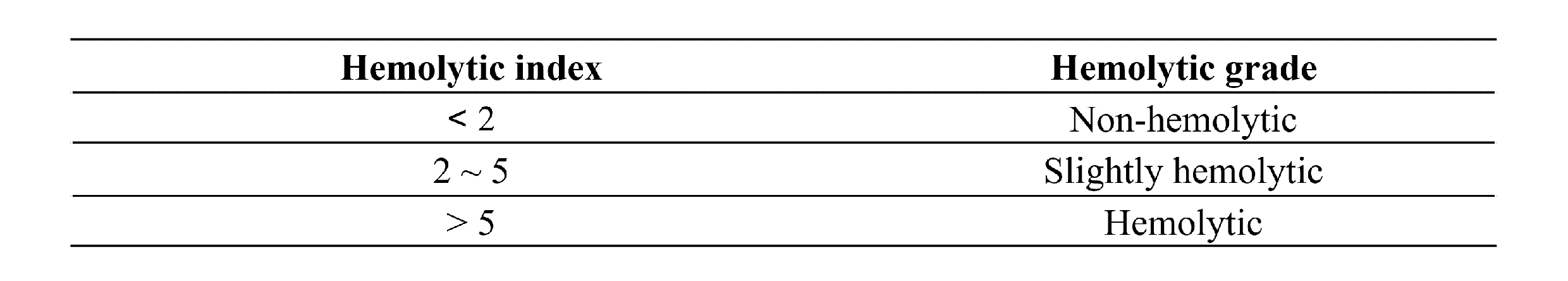
**

**Table S8**. Blood compatibility of the membrane polymer

**
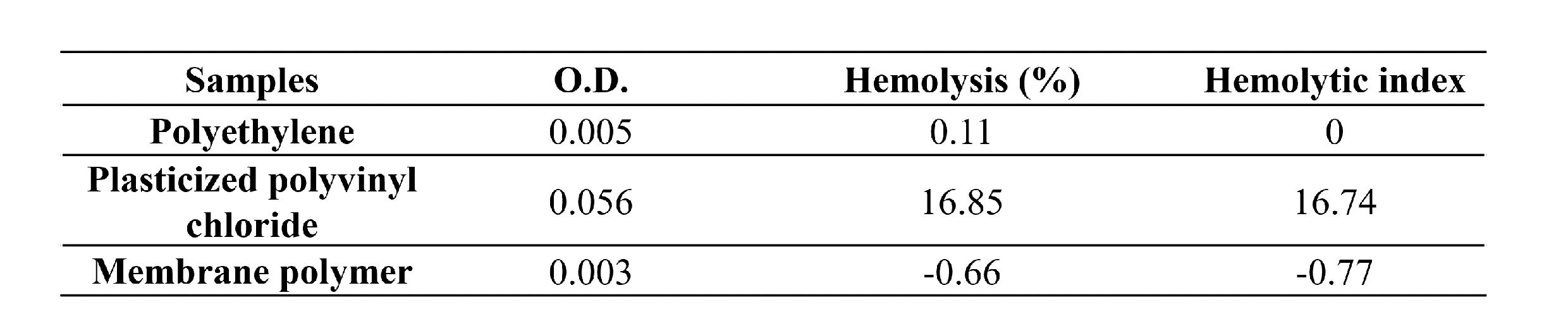
**

**Table S9.** DSC analysis of the mPEG-PCL hydrogel


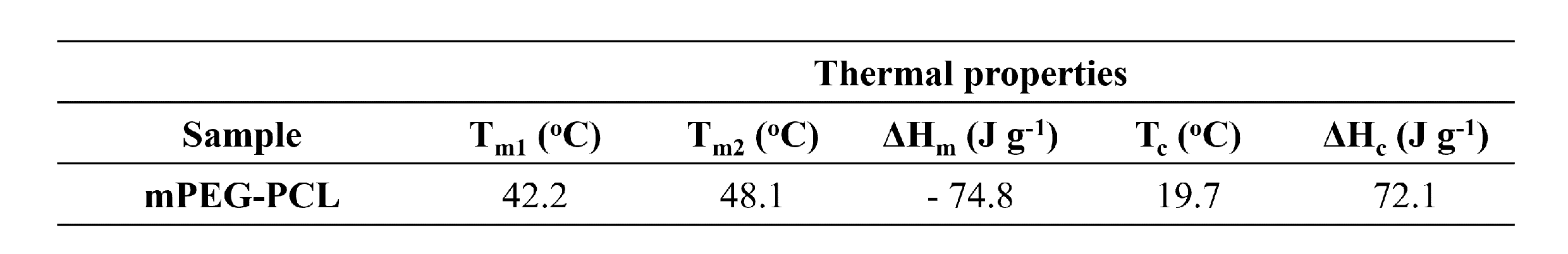


**Table S10.** qRT-PCR primer list


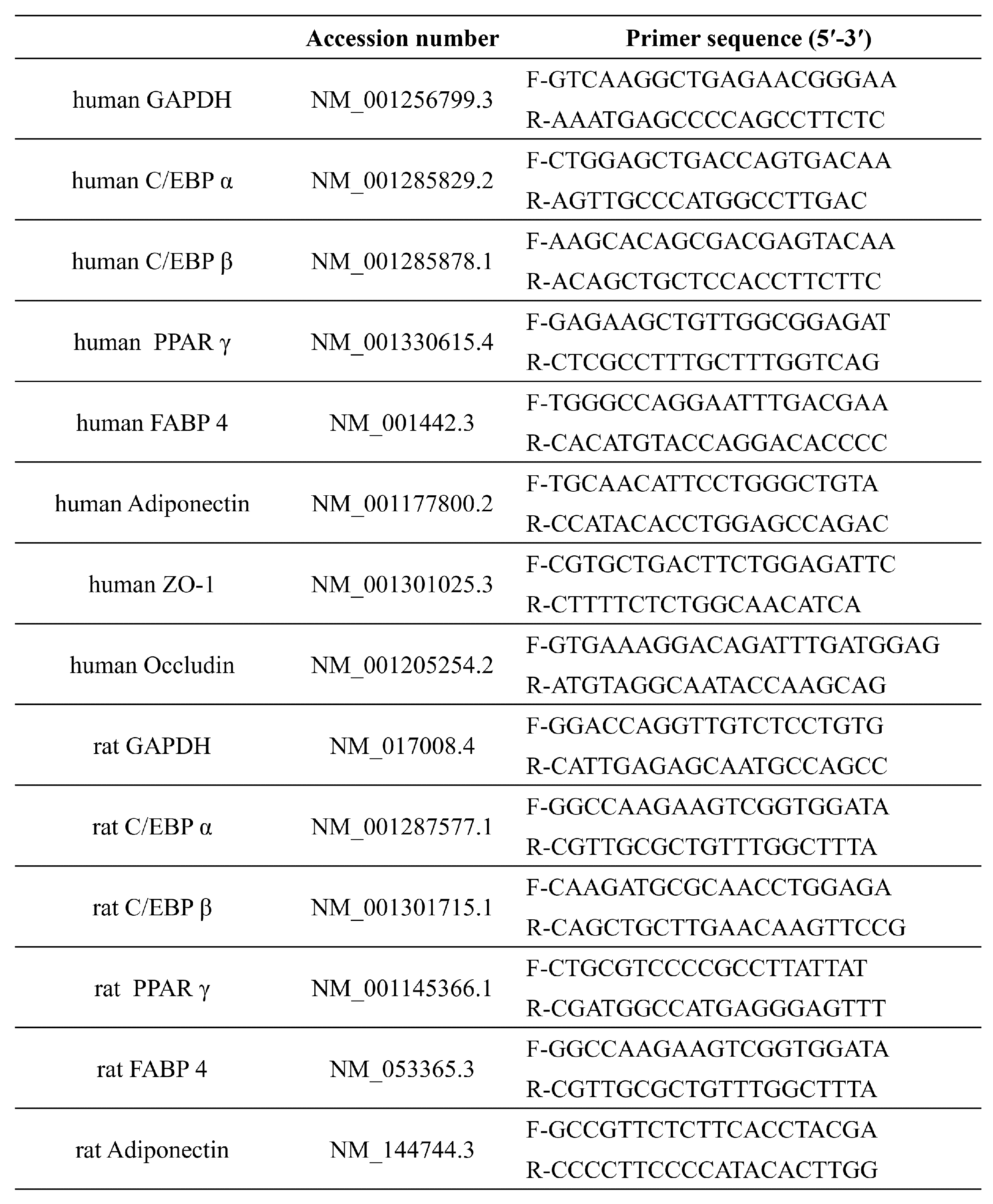

Supplement: Supplementary Materials — Figs. S1 to S13 Tables S1 to S10 Movies S1 to S5 [file research.0137.f1.zip › [Bmemb]SupplementaryRevision-submit.docx]
