# Supplementary figures and images for: Body-Shaping Membrane to Regenerate Breast Fat by Elastic Structural Holding
Source: Research (Wash D C). 2023 May 10;6:0137. doi: 10.34133/research.0137 (PMC10204741; doi:10.34133/research.0137)

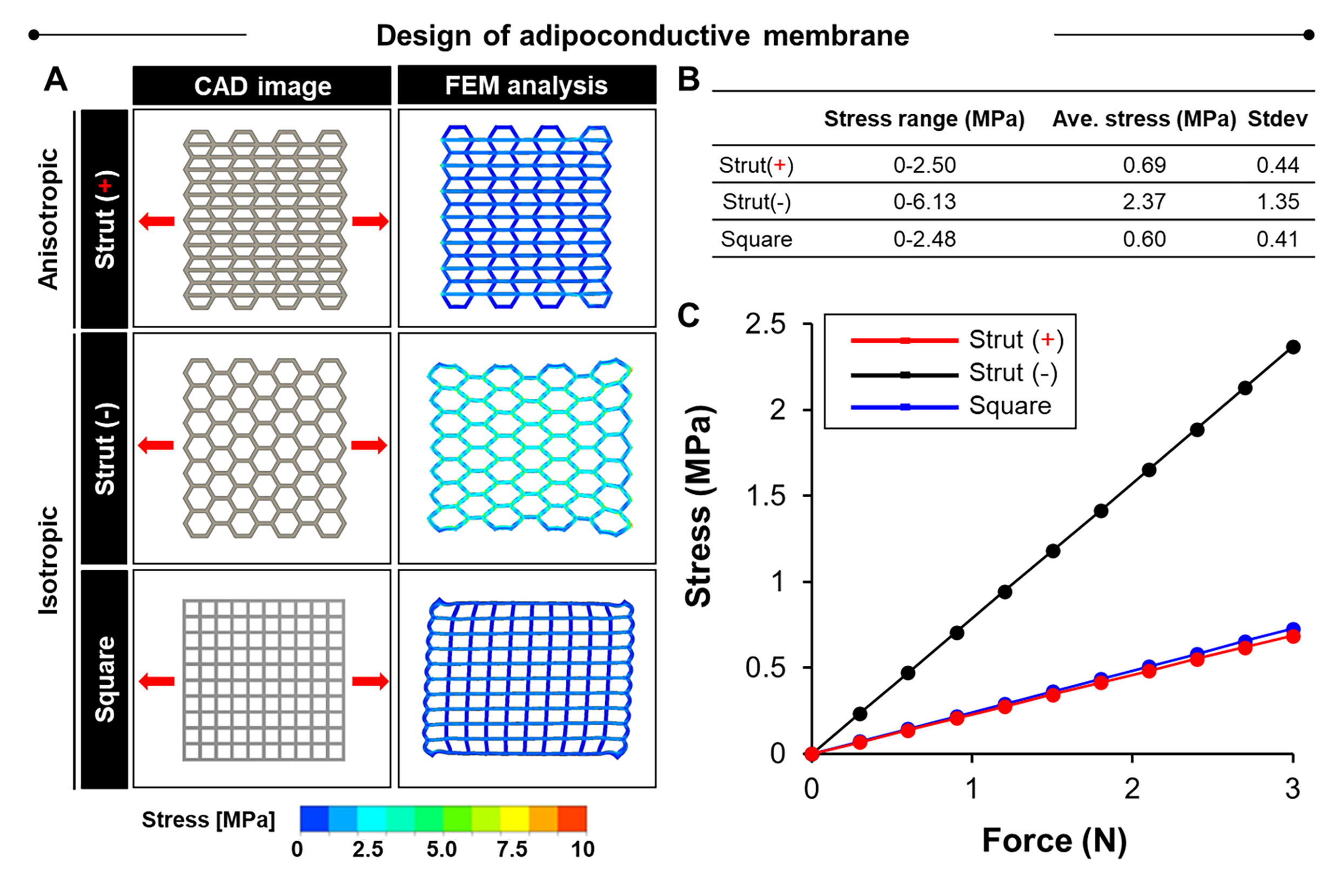

Supplement: Supplementary Materials — Figs. S1 to S13 Tables S1 to S10 Movies S1 to S5 [file research.0137.f1.zip › Figure S1.tif]

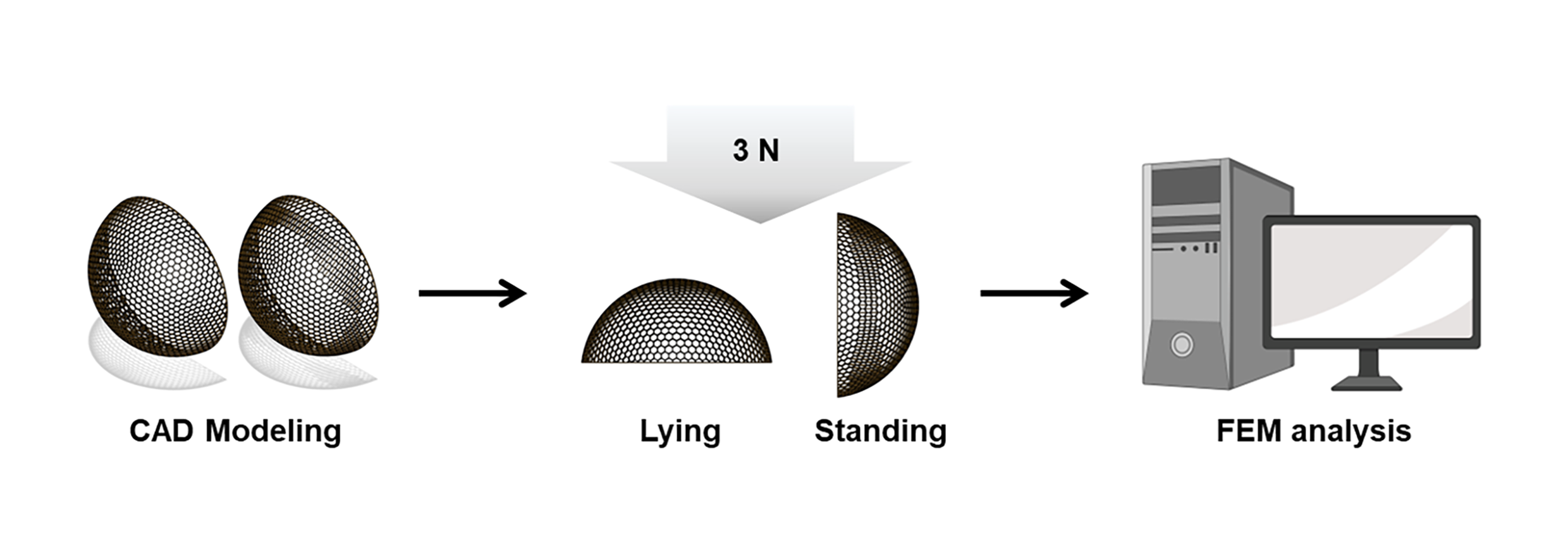

Supplement: Supplementary Materials — Figs. S1 to S13 Tables S1 to S10 Movies S1 to S5 [file research.0137.f1.zip › Figure S2.tif]

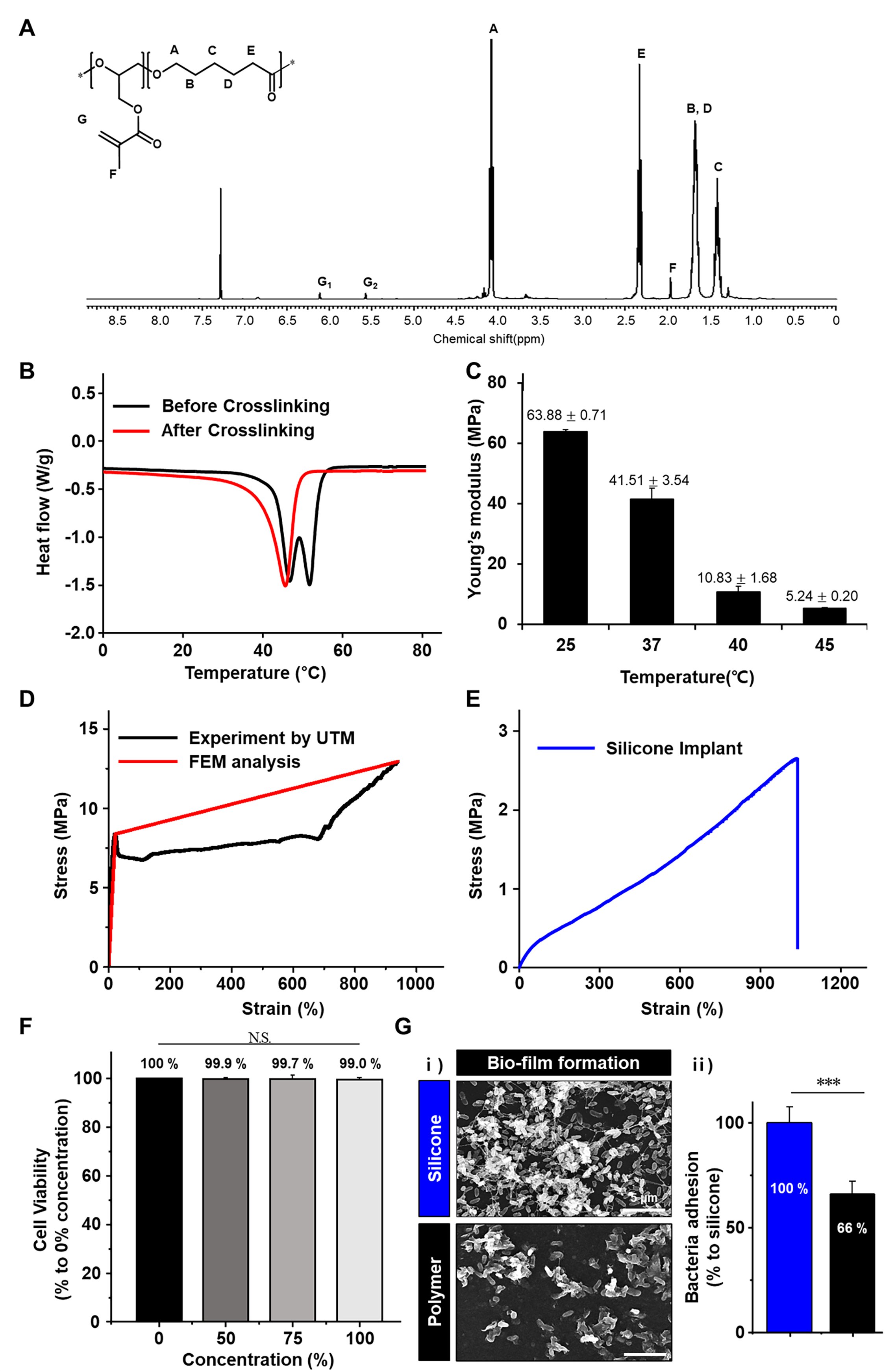

Supplement: Supplementary Materials — Figs. S1 to S13 Tables S1 to S10 Movies S1 to S5 [file research.0137.f1.zip › Figure S3.tif]

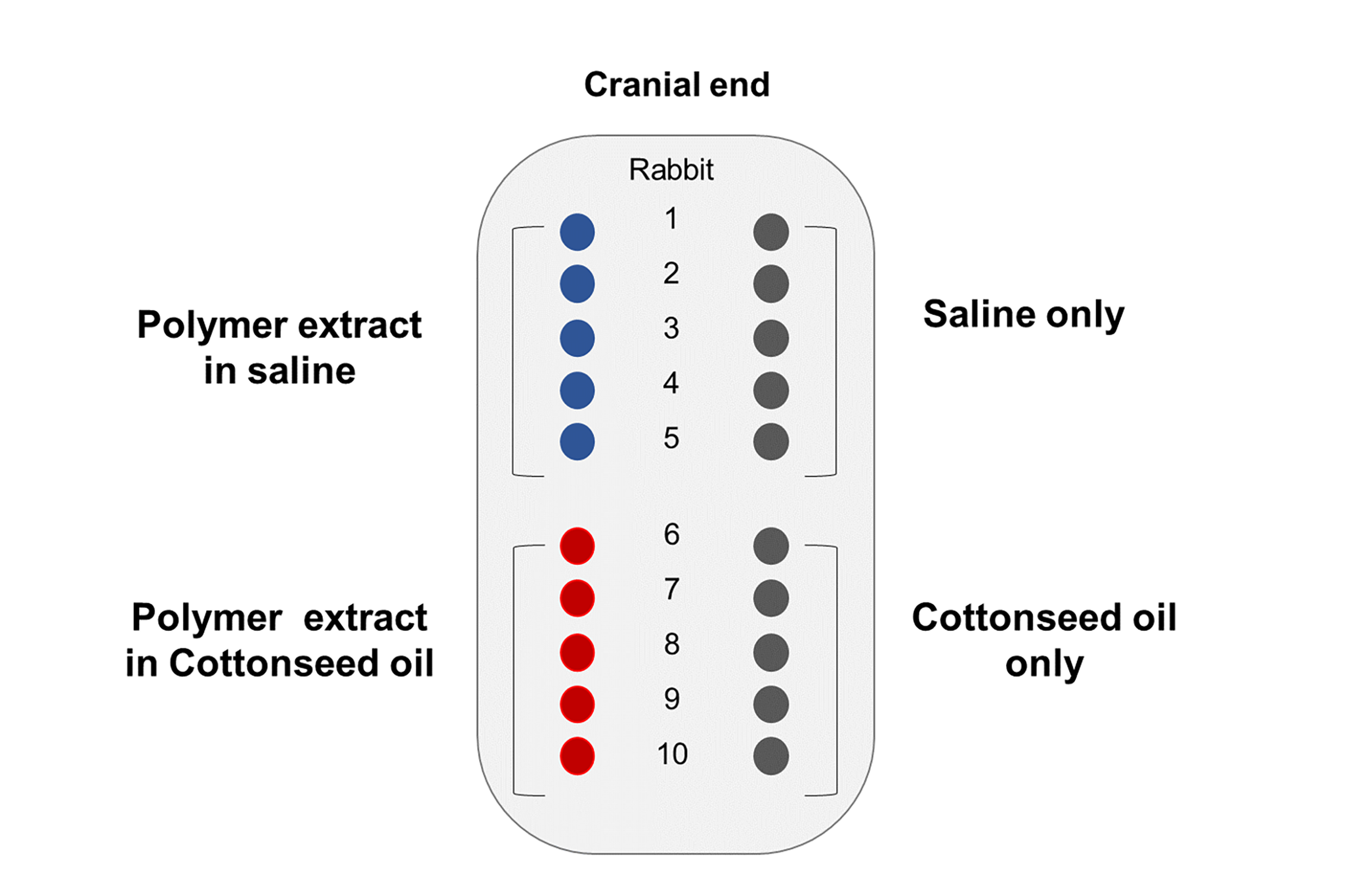

Supplement: Supplementary Materials — Figs. S1 to S13 Tables S1 to S10 Movies S1 to S5 [file research.0137.f1.zip › Figure S4.tif]

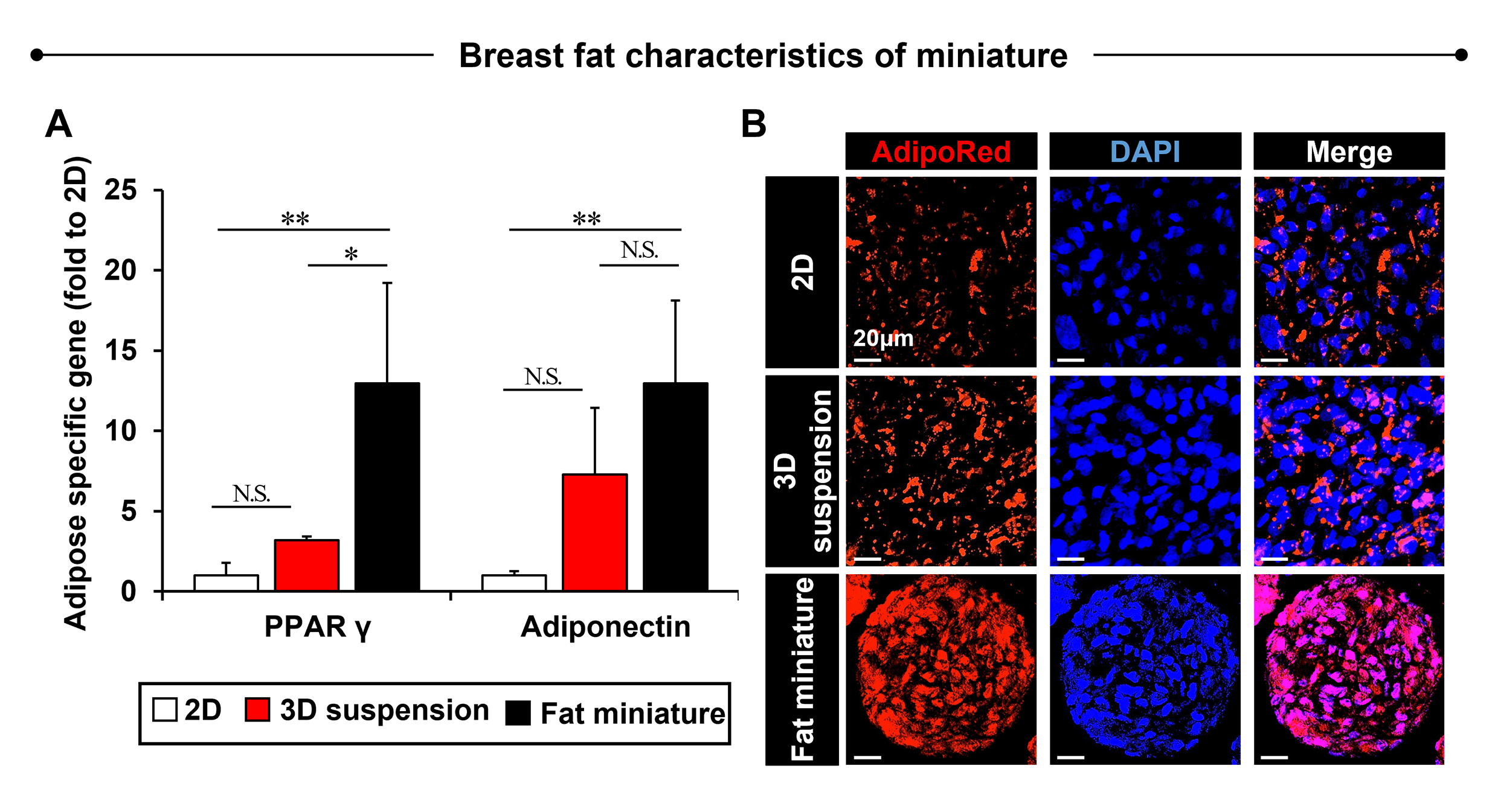

Supplement: Supplementary Materials — Figs. S1 to S13 Tables S1 to S10 Movies S1 to S5 [file research.0137.f1.zip › Figure S5.tif]

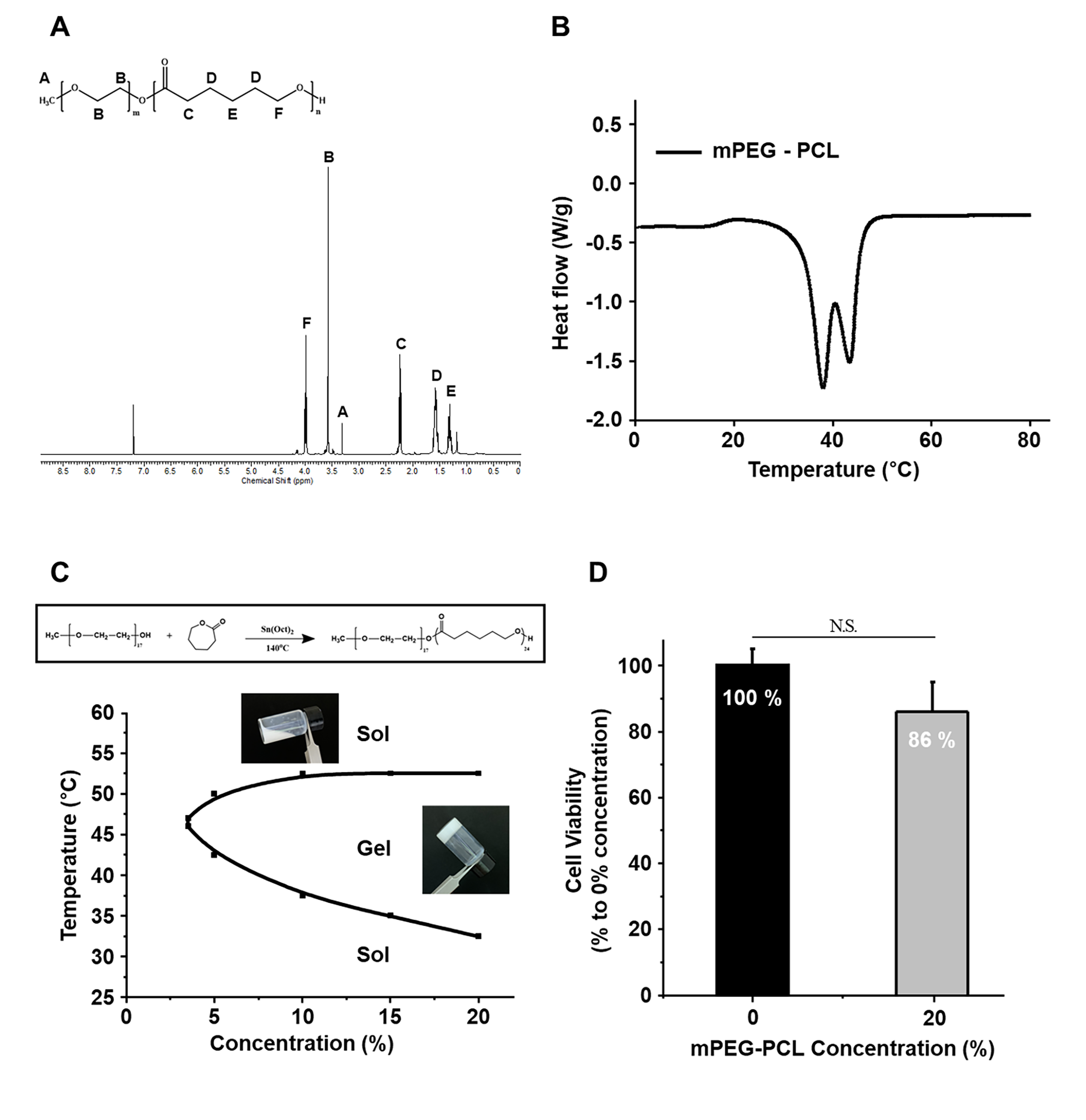

Supplement: Supplementary Materials — Figs. S1 to S13 Tables S1 to S10 Movies S1 to S5 [file research.0137.f1.zip › Figure S6.tif]

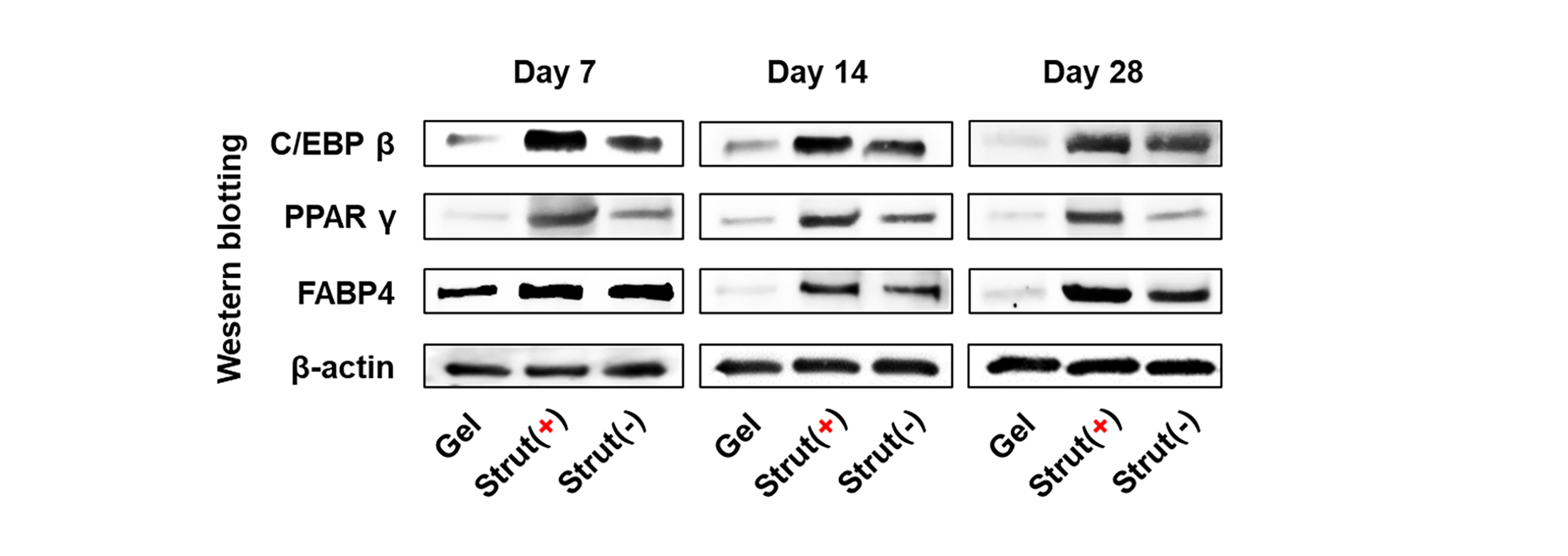

Supplement: Supplementary Materials — Figs. S1 to S13 Tables S1 to S10 Movies S1 to S5 [file research.0137.f1.zip › Figure S7.tif]

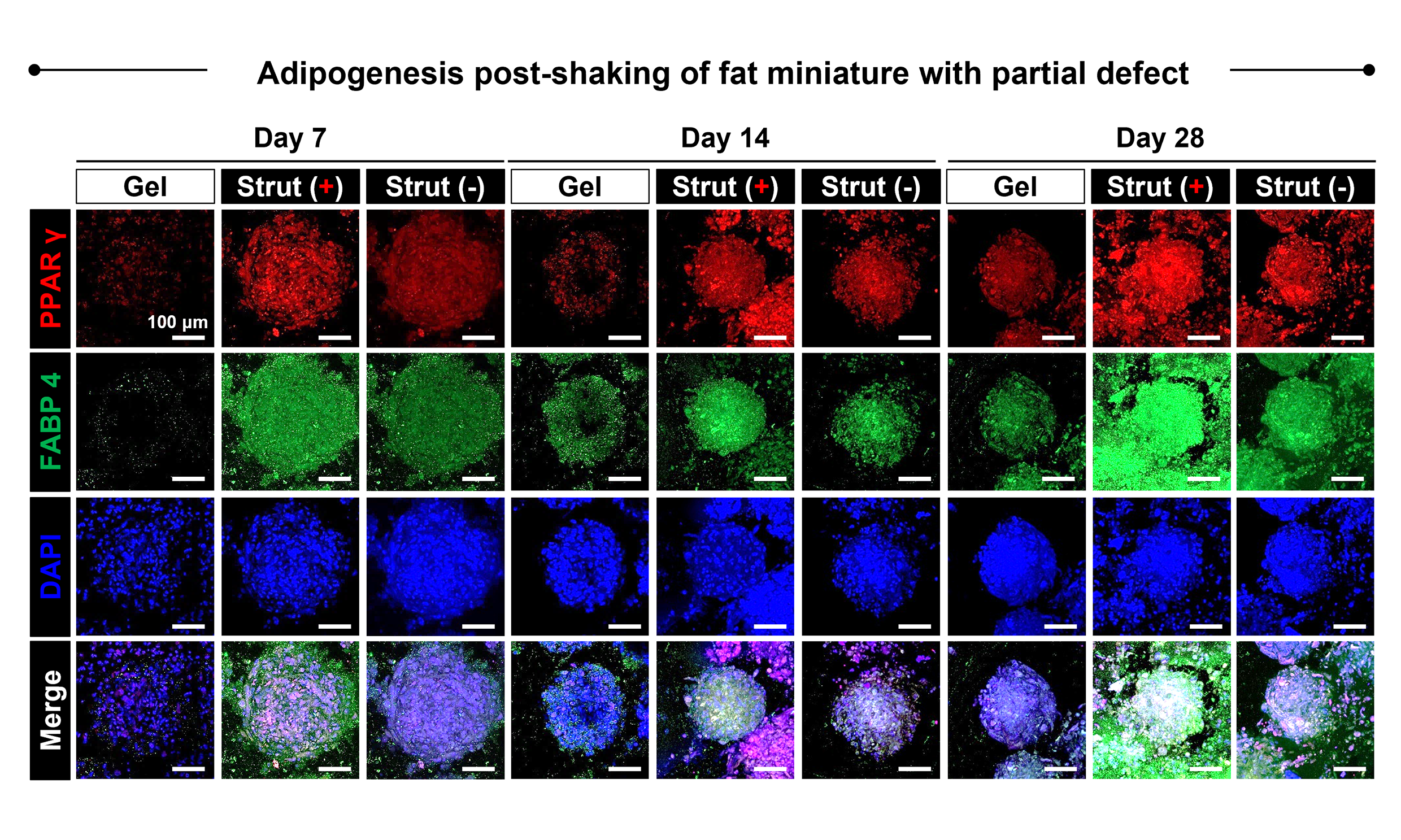

Supplement: Supplementary Materials — Figs. S1 to S13 Tables S1 to S10 Movies S1 to S5 [file research.0137.f1.zip › Figure S8.tif]

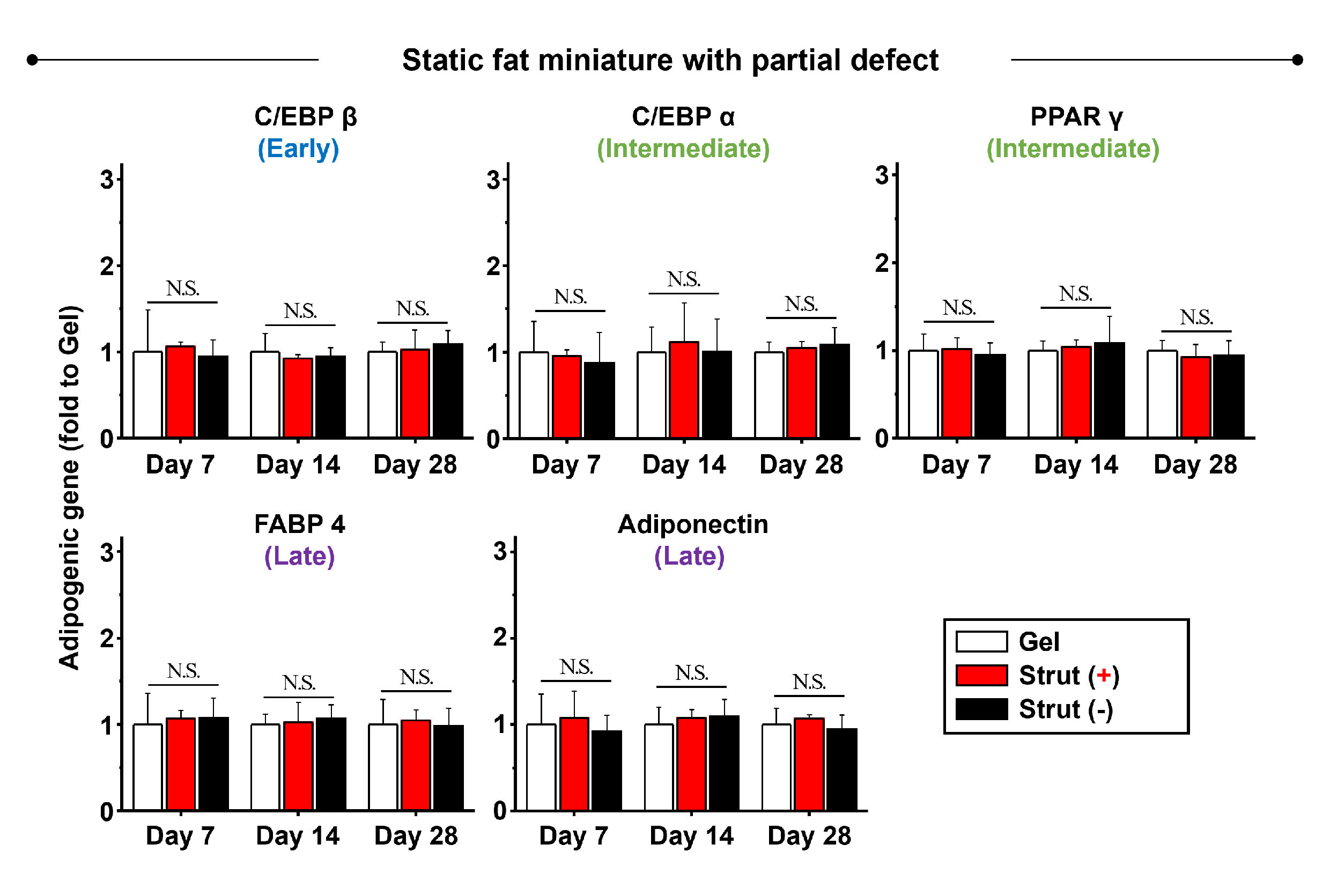

Supplement: Supplementary Materials — Figs. S1 to S13 Tables S1 to S10 Movies S1 to S5 [file research.0137.f1.zip › Figure S9.png]

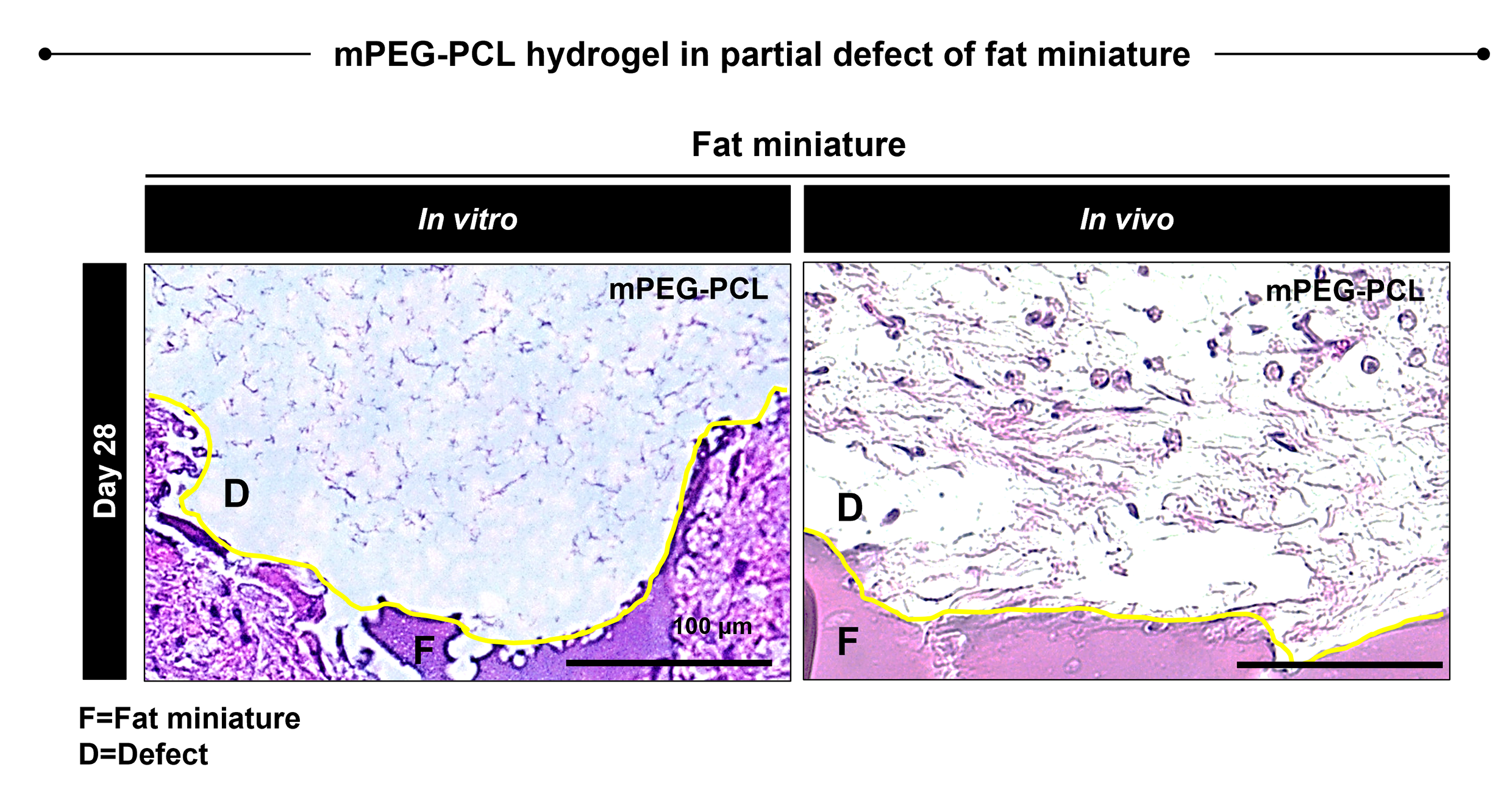

Supplement: Supplementary Materials — Figs. S1 to S13 Tables S1 to S10 Movies S1 to S5 [file research.0137.f1.zip › Figure S10.tif]

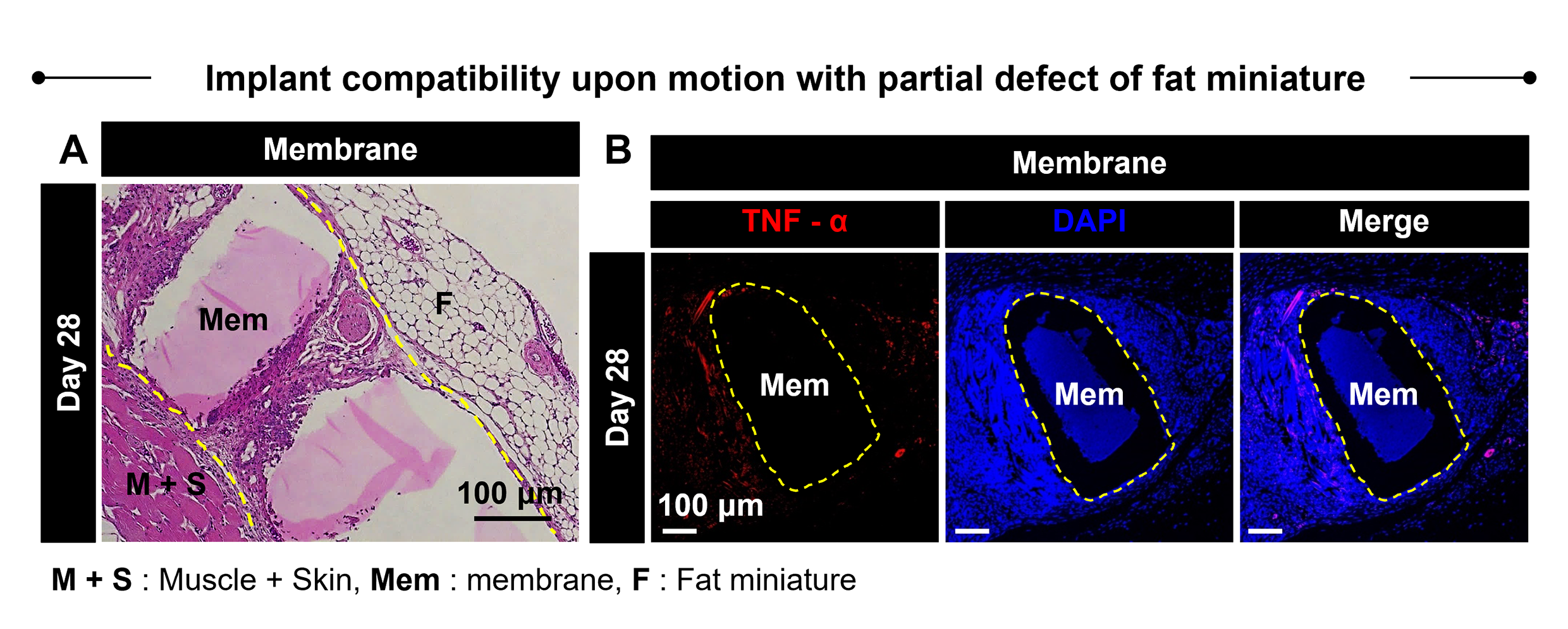

Supplement: Supplementary Materials — Figs. S1 to S13 Tables S1 to S10 Movies S1 to S5 [file research.0137.f1.zip › Figure S11.tif]

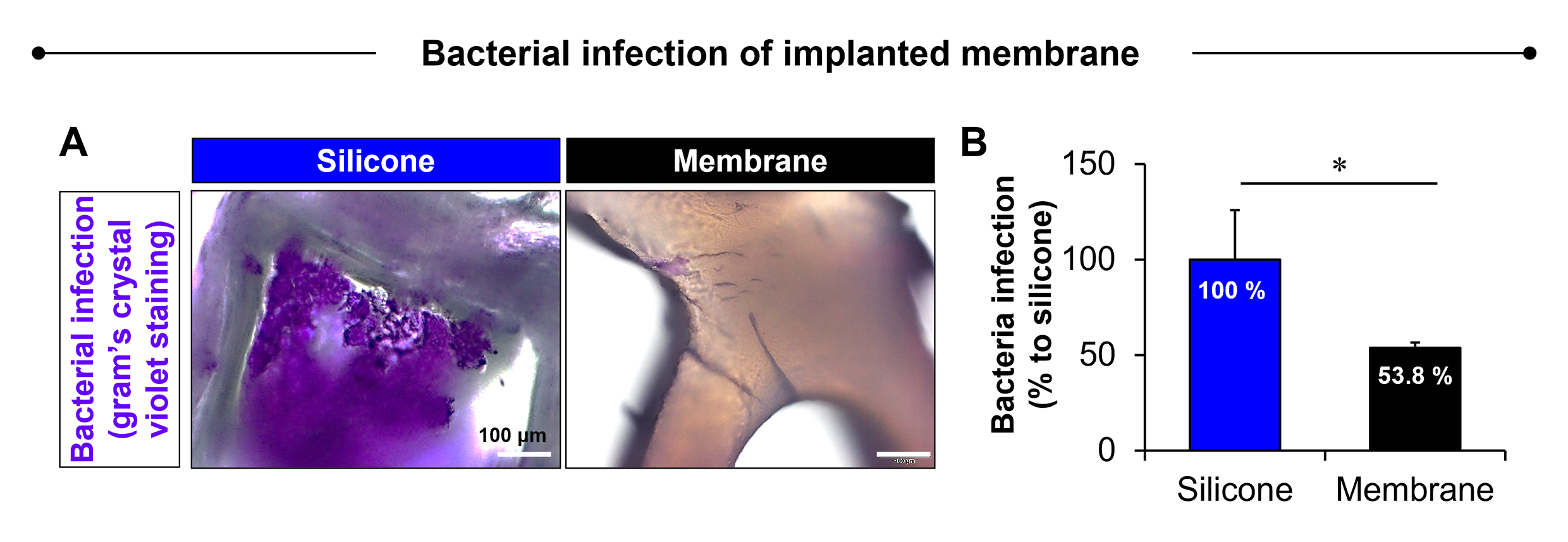

Supplement: Supplementary Materials — Figs. S1 to S13 Tables S1 to S10 Movies S1 to S5 [file research.0137.f1.zip › Figure S12.tif]

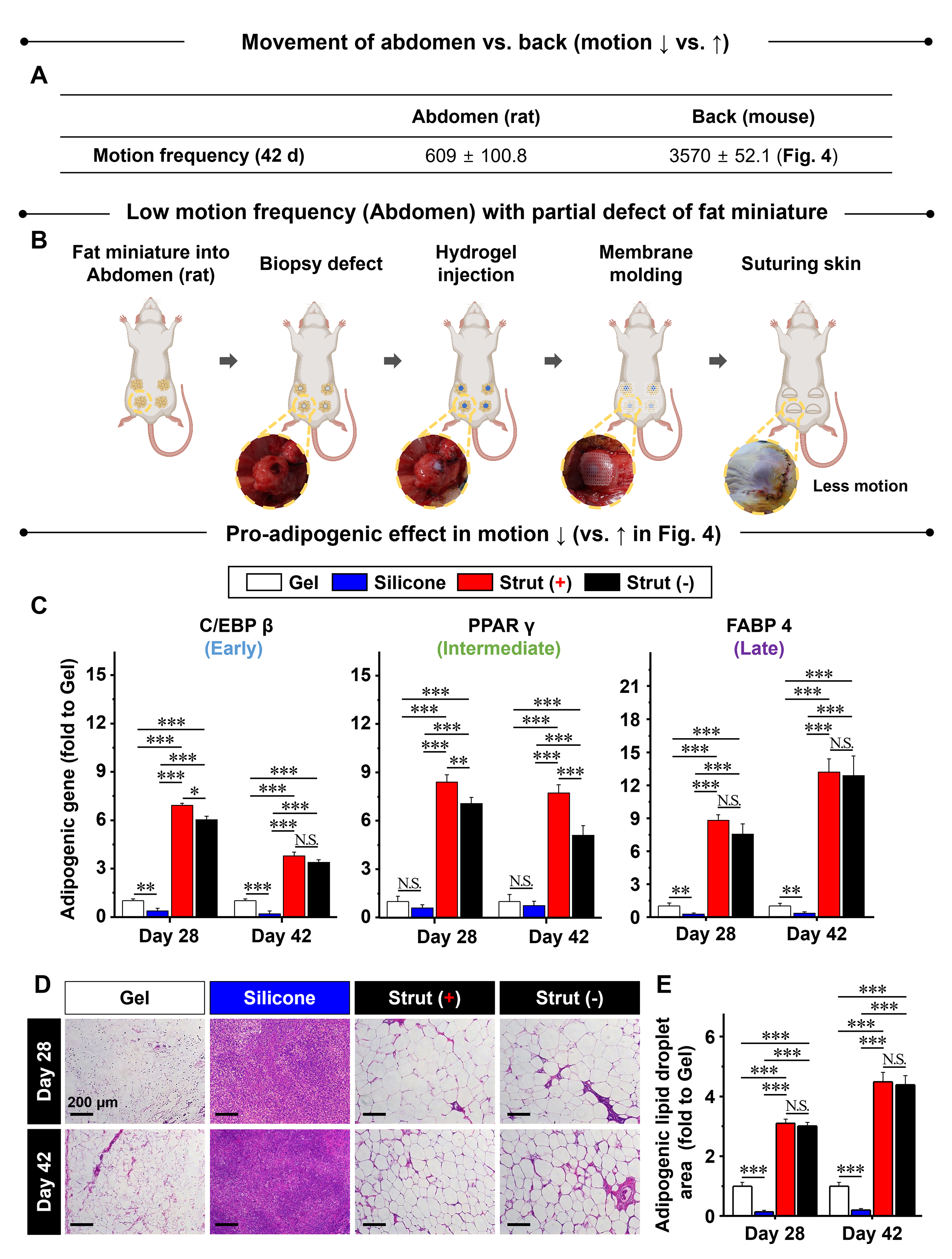

Supplement: Supplementary Materials — Figs. S1 to S13 Tables S1 to S10 Movies S1 to S5 [file research.0137.f1.zip › Figure S13.tif]

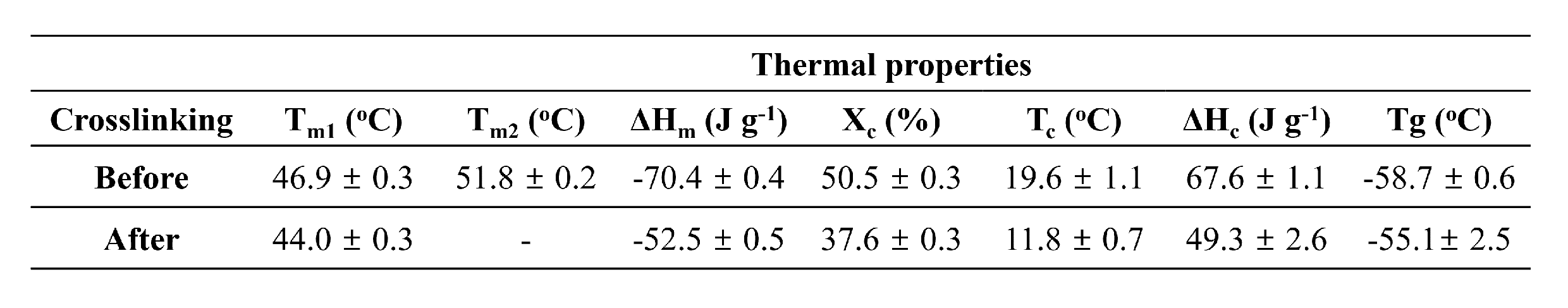

Supplement: Supplementary Materials — Figs. S1 to S13 Tables S1 to S10 Movies S1 to S5 [file research.0137.f1.zip › Table S1.png]

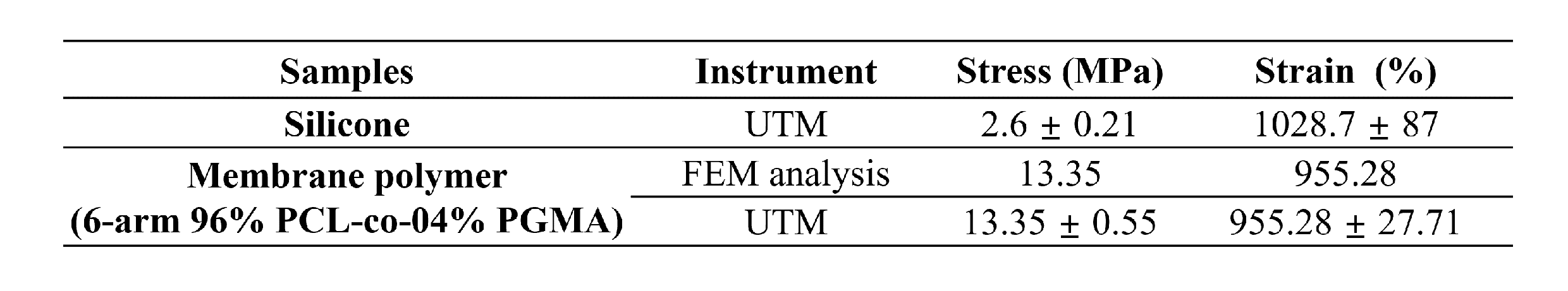

Supplement: Supplementary Materials — Figs. S1 to S13 Tables S1 to S10 Movies S1 to S5 [file research.0137.f1.zip › Table S2.png]

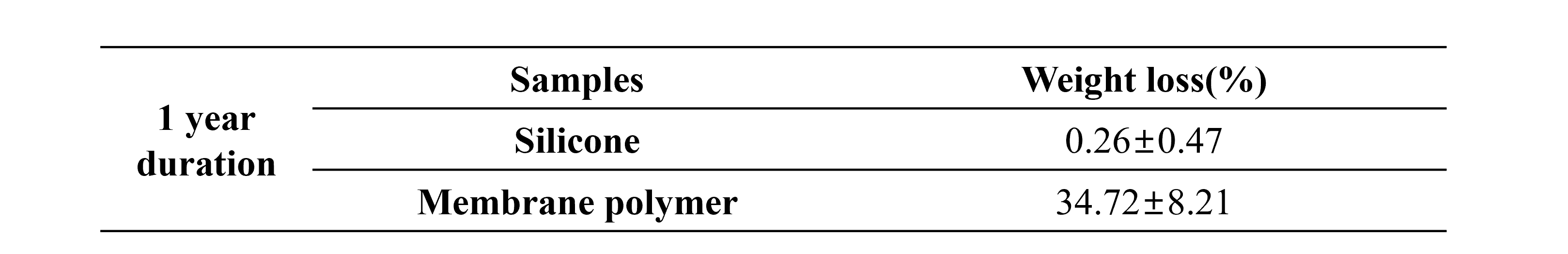

Supplement: Supplementary Materials — Figs. S1 to S13 Tables S1 to S10 Movies S1 to S5 [file research.0137.f1.zip › Table S3.png]

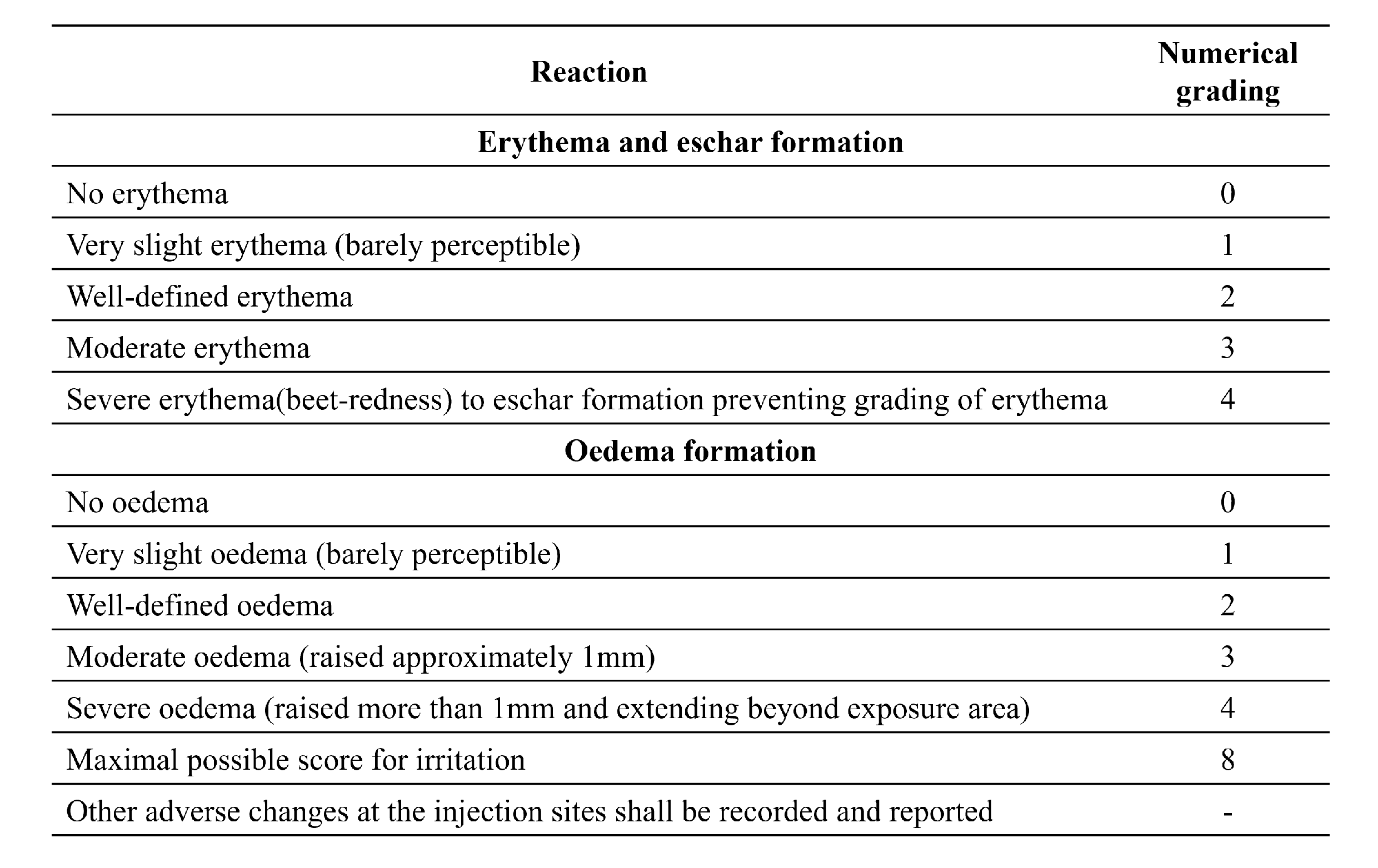

Supplement: Supplementary Materials — Figs. S1 to S13 Tables S1 to S10 Movies S1 to S5 [file research.0137.f1.zip › Table S4.png]

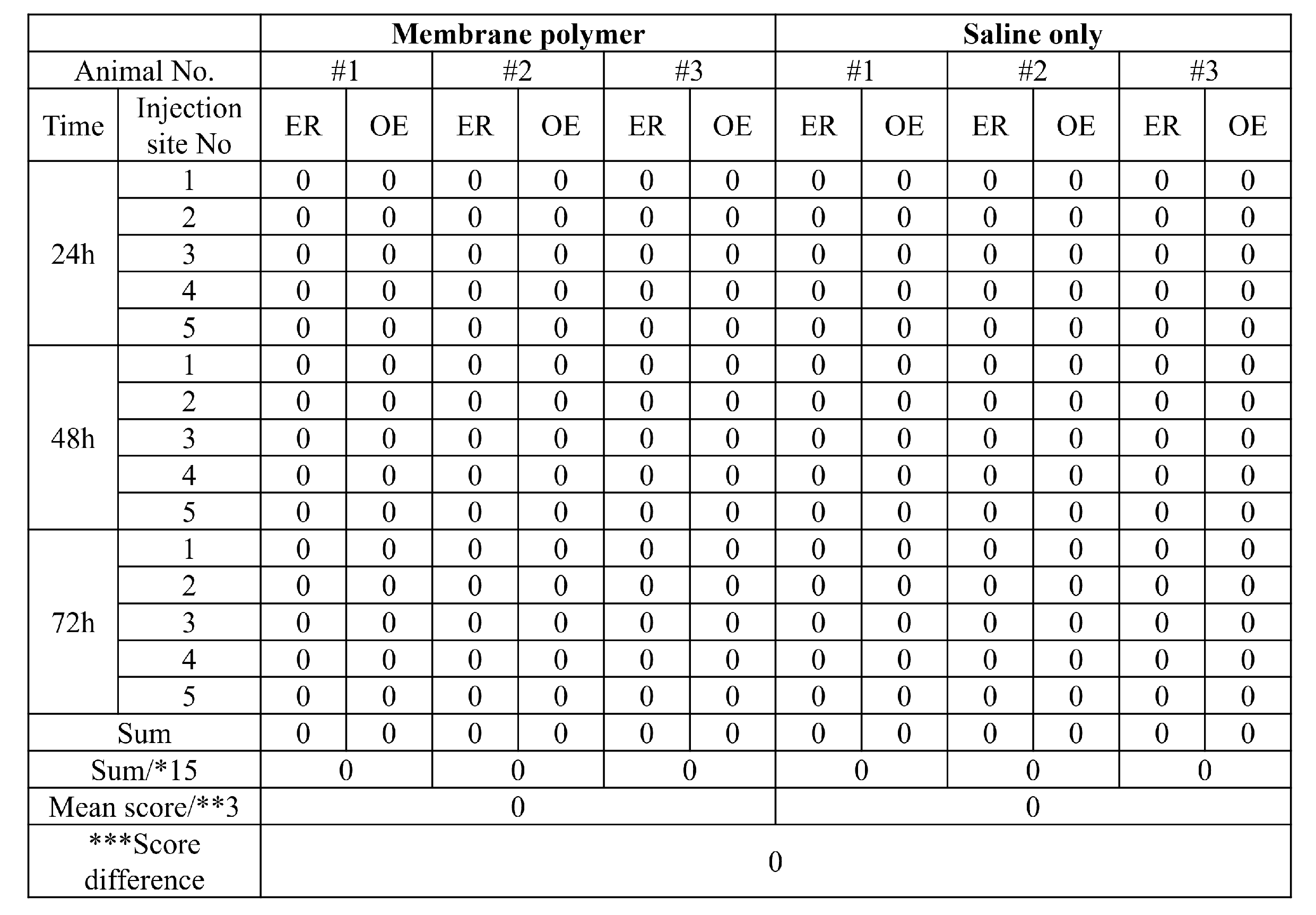

Supplement: Supplementary Materials — Figs. S1 to S13 Tables S1 to S10 Movies S1 to S5 [file research.0137.f1.zip › Table S5.png]

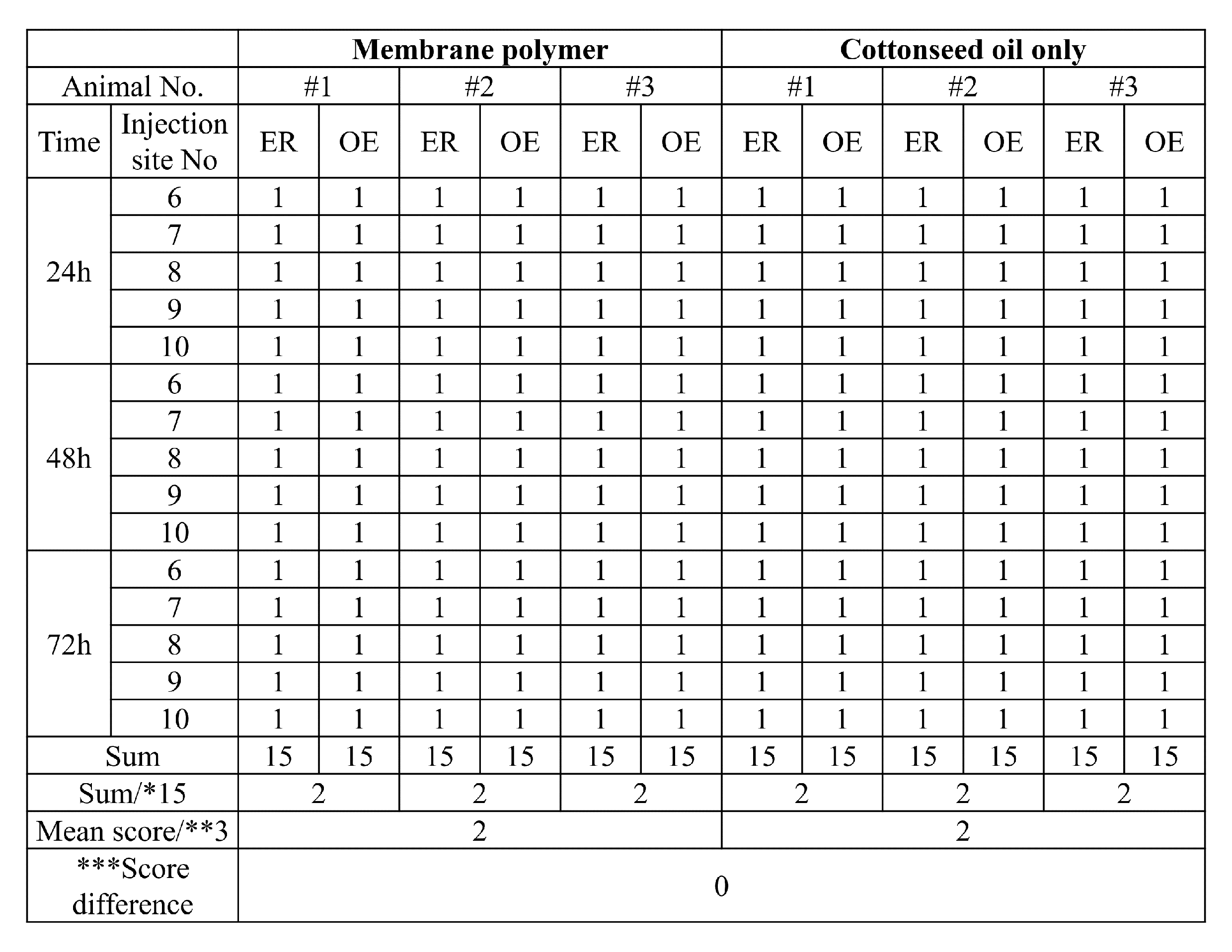

Supplement: Supplementary Materials — Figs. S1 to S13 Tables S1 to S10 Movies S1 to S5 [file research.0137.f1.zip › Table S6.png]

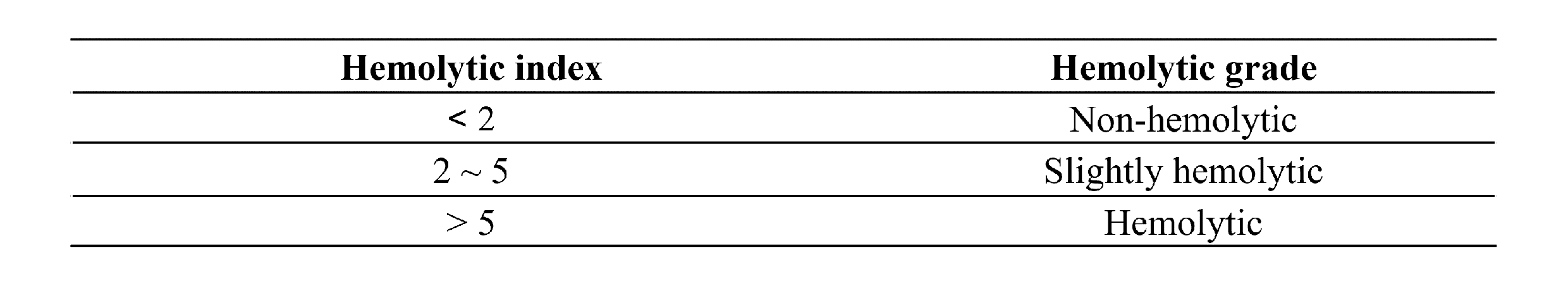

Supplement: Supplementary Materials — Figs. S1 to S13 Tables S1 to S10 Movies S1 to S5 [file research.0137.f1.zip › Table S7.png]

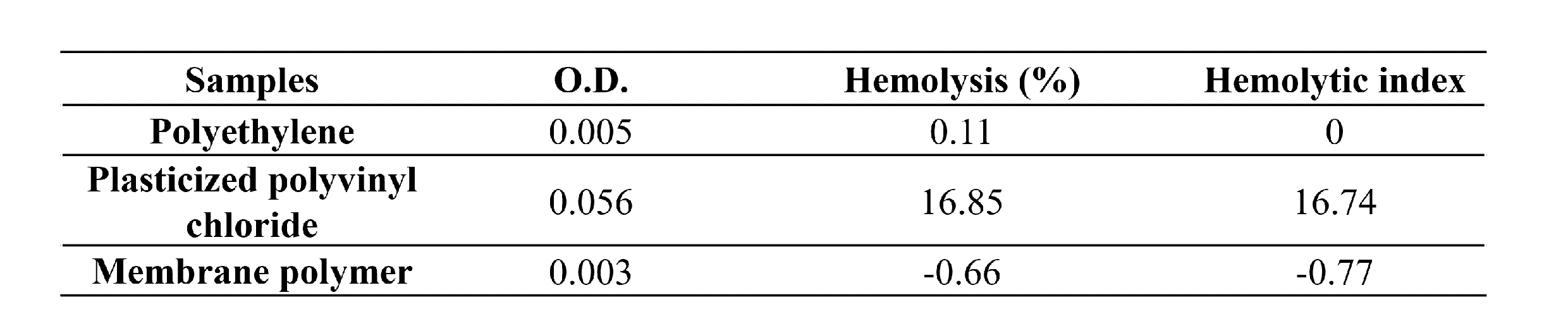

Supplement: Supplementary Materials — Figs. S1 to S13 Tables S1 to S10 Movies S1 to S5 [file research.0137.f1.zip › Table S8.png]

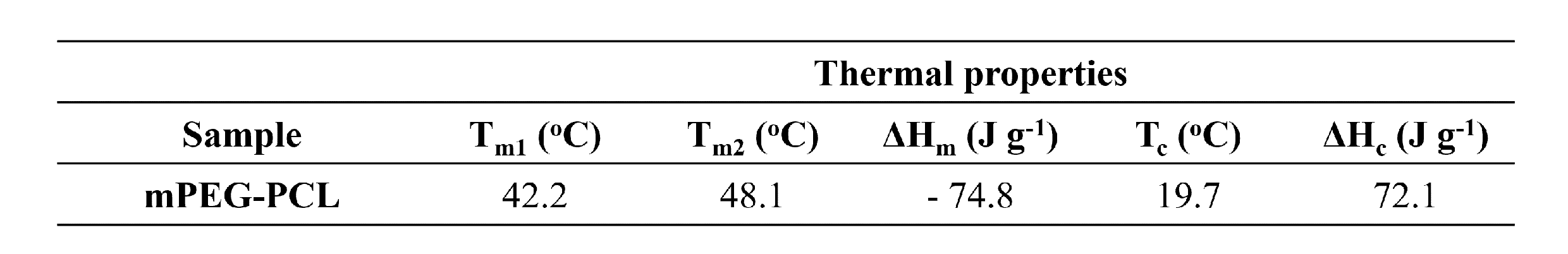

Supplement: Supplementary Materials — Figs. S1 to S13 Tables S1 to S10 Movies S1 to S5 [file research.0137.f1.zip › Table S9.png]

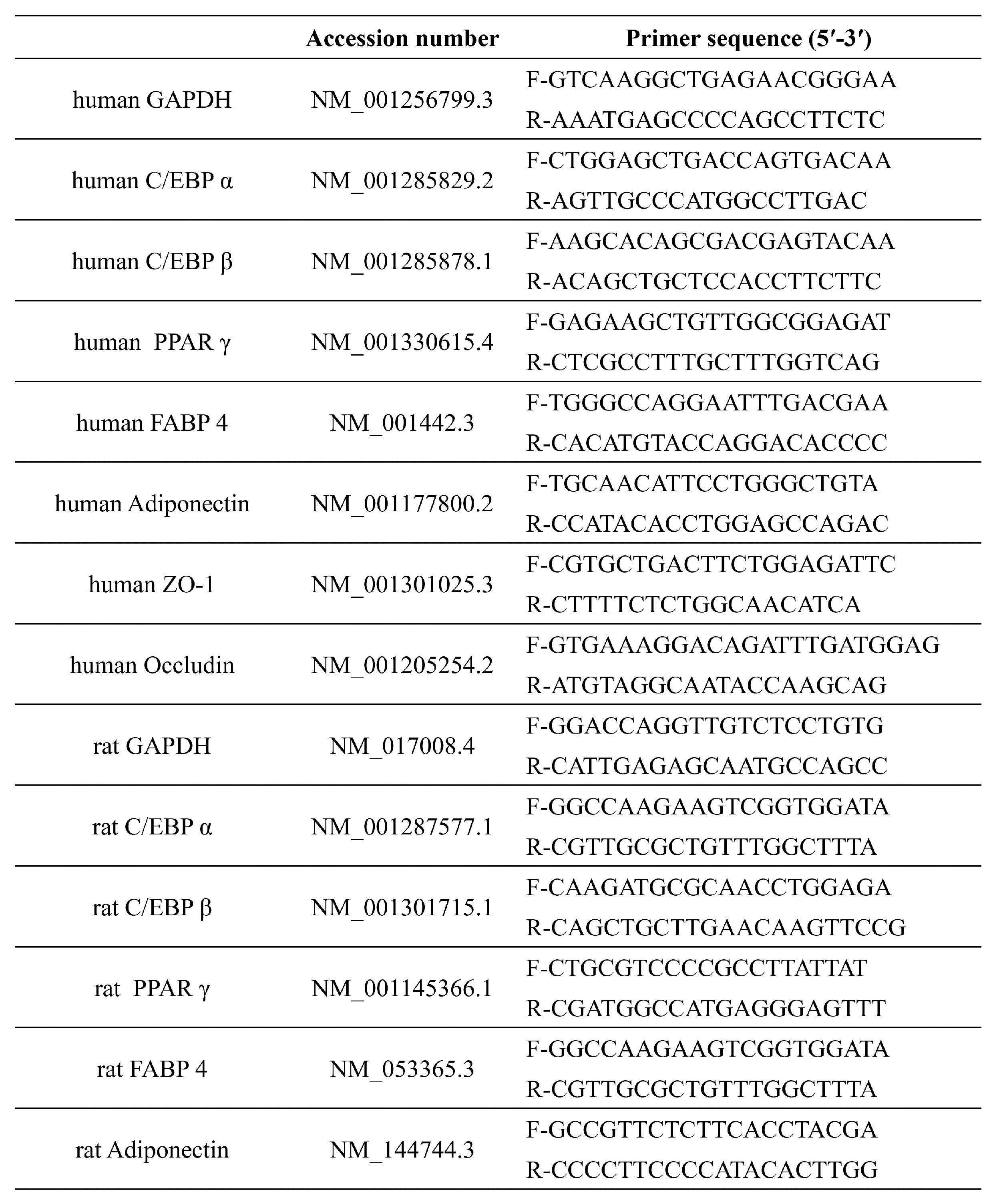

Supplement: Supplementary Materials — Figs. S1 to S13 Tables S1 to S10 Movies S1 to S5 [file research.0137.f1.zip › Table S10.png]
